# POET: A Software Suite for Mapping the Site-Specific Electronic Origins of Magnetic Anisotropy

**DOI:** 10.1021/acs.jpca.6c01818

**Published:** 2026-06-06

**Authors:** Jan Navrátil, Piotr Błoński

**Affiliations:** † Regional Centre of Advanced Technologies and Materials, Czech Advanced Technology and Research Institute (CATRIN), Palacký University Olomouc, Šlechtitelů 27, 779 00 Olomouc, Czech Republic; ‡ Department of Physical Chemistry, Faculty of Science, Palacký University Olomouc, tř. 17 listopadu 12, 779 00 Olomouc, Czech Republic; § IT4Innovations, VŠB−Technical University of Ostrava, 17. listopadu 2172/15, 708 00 Ostrava-Poruba, Czech Republic

## Abstract

The growing energy demand of global information technologies motivates the development of sustainable materials capable of retaining and processing information at the atomic scale. Resolving the site- and orbital-specific origins of magnetic anisotropy energy (MAE) is key to establishing the physical principles required for the rational design of tailored atomic-scale magnets. However, these contributions remain obscured in the output of noncollinear density functional theory calculations incorporating spin–orbit coupling. We present the Palacký OptoElectronic Toolkit (POET), an open-access suite that decomposes complex simulation data into intuitive graphical representations that map electronic reorganization onto atomic and orbital contributions and elucidates the interplay between bonding and magnetic anisotropy. To demonstrate its utility, we investigate two complementary strategies for achieving electric-field tunable MAE in transition-metal-functionalized graphene on various substrates. We show that iodination of Pt adatoms on nitrogen-decorated single-vacancy graphene on MgO creates strong, field-tunable in-plane anisotropy, while OsPt and OsPd heterodimers yield exceptional perpendicular MAE of ∼150 meV. The microscopic insights enabled by POET facilitate the rational control of magnetic anisotropy through chemical engineering, paving the way for energy-efficient information storage and processing at the atomic limit.

## Graphical abstract

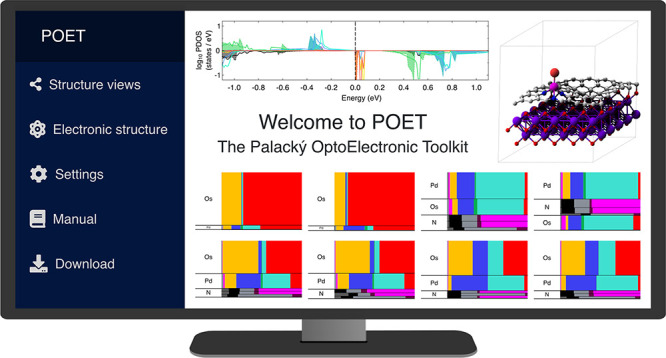

## Introduction

The rapid expansion of the information and communication technology (ICT) sector, together with its rising environmental impact^1–3^ and ever-increasing data storage demands,^4^ underscores the need for energy-efficient, high-density solutions. Miniaturizing magnetic bits to the atomic scale^5–10^ offers a compelling route toward this goal. Defective graphene, in particular, provides an ideal host for transition-metal-based (TM) atomic-scale magnets (ASMs),^11^ where the MAE can be chemically and structurally tuned with atomic precision.^12–14^

For practical implementation, graphene-supported ASMs must be integrated with solid substrates^15^ that preserve or enhance magnetic anisotropy.^16^ The successful synthesis of high-quality graphene monolayers on (111) metal surfaces such as Cu,^17,18^ Ni,^19^ and Ir,^20,21^ as well as on dielectric MgO,^22,23^ provides a realistic foundation for integrating atomic magnets into device architectures. Yet, how these substrates, especially when graphene contains defects,^24–29^ influence the electronic and magnetic properties of ASMs remains less explored.^16^

Second-order perturbation theory (PT2)^30,31^ offers a robust framework for interpreting the electronic origin of MAE^16^ by treating spin–orbit coupling (SOC) as a perturbation to the electronic structure from scalar-relativistic (SR) density functional theory (DFT). However, PT2 inherently neglects the rearrangement of electronic states under magnetization switching—an effect that becomes increasingly important in substrate-supported systems. Furthermore, PT2 can break down when the MAE contributions are dominated by near-degenerate transitions where the energy spacing between states is small relative to the SOC strength.^32^ Consequently, cross-validation against the force theorem (FT) or fully self-consistent noncollinear (SC NCL) SOC calculations is highly recommended in these regimes to ensure physical validity. However, the sheer complexity of the resulting electronic structure often obscures the specific orbital mechanisms driving the anisotropy.

In TM dimers, anisotropy originates primarily from SOC-driven modifications of the *d*-orbital projected density of states (PDOS) near the Fermi level (*E*_F_).^33^ To facilitate the analysis of these electronic-structure changes in complex systems, and to bridge the gap between the interpretative clarity of the PT2 framework and the quantitative accuracy of DFT–SOC results, we developed the Palacký OptoElectronic Toolkit (POET). This open-access software suite transforms raw simulation data into intuitive graphical representations that map the electronic reorganization induced by magnetization rotation onto atomic and orbital contributions, revealing how specific bonding motifs govern the MAE. Using this framework, we systematically investigate a comprehensive set of magnetic building blocks, including single TM atoms (Mn, Co, Ir, Pd, Pt), their halogen-functionalized counterparts (X–TM@NSV, X = F, Cl, B, I), and TM dimers incorporating Os and Ir, on both freestanding and substrate-supported nitrogen-decorated single-vacancy (NSV) graphene. Given that ASMs performance is governed by interfacial charge transfer and orbital hybridization, we analyze how these effects evolve across various TM@NSV architectures. We further demonstrate that the magnetic anisotropy can be modulated by an external electric field, revealing strong magnetoelectric coupling at the atomic scale.^34,35^ Finally, we employ POET to elucidate the physical mechanisms driving two distinct chemical strategies for achieving robust, tunable MAE: the iodination of Pt adatoms on MgO-supported NSV-graphene for high-stability in-plane anisotropy, and the formation of Os-based heterodimers (OsPt, OsPd) for perpendicular anisotropy on the same platform. Together, this work provides a versatile framework for the rational atomic-scale design of information storage and processing architectures.

## Theoretical Methods

### Density-Functional-Theory Calculations

All calculations were performed within spin-polarized density functional theory using the Vienna ab initio Simulation Package (VASP).^36–39^ Exchange–correlation effects were described by the generalized gradient approximation (GGA)^40,41^ in the Perdew–Burke–Ernzerhof (PBE) formulation.^42,43^ Core–valence interactions were treated within the projected augmented wave (PAW) method.^44,45^ A plane-wave basis with a kinetic-energy cutoff of 400 eV was used for both scalar-relativistic (SR) and NCL–SOC calculations—a value previously shown to yield converged energies for TM species bound to vacancy site in graphene.^11,46^ Increasing the cutoff to 500 eV resulted in MAE changes below 1 meV in tests on OsPd@NSV supported on Cu(111) and MgO(001). Partial occupancies were handled using Gaussian smearing with a width of 0.02 eV. Electronic self-consistency was achieved when the total-energy change fell below 10^–6^ eV, and structural relaxations proceeded until residual forces were smaller than 10^–2^ eV/Å. Dispersion interactions were included for halogenated systems and for those containing substrates using the DFT-D3 correction with Becke–Johnson damping.^47^

Brillouin-zone sampling was tailored to the lateral dimensions of each model system. For structural relaxations, a 6 × 6 × 1 Monkhorst–Pack *k*-mesh was used for freestanding NSV-graphene, while supported systems were relaxed with a 2 × 2 × 1 mesh (increased to 2 × 4 × 1 for the rectangular MgO(001) surface cell). Subsequent static SR calculations employed a 6 × 6 × 1 mesh for all structures. To ensure accurate MAEs, NCL–SOC calculations required denser sampling: 9 × 9 × 1 for freestanding and 3 × 3 × 1 for supported systems (2 × 4 × 1 for the MgO(001) surface). Convergence tests confirmed that increasing the mesh to 4 × 4 × 1 for OsPd@NSV@MgO(001) and to 6 × 6 × 1 for OsPt@NSV@Cu(111) altered the MAE by a maximum 3 meV.

Magnetic anisotropy energies were evaluated using NCL DFT with SOC.^48–51^ Owing to the high computational cost, MAEs were extracted from static total-energy calculations. The MAE was defined as

$$\mathrm{MAE}=\min(E_{x},E_{xy},E_{y})-E_{z}$$ 1

where *E*_α_ (α = *x*, *xy*, *y*, *z*) denotes the total energy for an initial magnetization aligned along direction α. Positive MAE values correspond to the *z*-axis (out-of-plane easy axis), taken perpendicular to the graphene plane and collinear with the TM–TM bond. Given that the standard PBE functional may underperform for molecular magnets,^52^ we tested the OptB86b functional^53–55^ for the freestanding I–Pt@NSV complex. The magnitude of the MAE increases by 17%, indicating that the predicted anisotropy is qualitatively robust with respect to the choice of exchange-correlation functional.

Atomic-scale magnets may exhibit a highly corrugated magnetic energy landscape, giving rise to multiple metastable spin isomers. To ensure that the correct electronic and magnetic ground state is identified for each magnetization direction, the NCL–SOC calculations were initialized from several distinct total magnetic moments. For instance, for freestanding OsPt@NSV under varying external electric field, the structure was first relaxed in three independent SR calculations with the total magnetic moment constrained to 1 μ_B_, 3 μ_B_, and 5 μ_B_. Each relaxed geometry then served as a starting point for a full NCL–SOC calculation. For the MAE evaluation, the lowest-energy solution obtained (independently for the easy- and hard-axis configurations) across all initial geometries and spin initializations was selected.

To evaluate the MAE in cases where standard self-consistent NCL–SOC relaxation from a chosen axis either led to nonmagnetic solution (e.g., freestanding I–Pt@NSV) or relaxed back to the easy magnetization direction (e.g., MgO(111)-supported systems), a constrained-moment protocol was employed that fixes the direction and/or magnitude of the magnetic moment. To minimize spurious contributions from the constraint, a stepwise procedure was adopted in which the penalty parameter (LAMBDA) was gradually increased (1, 7, and 50), with each step initialized from the converged charge density of the preceding one. The lowest-energy configuration was then taken as the relevant reference for that orientation.

Modeling graphene–substrate interfaces required consideration of lattice matching. Commensurate supercells were constructed by matching the graphene overlayer to the periodicity of each substrate (Figures 1, S1, and S2). For the hexagonal (111) metal surfaces the graphene in-plane lattice constant (2.47 Å) was left unstrained for Ni(111), expanded to 2.55 Å (3.2*%* strain) for Cu(111), and enlarged to 2.72 Å (10.1% strain) for Ir(111). For the MgO(111) surface two registry variants were considered: a *short match* (*sm*) with compressed graphene (2.40 Å, corresponding to a −2.8*%* strain) and a *long match* (*lm*) with slightly expanded graphene (2.50 Å, corresponding to a 1.2% strain). The MgO(111) in-plane constant used in both variants was 3.00 Å. The lattice constant of graphene on the rectangular MgO(001) surface was compressed to 2.45 (−0.8% strain) to align with the longer surface lattice vector (4.28 Å), while the shortest Mg–Mg spacing in this cell (3.02 Å) was comparable to the MgO(111) in-plane constant.

**Figure 1:**
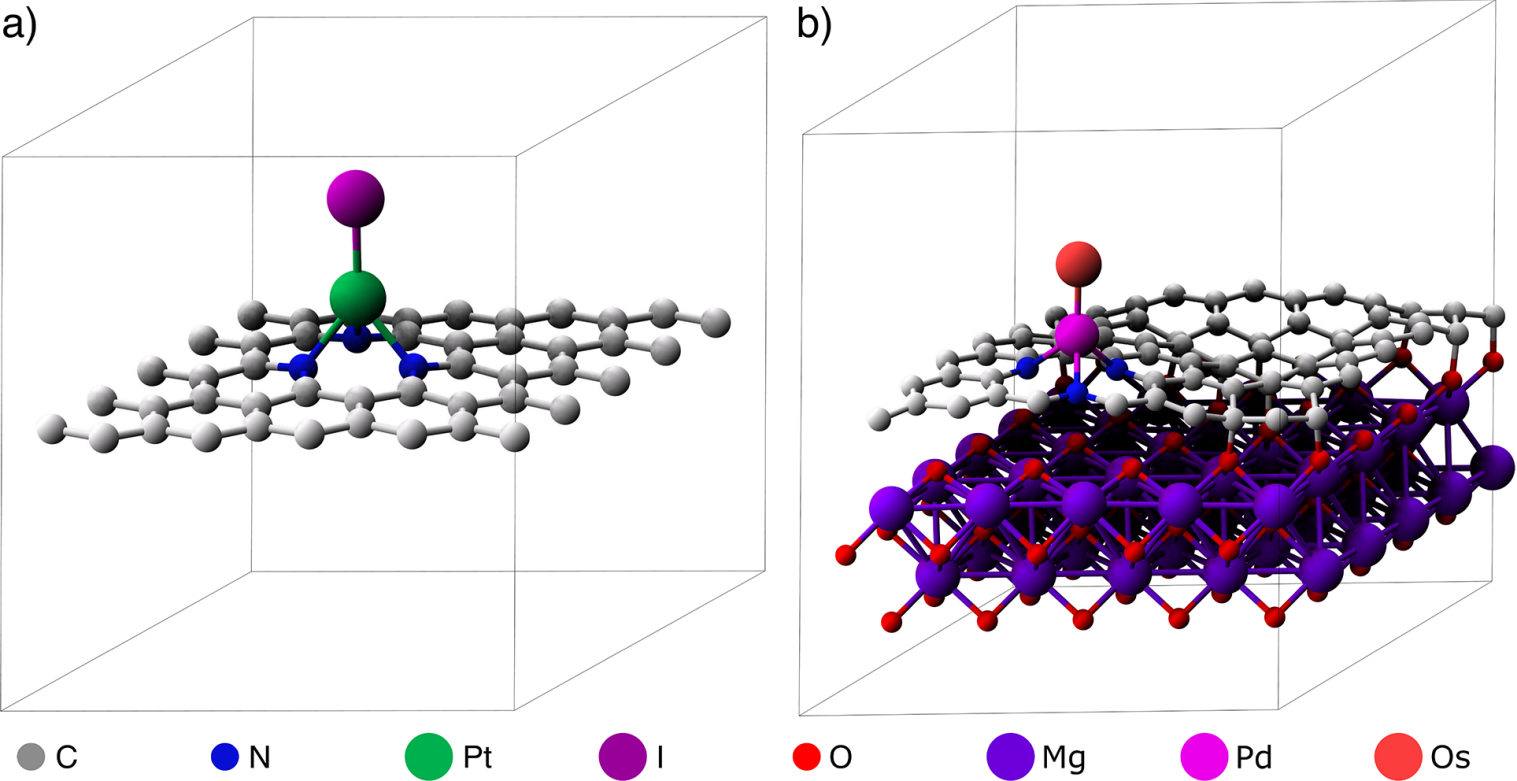
Selected supercells rendered using POET. (a) Freestanding I–Pt@NSV, (b) OsPd@NSV@MgO(111).

Metal substrates were modeled using four-layer slabs, with each layer containing a 5 × 5 arrangement of atoms (100 atoms in total). The MgO(001) substrate was represented by a four-layer slab with a 4 × 2 arrangement of MgO formula units (each containing two Mg and two O atoms) per layer, totaling 64 Mg and 64 O atoms. The *sm* MgO(111) slab contained 32 Mg and 48 O atoms with 4 × 4 atom arrangements per layer across 2 Mg and 3 O layers, supporting a 50-atom graphene sheet. The *lm* MgO(111) slab with 5 × 5 arrangements across 2 Mg and 3 O layers (50 Mg and 75 O atoms) supported a 72-atom graphene sheet. In all slab models, the bottom two layers were fixed at their bulk-truncated positions, while the upper layers—along with the graphene sheet and any adsorbates—were fully relaxed. A vacuum layer of >10 Å was inserted between periodic images for freestanding and metal–supported systems to eliminate spurious interactions. For the computationally demanding MgO-supported systems, the vacuum layer had to be reduced to >8 Å.

### Palacký OptoElectronic Toolkit (POET)

All electronic structure analyses and visualizations presented in this paper were performed using our in-house postprocessing software, the Palacký OptoElectronic Toolkit (POET), unless otherwise stated. POET was used to perform three central analyses of VASP results: (i) identifying bonding, nonbonding, and antibonding orbital character to estimate bond-order (BO); (ii) calculating orbital-pair contributions to the MAE via PT2; and (iii) interpreting magnetic FT results in terms of atom- and orbital-resolved MAE via the approximate reconstruction of band tracks and their projection character derived from *k*-point-resolved weights.

POET is developed in C# utilizing the Microsoft.NET 10 framework. Its architecture is divided into two complementary components: a back-end console application and a front-end web application. The console application processes the raw voluminous output files from VASP calculations, extracting and compiling the essential data into compact, portable files for efficient storage and transfer. The open-source back end allows users to develop custom import modules that generate a POET-compatible file format, enabling support for other electronic-structure packages without changes to the core analysis routines. Subsequently, users perform in-depth analysis through the universally accessible web application.

The web application is built using a Blazor WebAssembly framework with Razor pages syntax, enabling a dynamic, single-page experience that updates in response to user interactions. For 3D structural visualization (Figure 1), POET employs the babylon.js graphics engine, which provides a rich application programming interface (API) for all rendering tasks. The client-side communication between the Blazor application and babylon.js is facilitated by a custom JavaScript (JSInterop) wrapper inspired by Nek.^56^ The rendering approach implemented in POET, inspired by the Speck visualization library,^57^ incorporates graphical enhancements like ambient occlusion to improve spatial perception and image clarity.

The POET web application supports two deployment modes powered by WebAssembly to accommodate different computational workflows: a client-side (browser-only) mode and a server-hosted mode. In the former, POET is accessed at its web address, but downloaded and executed locally within a user’s web browser, with no subsequent communication to a server. While this approach provides complete platform independence and data privacy, all files must be manually selected through the browser interface due to standard client-side security sandboxing that prevents direct access to the local filesystem.

The server-hosted component must be downloaded and launched on the user’s local machine. This local server grants the web application secure access to browse files on the local drive or, via integrated SSH connections, on remote computers or clusters, eliminating the manual file selection step through the browser interface. While primarily designed for the local machine, with appropriate permissions, this server component would also allow users for direct results inspection on a cluster from any networked device, including smartphones. Planned future extensions of this mode include calculation preparation and job submission assistants.

### Console Application

#### Data Processing

The POET console application serves as the primary data-processing engine, efficiently parsing voluminous output files (e.g., VASP PROCAR) into compact, analysis-ready data sets (*PROCAR splitter* mode). Based on user-defined criteria, such as selecting atoms of interest and an energy window (e.g., ±5 eV around *E*_F_), the application extracts targeted information into separate files for each selected atom, achieving a significant reduction in file size while preserving all physical information within the chosen subset.

Specifically, the application executes user-configurable analytical routines, including (i) PT2-based calculations of MAE contributions (*PT2-to-MAE* mode, Figure 2), implementing the formalism presented in our recent work,^16^ (ii) an *orbital-occupancy calculator* allowing also for BO estimation and (iii) magnetic anisotropy analysis via the magnetic force theorem (*FT-to-MAE* mode). The output files are orders of magnitude smaller than the original raw files, enabling efficient storage and further exploration via the POET web application.

**Figure 2:**
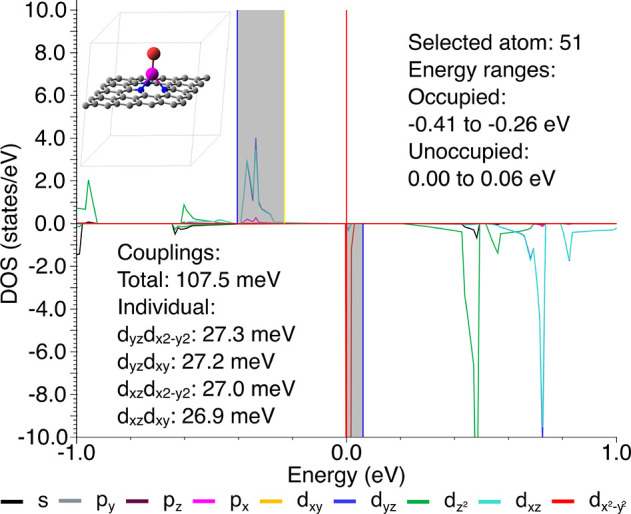
Interactive calculation of PT2 contributions to MAE using the POET web application. Selecting an atom in the rendered structure (inset) displays its SR-DOS, enabling the interactive selection of energy windows. The application computes site-resolved PT2 contributions using data sets preprocessed via the *PROCAR splitter* mode of the POET console application, with results displayed dynamically next to the structure. Energy boundaries are defined via mouse-based selection: within the occupied part of the SR-DOS, a left-click and Shift+click set the blue and yellow energy boundaries, respectively; for unoccupied states above the Fermi level, the same operations are performed while holding the Ctrl key.

To accommodate different user configurations, the console application is distributed in both framework-dependent and deployment-ready self-contained modes supporting major operating systems (Windows, Linux, and macOS), with the complete source code available on GitLab for custom compilation.

#### Orbital Occupancy

In the PAW method, PDOS-derived orbital occupations are not strictly particle-conserving. In practice, PAW codes calculate site- and orbital-resolved populations by projecting Kohn–Sham states onto atom-centered spherical harmonics within the PAW augmentation spheres. Charge residing in the interstitial region (outside these spheres) is not captured by these approximations, and numerical integration during postprocessing introduces additional errors. Consequently, the sum of orbital-projected occupations rarely equals the total valence electron count. Applying a *single global scaling factor* to the PDOS to enforce “charge conservation” implicitly assumes a uniform “leakage fraction” across all orbitals, which is rarely valid because the extent of the interstitial weight varies with the orbital character, the degree of hybridization, and the chosen sphere radii.

To address these limitations, the POET console application processes the orbital-projection weights stored in the PROCAR file. For every electronic state at each *k*-point, (i) the application retrieves its total occupancy and the corresponding set of raw projected atomic–orbital weights. (ii) These weights are normalized such that their sum matches the total Fermi–Dirac occupancy of that state, thereby accounting for the interstitial density based on the specific orbital character of that state. (iii) The corrected contributions are then scaled by the respective *k*-point weight and summed across all occupied bands and *k*-points, yielding the final spin-, orbital-, and atom-resolved occupancies. From these data, atom- and orbital-projected magnetic moments can be readily derived. By providing these resolved occupancies, the POET console application complements Bader charge partitioning. While Bader analysis captures the net charge transfer, POET reveals the underlying bonding mechanisms, such as electron back-donation.

Currently, these results are exported as text files, with future updates planned to integrate this analysis into the POET web application for interactive visualization.

#### Bond Order

The bond order is a conceptual measure of the number of chemical bonds between atoms. In the simplest diatomic molecular-orbital (MO) picture, it is related to the difference in occupation between bonding (B) and antibonding (AB) orbitals. Because the systems studied here are generally spin-polarized, we evaluate the BO in a spin-resolved manner, with all steps performed independently for each spin channel σ ∈ {↑, ↓}, and the total bond order is obtained as

$$\mathrm{BO}={\mathrm{BO}}_{↑}+{\mathrm{BO}}_{↓}$$ 2

Here, the bond order for a given spin channel is defined as

$${\mathrm{BO}}_{\sigma }=\frac{N_{e,B}^{\sigma }-N_{e,\mathrm{AB}}^{\sigma }}{2}$$ 3

where *N*_*e*,B_^σ^ and *N*_*e*,AB_^σ^ denote the *per-spin* electron counts in B and AB orbitals, respectively. While this formalism can be generalized to multicenter bonds, our focus here is on estimating the BO between two TM atoms using orbital-projected data from the VASP PROCAR file.

To estimate the BO in our periodic slab models, we employ the console application of POET in its *orbital-occupancy* mode. The user selects a pair of atoms (or a group of atoms treated as a single entity) and a threshold parameter *m*. POET processes spin-resolved Kohn–Sham states (weighted by their occupations), identifying a state (*n*, **k**, σ) as *MO-like* for the selected pair if the smaller projection weight satisfies *w*_min_ ≥ *m w*_max_. In this work, we use *m* = 0.50. For each such MO-like state, POET records its orbital-resolved occupancy and energy relative to *E*_F_ in a text output. States associated with the selected atoms that do not meet this bonding criterion are classified as *nonbonding* for the chosen pair; they may participate in other bonds but are not counted for the present TM–TM BO estimates.

In an idealized picture, the contribution of a single pair of orbitals (*o*) occupied by *N*_*e*_^σ^ per-spin electrons can be expressed as

$${\mathrm{BO}}_{o,\sigma }=\frac{1-|1-N_{e}^{\sigma }|}{2}$$ 4

reaching a maximum of 0.5 at *N*_*e*_^σ^ = 1 (fully occupied B and empty AB within a single spin channel) and drops to zero for *N*_*e*_^σ^ = 0 or 2. Summing over both spin channels recovers the maximum contribution of 1 for a spin-paired B orbital in the spin-symmetric limit. Such a clean decomposition is rarely possible in plane-wave calculations for low-symmetry systems like TM dimers anchored to a NSV in graphene with effective *C*_3*v*_ symmetry. We therefore adopt a simplified counting scheme inspired by the dimer-only picture.

For a late TM dimer (groups 8–10), up to five *d*-derived B and five AB combinations may form if all valence *d* orbitals of both TM atoms participate. In the spin-resolved convention, let *n* ^σ^ denote the effective number of nonbonding *d*-channels on the upper TM atom (TM_T_) in spin channel σ, computed as the sum of the per-spin occupancies (0–1) of the *d*-channels classified by POET as nonbonding for the selected TM–TM pair; thus 0 ≤ *n* ^σ^ ≤ 5.

To account for the *s* orbital, we introduce a binary correction *c* ∈ {0, 1} for the *s* channel: if the per-spin *s* occupancy exceeds 0.30, we treat *s* as nonbonding (*c* = 1); otherwise *c* = 0. The effective number of available MO pairs per spin is then approximated as

$$p^{\sigma }=5-(n^{\sigma }-c)$$ 5

With the total number of electrons *N*_*e*_^σ^ residing in all MO-like states identified between the two TM atoms, the BO is estimated for each spin channel as

$${\mathrm{BO}}_{\sigma }=\frac{p^{\sigma }-|p^{\sigma }-N_{e}^{\sigma }|}{2}$$ 6

Equation 6 can be generalized heuristically to account for additional atoms participating in the bonding network (e.g., the N atoms of the NSV defect) by introducing an effective participation number *a*,

$${\mathrm{BO}}_{a,\sigma }=\frac{\frac{p^{\sigma }a}{2}-\left|\frac{p^{\sigma }a}{2}-N_{e}^{\sigma }\right|}{a}$$ 7

In practice, however, POET currently identifies MO-like states only between the explicitly selected pair of atoms (or group of atoms). The nitrogen contributions are not included in the electron count *N*_*e*_^σ^. Hence, applying eq 7 amounts to a simplifying assumption about the availability of additional (effectively empty) orbitals on the N atoms. Testing with *a* = 3 (each N contributing 1/3) or *a* = 5 (each N contributing fully) yields similar trends. Using *a* = 3, the correlation coefficient between the computed BO and the adsorption energy of the upper TM atom in the dimers (*E*_*a*_^T^) is −0.42, whereas restricting the analysis to a strict two-atom picture (*a* = 2) gives −0.27. The imperfect correlation is expected; otherwise, it would imply that the adsorption energy is governed solely by the TM–TM BO.

The density of states (DOS) associated with the identified MOs can be approximately reconstructed by differentiating the cumulative MO-occupancy profile with respect to energy (Figures S3–S15). Within the POET console application, the cumulative occupancy–versus–energy curve is represented using a piecewise cubic Hermite interpolating polynomial (PCHIP) generated using the Math.NET Numerics library.^58^ This method yields a smooth, continuous representation, allowing the derivative, and thus the approximate MO-projected DOS to be evaluated at any energy point. The output resolution is controlled by the user via the number of points per eV.

The results are written to a Grace-formatted file for immediate visualization, and corresponding plotting templates are provided in the public POET repository. A native visualization workflow is planned for future integration into the POET web application to reduce reliance on external plotting tools.

#### Orbital-Decomposed MAE

The POET console application reconstructs electronic band connectivity by tracing energy eigenstates across the Brillouin zone using the atom- and orbital-resolved projection weights from the VASP PROCAR file. As a preliminary step, the raw projection weights for each state (i.e., the atom- and orbital-resolved contributions) are linearly normalized to unity, following the routine described above for the *orbital-occupancy calculator*. This mitigates nonconservation of PAW-sphere projections and provides numerical consistency for the subsequent band-matching procedure.

Next, for uniform meshes, the *k*-points are ordered starting from Γ, with subsequent points arranged using a rectangular spiral (four-directional) algorithm. This traversal minimizes the *k*-space step between consecutive points, which improves the robustness of the iterative connectivity reconstruction.

In each iteration, POET performs a batch evaluation of all possible assignments between currently unassigned states at the new **k** point and existing partially reconstructed band tracks. The suitability score for each candidate is computed by comparing the new projection against the last *n* projections within a given band track (*n* is user-defined; *n* = 3 is typically effective), using four weighted criteria: (i) similarity in atomic and orbital character, (ii) similarity in electronic occupation, (iii) energy proximity to the most recent matched state, and (iv) deviation from the energy trajectory extrapolated from the last *n* matched points.

Each criterion can be tuned by two adjustable parameters: (i) a nonlinearity exponent applied to the corresponding deviation measure, controlling how strongly small vs large mismatches affect the score and (ii) a relative weight that balances the importance of the criteria, with a default value of 1 being satisfactory for most cases. Setting any single weight above 10 causes it to dominate the suitability score, overshadowing the contributions of the remaining criteria.

After suitability scores are computed for all candidate pairs, POET assigns the highest-scoring state–track pair and removes it from the pool. This process iterates until no remaining scores exceeds a user-defined threshold (0.1 is the default value). To avoid forced, low-suitability assignments, users can inspect the resulting band connectivity in the POET web application, where unassigned projections are marked with red dots (Figure S16), adjust parameters, and rerun the procedure until a physically sensible connection pattern is obtained. When focusing on particular atomic species, such as the TMs central to this work, increasing their weight in the character-similarity criterion promotes chemically relevant assignments.

The reconstructed band tracks, together with their atom/orbital character, enable an orbital-decomposed analysis of MAE within the magnetic FT framework.^59,60^ First, the scalar-relativistic ground-state (SR GS) electronic structure is obtained. Noncollinear SOC calculations are then performed on this fixed charge density for magnetizations oriented along the Cartesian directions *x*, *y*, *z*, and the in-plane diagonal *xy*. The MAE is evaluated from band-energy differences between a perpendicular (⊥) and parallel (∥) orientation relative to the dimer bond as

$$\mathrm{MAE}\approx \sum_{k}w_{k}\left[\sum_{n}f_{nk}^{⊥}\epsilon_{nk}^{⊥}-\sum_{n}f_{nk}^{∥}\epsilon_{nk}^{∥}\right]$$ 8

where *w*_**k**_ are the *k*-point weights and *f*_*n* **k**_^α^ are the occupations for orientations α ∈ {⊥, ∥}. While the total MAE does not require state-by-state pairing, an orbital-resolved decomposition does; therefore, POET reconstructs band tracks for each orientation and then establishes a cross-orientation mapping using an alignment algorithm analogous to the *k*-point connectivity procedure. Users can manually connect pairs in the web interface if any automated alignment is incorrect.

### Web Application

The POET web application serves as an interactive front end for visualizing atomic geometries and analyzing data generated by the console application.

All graphics (except structure plots) are exportable as Scalable Vector Graphics (SVG), allowing manual adjustment of fonts and colors. 3D atomic models (Figure 1) are rendered as raster images and exported by default as PNG files, offering a balanced compromise between visual fidelity and file size. The output resolution is scalable, with dimensions of 5000 × 3000 pixels or greater being readily achievable.

Visualization styles, rendering options, and plot parameters are configured via the *settings* panel (Figure 3). For instance, the *general settings* tab (Figure 3a) allows users to select the font, panel labeling style, and plot-display mode. This mode toggles between a layout optimized for interactive on-screen viewing and a more compact layout intended for exported figures.

**Figure 3:**
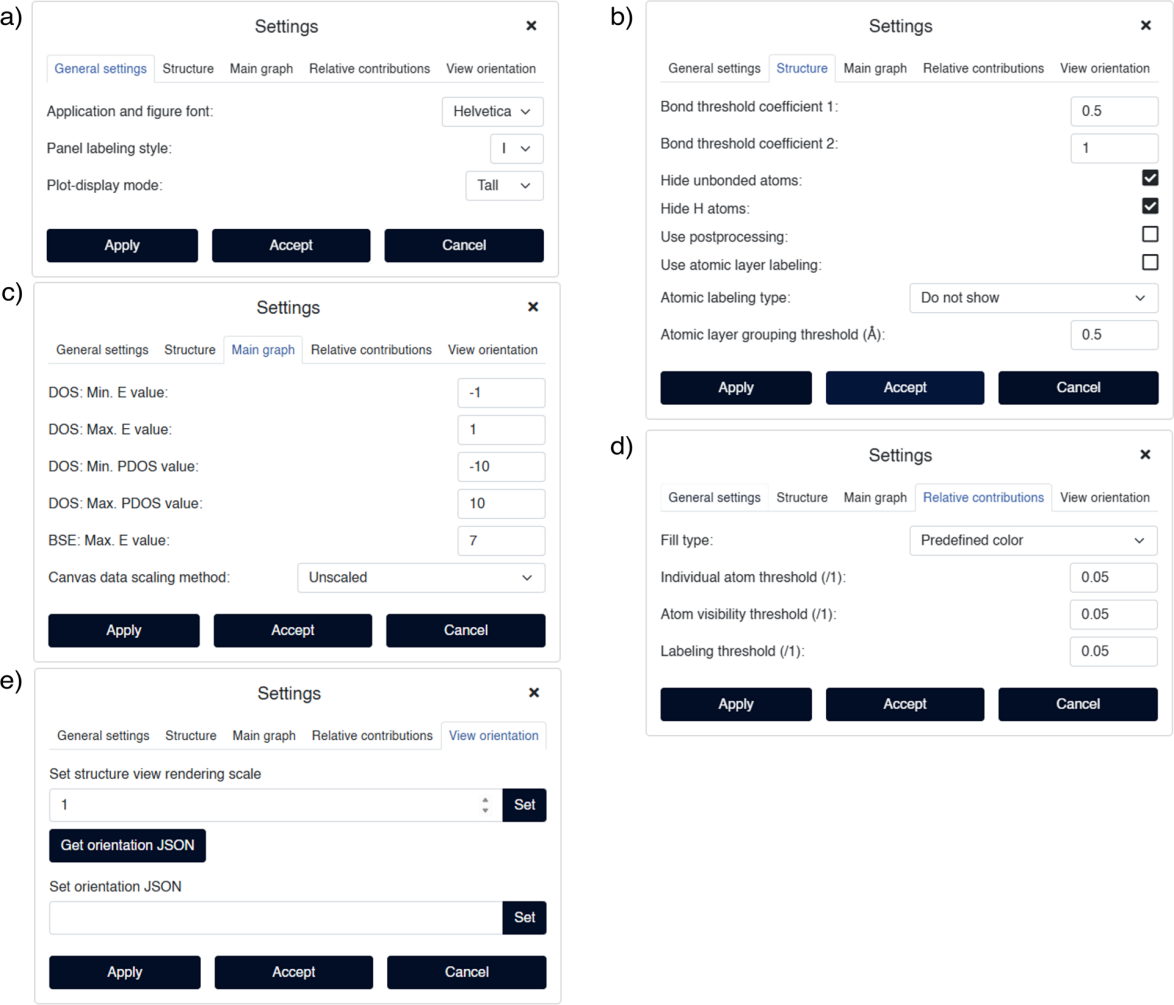
Settings tabs in the POET web application. The panels correspond to (a) General settings, (b) Structure, (c) Main graph, (d) Relative contributions, and (e) View orientation.

The application provides extensive control over the visualization of atomic structures (Figure 3b). This includes: (i) the automated determination of bonds via a user-adjustable maximum distance between atomic pairs,

$$d_{\max}=(r_{1}+r_{2}+c_{1})\times c_{2}$$ 9

where *r*_1_ and *r*_2_ are covalent radii, and *c*_1_ and *c*_2_ are bond threshold coefficients (default *c*_1_ = 0.5 and *c*_2_ = 1.0), (ii) options to hide isolated or hydrogen atoms, and (iii) the automatic grouping of atoms by element within a user-defined *z*-coordinate tolerance given in Å (i.e., within a layer). Further, atomic labeling can be configured to follow the input order, element type, or orbital contribution magnitude (controlled by a user-defined *labeling threshold*).

Electronic structure data, such as the density of states (Figure 2) or band structure (Figure S16), are displayed in a central interactive plot. The visible ranges on both the *x* and *y* axes are adjustable (Figure 3c), with the values on the *y*-axis scalable via a square root or a decadic logarithm. This plot allows users to select states of interest, which are then visualized in accompanying relative contribution plots where atomic radii are scaled by their contribution magnitude. Interactive features include selecting an energy range by holding the Shift key, which updates the relative plots to show averaged values for that interval, and defining a custom reference energy by holding the Ctrl key, after which all values are reported relative to this user-defined point.

A core analytical feature of the POET web application is the 2D visualization of system properties—such as the magnetic anisotropy or excited charge distribution—decomposed into their relative atomic and orbital contributions. In this layout, a user-selected set of electronic states is depicted by a series of rectangles. The filled area, height, and width of each rectangle are proportional to the total contribution of a given state, the contribution from a specific atom or group of atoms, and the orbital character, respectively (e.g., Figure 4c–j). These contribution data are simultaneously mapped onto the 3D structure plot, where the size of each atomic sphere is proportional to its contribution.

**Figure 4:**
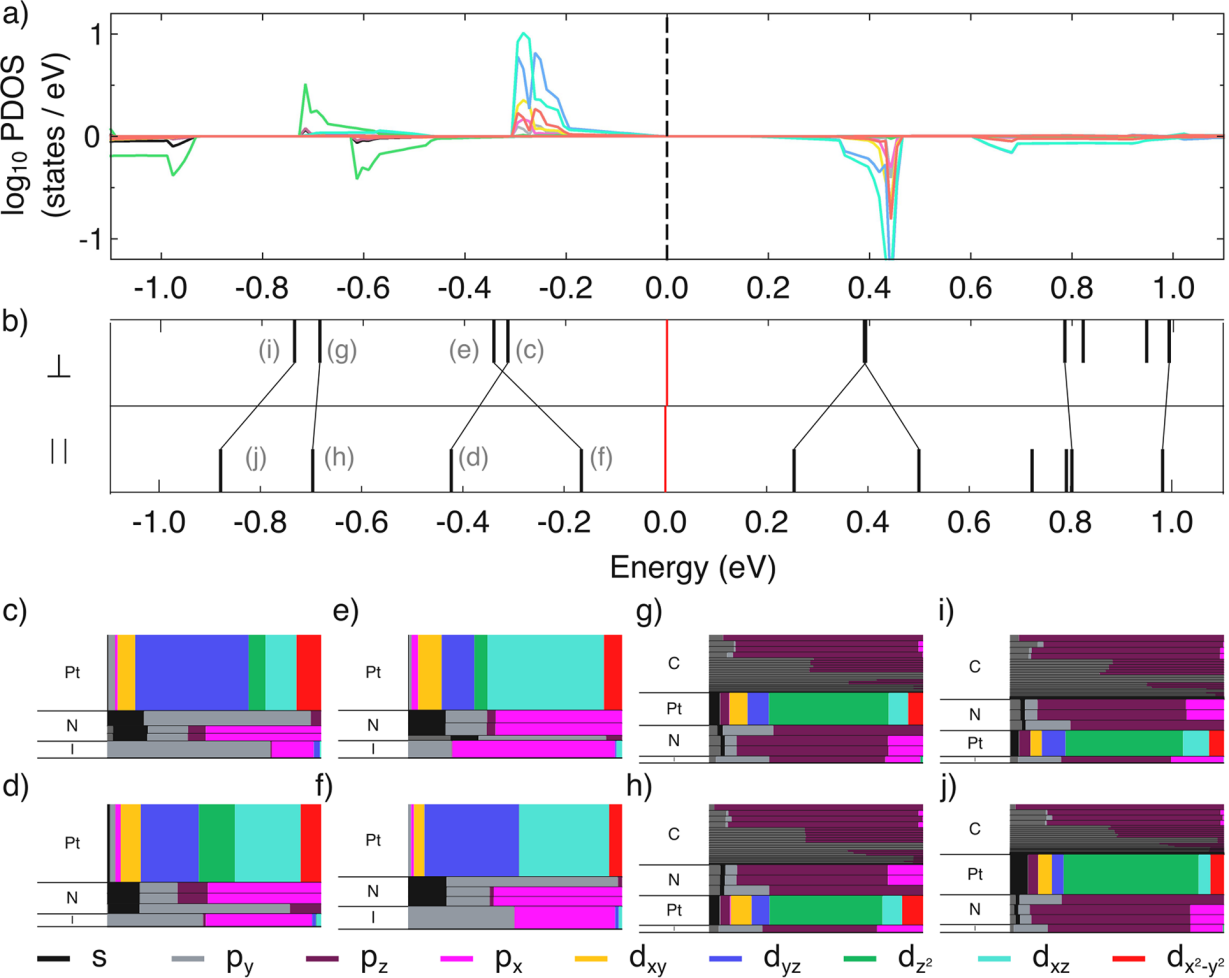
Origin of the large magnetic anisotropy energy (MAE) in freestanding I–Pt@NSV, analyzed via the magnetic force theorem (FT) using POET. (a) Scalar-relativistic projected density of states (PDOS) of the Pt atom. (b) eigenvalue spectra for magnetization perpendicular (top) and parallel (bottom) to the I–Pt bond, each aligned to its Fermi level (*E*_F_, red lines). For a direct comparison, the energy axes are aligned to account for the difference in *E*_F_. Letter labels (c–j) in panel (b) mark the states with the dominant contribution to the MAE, with the corresponding atom- and orbital-resolved decompositions displayed in panels (c–j), where the box sizes are proportional to the orbital weights.

The appearance of the contribution plots is user-configurable using either a predefined color scheme or patterns (Figure 3d). Additionally, three adjustable thresholds control the level of detail. (i) *Individual atom threshold*: An atom whose contribution exceeds this value is displayed as a separate entity. (ii) *Atom visibility threshold*: Contributions from atoms or groups of atoms below this value are omitted to prevent clutter, while their combined share is represented as blank space. (iii) *Labeling threshold*: Only contributions exceeding this value are annotated. Setting this threshold above the *atom visibility threshold* allows significant features to be identified without overcrowding the plot with labels.

Settings for the structure view and orientation (Figure 3e) allow users to upscale the rendering resolution for high-quality exports and to save precise viewing angles as JSON files for later reuse.

## Results and Discussion

### Halogenation

Direct halogenation of the graphene framework is energetically disfavored (Table S4). Unlike F,^61,62^ Cl, Br, and I remain weakly physisorbed and endothermic relative to diatomic X_2_ molecules, even at NSV defects.

In contrast, halogenation of TM atoms anchored at NSV sites is strongly exothermic (Table S5, Figure S17a) and remains energetically favorable relative to X_2_ across the entire TM@NSV set. Multihalogen adsorption (2–3 atoms, Figure S18) becomes progressively less favorable but retains its exothermic character (Table S5), suggesting that the degree of functionalization can be tuned via controlled halogen exposure.

To assess substrate effects, we selected the X–Pt@NSV (X = Cl and I) systems exhibiting the highest MAE and examined them on representative supports (Table S6). On Cu(111), Ni(111), and MgO(001), the binding strength is only slightly reduced (by up to 7%), whereas Ir(111) and MgO(111) induce a much stronger suppression (by as much as 55% for I–Pt@NSV@Ir(111)). This trend correlates with the preadsorption Bader charge on Pt (*R* = 0.77), such that a greater positive charge on Pt reduces electron density available for Pt–X bond formation, thereby weakening halogen binding.

### TM Dimers

TM–TM dimerization (Figure S20) proceeds via the strongly exothermic adsorption of a second metal atom (TM_T_) onto a TM preanchored at an NSV site (TM_B_) (see SI Section S5, Table S8 and Figure S17b). Most dimers adopt upright geometries, whereas tilted configurations occur only on MgO(111), where interactions with defect-boundary atoms quench magnetism; such structures are excluded from further analysis.

The OsPd@NSV system illustrates how the symmetry reduction at the NSV defect enables unconventional orbital couplings that govern both bonding and magnetism. Its high-spin GS (5.00 μ_B_) lies 0.46 eV below the low-spin configuration (1.00 μ_B_) and is stabilized by spin-down σ_*s*,*d*_ hybrids between Pd *d*_*z* ^2^_ and Os *d*_*z* ^2^_ and *s* orbitals (Figures S3 and S21). In the spin-up channel, a coupling between Pd *d*_*xy*_ *d*_*x* ^2^–*y* ^2^_ and Os *d*_*yz*_ *d*_*xz*_, forbidden in *D*_4*h*_ or *C*_4*v*_ symmetry, becomes allowed via the NSV-induced *C*_3_ symmetry, which prompts a ∼30° in-plane reorientation of the Pd *d*_*xy*_ *d*_*x* ^2^–*y* ^2^_ lobes while keeping *d*_*yz*_ *d*_*xz*_ saturated by N coordination. The highest occupied spin-up state thus comprises two degenerate antibonding π_*p*,d_^*^ orbitals delocalized across N, Pd, and Os, while Os *d*_*xy*_ and *d*_*x* ^2^–*y* ^2^_ form sharp degenerate states just above *E*_F_ in the spin-down channel.

Chemical substitutions systematically tune this bonding framework by altering valence electron counts. Substituting Pt for Pd, for instance, reduces the *s*–*d* separation, increasing *s*-orbital occupancy and pushing the spin-up π_*p*,d_^*^ manifold above *E*_F_, lowering antibonding filling (Figure S4). Concurrently, Os *d*_*x* ^2^–*y* ^2^_ in the spin-down channel moves below *E*_F_, stabilizing a low-spin GS (1.33 μ_B_). Even in the high-spin state (5.00 μ_B_, 0.25 eV above the GS), π_*p*,d_^*^ remains unoccupied (Figure S5)—a feature that critically influences the MAE.

On supported NSV-graphene, the *E*_*a*_^T^ values generally remain within ±20% of freestanding limits, except on Ir(111) and MgO(111) (Table S8). On Cu(111) and Ni(111), partial lone-pair donation from N to the substrate depletes dimer electron density, reducing spin-up antibonding and spin-down bonding occupancies, and yielding BO changes of ±12 and ±25%, respectively (Figures S8–S11). Slightly higher BO values on Ni(111) correlate with a more exothermic average adsorption energy (defined here as the mean *E*_*a*_^T^ for all considered dimers on a given surface). On MgO(001), the alignment of a single N atom above a Mg^2+^ site localizes lone-pair donation, limiting BO changes to between −6 and +16% (Figures S12–S15). In contrast, Ir(111) induces strong TM_B_–Ir interactions, reducing BO by up to 60% and *E*_*a*_^T^ by as much as 68%. On MgO(111), however, the effects are strongly system dependent. To illustrate, *E*_*a*_^T^ strengthens by 60% for OsPt but drops by two-thirds for OsCo. Crucially, both experience severe BO reductions, indicating that substrate-induced variations stem primarily from interfacial charge redistributions that modify the local electrostatic potential rather than intrinsic TM_T_–TM_B_ bonding alterations.

Finally, TM–TM dimerization generally reduces the TM_B_ magnetic moment, consistent with previous reports^11,46^ (Table S8). High-spin configurations persist mainly for dimers featuring Co and Mn anchors, reflecting the robust moment retention typical of 3*d* transition metals.

### Magnetic Anisotropy Energy

Single TM adatoms on freestanding NSV-graphene typically exhibit negligible MAEs (<1 meV, Table S9). The only exception is Ir@NSV, which reaches −10 meV with in-plane easy axis. PT2 analysis attributes this anisotropy to spin-down coupling between the occupied *d*_*z* ^2^_ and the unoccupied *d*_*yz*_ *d*_*xz*_ (Figure S22), while the corresponding spin-up *d*_*yz*_ *d*_*xz*_ states are filled and thus blocked from contributing a positive MAE term.

When substrate-supported, most TM@NSV systems remain effectively isotropic (Table S10); even the modest anisotropy of Ir@NSV is reduced to at most −5 meV on MgO(001). Only Pt@NSV@MgO(111) (Figure S23) stands out, developing a substantially larger MAE of −25 meV. Structural distortion of graphene induced by MgO(111), with Pt occupying a protruding position, is not sufficient to account for this effect, as recomputing the same distorted geometry without the substrate reverses the MAE to +8 meV, indicating that the large negative anisotropy originates from substrate-driven electronic reorganization rather than from structural deformation alone. C–O bonding at the interface perturbs the graphene π network and promotes further charge transfer from Pt to the N atoms, as reflected by an increased Bader charge on Pt from +0.46 e to +0.68 e. The charge transfer fully depopulates the highest-lying spin-down *d*_*yz*_ *d*_*xz*_ states, with the magnetization-direction-dependent energy shift of SOC-split *d*_*yz*_ *d*_*xz*_ states as the main contribution and smaller contributions from *d*_*xy*_ *d*_*x* ^2^–*y* ^2^_ (Figure S24).

Chemical functionalization of TM atoms anchored at NSV sites provides an alternative route to large anisotropy (Figure S25, Table S11). Particularly, iodine functionalization of Pt@NSV yields a record-high MAE of −53 meV, arising primarily from second-order spin-flip SOC between occupied and unoccupied *d*_*yz*_ *d*_*xz*_ states (Figure S26). When the magnetization aligns along the I–Pt bond, the two highest occupied states exhibit mixed Pt *d*_*yz*_ *d*_*xz*_ and iodine *p*_*y*_ *p*_*x*_ character (Figure 4). The lower of the two also contains a *d*_*z* ^2^_ component, stabilized along the magnetization axis and accompanied by reduced iodine character. Rotating the magnetization perpendicular to the bond merges these states into nearly degenerate levels that lie below the center of gravity of their parallel-orientation counterparts. Concurrently, the axial stabilization of *d*_*z* ^2^_ is lifted, and its character redistributes into higher-lying, nearly degenerate *d*_*yz*_ *d*_*xz*_ states. This asymmetric orbital response to magnetization reorientation lowers the total energy of the perpendicular configuration by 61 meV, giving rise to the large negative MAE.

Applying an external electric field provides a distinct mechanism for tuning the MAE.^34,35^ Unlike Pt@NSV@MgO(111), where the MAE shows at most a 36*%* reduction from its zero-field baseline, I–Pt@NSV exhibits a near-monotonic field dependence, reaching −71 meV at −0.5 eV/Å (Figure S27a). For the field range from −0.5 to +1.0 eV/Å, the energetic separation between the occupied and unoccupied *d*_*yz*_ *d*_*xz*_ states progressively increases, weakening their PT2 contribution to the MAE (Figure S28). A similar trend is observed for the Pd analogue, where MAE values are roughly halved, consistent with its smaller SOC constant.

In contrast, iodination of Pt@NSV@MgO(111) suppresses the MAE, which is accompanied by a reduction in the total magnetic moment, consistent with an iodine-induced increase in Pt-state occupations (as in freestanding Pt@NSV), but here predominantly in the spin-down channel. Together, the iodine and the MgO(111) substrate disrupt the characteristic Pt *d*_*yz*_ *d*_*xz*_ hybridization, consistently preserved in all other Pt@NSV systems, by leaving *d*_*yz*_ below *E*_F_ and pushing *d*_*xz*_ above it (Figure S29). While the coupling between occupied *d*_*yz*_ and empty *d*_*xz*_ within the same spin channel promotes an easy axis along the I–Pt bond, this contribution is modest and serves primarily to mitigate the negative MAE. However, an external electric field can partially counteract this suppression, restoring the spin-flip *d*_*xz*_–*d*_*yz*_ coupling and an MAE of −3 meV at −0.50 eV/Å and −30 meV at +0.50 eV/Å.

Achieving large, positive MAE values, however, requires changing the fundamental structural unit, from halogenated single atoms to TM dimers anchored at the NSV sites (Table S12).^11,16,46^ Among freestanding systems, OsPd@NSV exhibits the largest MAE (133 meV) identified in this study. PT2 analysis attributes this anisotropy to spin-flip coupling between the occupied spin-up π_*p*,d_^*^ and unoccupied spin-down *d*_*xy*_ *d*_*x* ^2^–*y* ^2^_ states near *E*_F_ (Figure S30d).^16^ Their importance is further validated by varying the TM–TM bond length (Figure S31). The MAE peaks at 213 meV when the bond is compressed to 90% of its equilibrium distance and decreases to 8 meV at 115%, which originates from a shift of the spin-up Os–Pd π_*p*,d_^*^ states relative to *E*_F_, moving them to lower energies as the bond elongates (Figure S30). This analysis not only resolves the discrepancy with our earlier, higher reported MAE value^11,16^ but also highlights the corrugated nature of the potential energy surface near the GS.

Magnetic force theorem (FT),^59,60^ combined with the POET band-track reconstruction, further clarifies the orbital origin of the MAE. With magnetization perpendicular to the Os–Pd bond, the highest-occupied/lowest-unoccupied molecular orbital (HOMO/LUMO) correspond to π_*p*,d_^*^ and the degenerate nonbonding *d*_*xy*_ and *d*_*x* ^2^–*y* ^2^_ pair, respectively (Figure 5). Aligning the magnetization along the dimer bond lifts this degeneracy and reorders the frontier levels, lowering the total energy: HOMO–1 becomes a δ-bonding *d*_*xy*_ *d*_*x* ^2^–*y* ^2^_ hybrid with ∼30% Pd (including *d*_*yz*_ and *d*_*xz*_) and N (*s* and *p*) character; the HOMO comprises an antibonding π* orbital dominated by Os/Pd *d*_*yz*_ *d*_*xz*_ with ∼25% N *p* mixing; the LUMO is antibonding π_*p*,d_^*^ (Os/Pd); and LUMO+1 is a degenerate *d*_*xy*_ and *d*_*x* ^2^–*y* ^2^_ pair with dominant Os weight.

**Figure 5:**
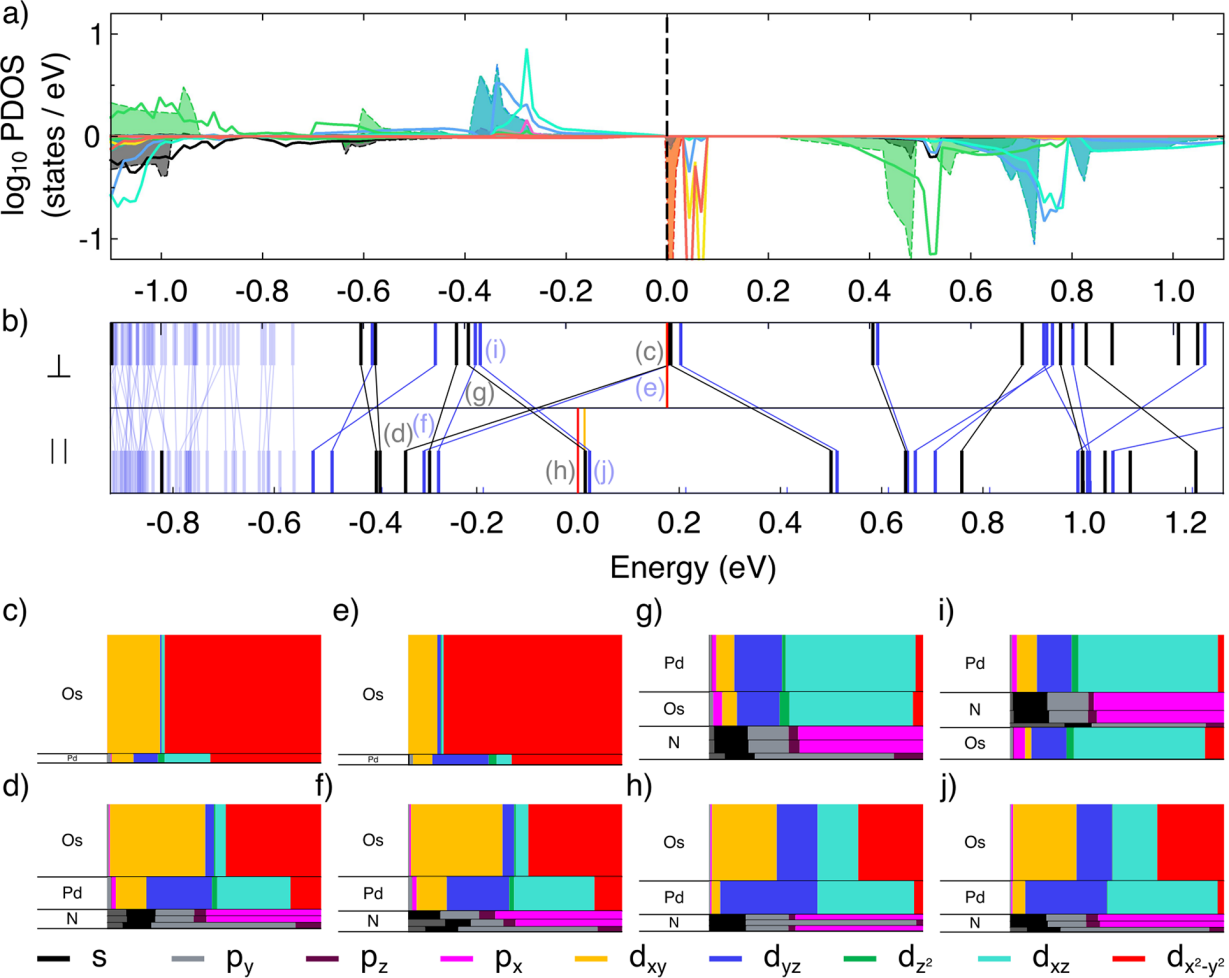
Origin of the large magnetic anisotropy energy (MAE) in freestanding OsPd@NSV and OsPd@NSV supported on MgO(001), analyzed via the magnetic force theorem (FT) using POET. (a) Scalar-relativistic projected density of states (PDOS) of the Os atom, where darker dashed lines correspond to freestanding OsPd@NSV and lighter solid lines to OsPd@NSV@MgO(001). (b) eigenvalue spectra for magnetization perpendicular (top) and parallel (bottom) to the Os–Pd bond, each aligned to its Fermi level (*E*_F_, red and orange lines). Black lines indicate freestanding OsPd@NSV, blue lines the MgO-supported system. For a direct comparison, the energy axes are aligned to account for the difference in *E*_F_. Letter labels (c–j) in panel (b) mark the states with the dominant contributions to the MAE, with the corresponding atom- and orbital-resolved decompositions displayed in panels (c,d,g,h) for the freestanding and (e,f,i,j) MgO-supported OsPd@NSV, where the box sizes are proportional to the orbital weights.

Beyond OsPd@NSV, the magnitude and sign of the MAE across the 3*d*–5*d* dimer series is governed by the relative energy and occupation of the frontier π_*p*,d_^*^ and *d*_*xy*_ *d*_*x* ^2^–*y* ^2^_ orbitals. Systems where the key π_*p*,d_^*^–*d*_*xy*_ *d*_*x* ^2^–*y* ^2^_ coupling channel is maximized (OsPd@NSV and OsPt@NSV (Figure S32)), yield the largest MAE (>100 meV). If these states are pushed away from *E*_F_ or competing (opposite-sign) couplings appear, the MAE falls to 67–50 meV (OsCo@NSV, OsIr@NSV, IrPd@NSV) or less (IrCo@NSV, IrIr@NSV), or can invert sign (OsMn@NSV, IrMn@NSV) (Table S12).

Under a varying electric field from −1.00 to +0.50 eV/Å, the MAE of freestanding OsPd@NSV increases monotonically from 62 to 155 meV (Figure S27b). Negative fields upshift the LUMO (*d*_*xy*_ *d*_*x* ^2^–*y* ^2^_), weakening its coupling to π_*p*,d_^*^, whereas positive fields strengthen this coupling by raising the energy of the π_*p*,d_^*^ states (Figure S33).

In contrast, the MAE of OsPt@NSV exhibits a monotonic increase from 67 to 140 meV under a field of opposed polarity and remains nearly unchanged between −0.25 and −0.75 eV/Å (Figure S27b). Within this plateau, a field-induced transition from a low-spin state (∼3 μ_B_) to a high-spin state (∼5 μ_B_) occurs, accompanied by a reorganization of the frontier orbitals (Figure S34): the spin-up π_*p*,d_^*^ states become occupied and the spin-down *d*_*xy*_ *d*_*x* ^2^–*y* ^2^_ states empty at −0.75 eV/Å, with their energy separation remaining nearly constant, resulting in an electronic configuration that mirrors that of OsPd@NSV at zero-field.

Substrate adsorption typically quenches the zero-field MAE of OsPd@NSV by 60 meV or more. Only the MgO(111) and MgO(001) surfaces reduce the MAE by a mere 3 and 5 meV, respectively, correlating with the degree of perturbation of the electronic structure. For instance, the energy separation of the two highest occupied bands upon rotating the magnetization from perpendicular to parallel magnetization decreases from 177 meV in the freestanding system to 167 meV on MgO(001) (Figure 5), while the essential orbital mechanism responsible for the large MAE in the freestanding system remains largely preserved. Under a field of +0.25 eV/Å, the MAE increases to 140–150 meV (Figure S27b).

In sharp contrast, OsPt@NSV exhibits a substantial MAE enhancement, from 120 to 151 meV, upon adsorption on MgO(001). Here, asymmetric coordination of N atoms by Mg^2+^ lifts the degeneracy of the spin-up π_*p*,d_^*^ states: $\pi_{p,d_{yz}}^{*}$ , oriented toward Mg^2+^, is stabilized below *E*_F_, while $\pi_{p,d_{xz}}^{*}$ , associated with the two more distant N atoms, remains above *E*_F_ (Figure S13). This selective stabilization preserves the positive OsPd@NSV-like contribution and introduces an additional term from $\pi_{p,d_{yz}}^{*}$ to $\pi_{p,d_{xz}}^{*}$ (occupied–unoccupied) coupling. Under a field of +0.25 eV/Å, the MAE further increases to 155 meV.

## Conclusions

Giant magnetic anisotropies can be realized in graphene-based heterostructures through two complementary chemical strategies. First, Os adatoms anchored to Pd- and Pt-decorated NSV defects in MgO-supported graphene produce exceptionally large perpendicular MAE, corresponding to predicted ten-year blocking temperatures^46,63^ of approximately 30–40 K, placing these systems nearly on par with state-of-the-art Ho@MgO single-atom magnets^7,9,64^ and well above lanthanides directly adsorbed on graphene.^15,65^ Although such heterostructures might be challenging to prepare experimentally, their extremely high projected bit density (440 TBit/in.^2^) and a strong magnetoelectric response (enabling in situ control of the energy barrier without compromising long-term thermal robustness) are particularly compelling. Second, the chemical functionalization of single Pt adatoms provides a more experimentally accessible route to large, field-tunable anisotropy, as the chemistry required for single-atom halogenation is simpler to implement than atomically precise dimer assembly. Notably, these halogenated species with in-plane easy axes can enable alternative device paradigms, including low-dissipation spin transport,^66^ ultrasensitive magnetometry,^67^ and probabilistic spin logic.^68^

Central to these findings is the POET suite, which elucidates the physical mechanisms governing magnetic behavior through the magnetization-dependent mapping of electronic reorganization onto intuitive, graphical representations of atomic and orbital contributions to the magnetic anisotropy. This open-access tool not only resolves the origin of MAE in current systems but also establishes the framework for the rational atomic-scale design of future information storage architectures.

## Supplementary Material



## Data Availability

Data supporting the findings of this paper are provided in the Supporting Information. Both the paper and the Supporting Information, as well as all data contained therein are freely available at 10.5281/zenodo.20321242. The Palacký OptoElectronic Toolkit (POET) web application is freely available for noncommercial use at https://poet.run. The code for the console application of POET is available at https://gitlab.com/navrsoft/poet_public.

## Notes

Structural, energetic, electronic, and magnetic characteristics of the investigated systems; and magnetic anisotropy energies and corresponding electronic-structure plots (PDF)
