# Supplementary material for: POET: A Software Suite for Mapping the Site-Specific Electronic Origins of Magnetic Anisotropy
Source: J Phys Chem A. 2026 Jun 6;130(24):4723–33. doi: 10.1021/acs.jpca.6c01818 (PMC13288674; doi:10.1021/acs.jpca.6c01818)
Supplement: Supplementary file 1 [file jp6c01818_si_001.pdf]

# Supporting Information for: POET: A Software Suite for Mapping the Site-Specific Electronic Origins of Magnetic Anisotropy


Jan Navrátil[1,2*] and Piotr Błoński[1,3*]

[1] *Regional Centre of Advanced Technologies and Materials, Czech Advanced Technology and Research Institute (CATRIN), Palacký University Olomouc, Šlechtitelů 27, 779 00 Olomouc, Czech Republic*

[2] *Department of Physical Chemistry, Faculty of Science, Palacký University Olomouc, tř. 17 listopadu 12, 779 00 Olomouc, Czech Republic*

[3] *IT4Innovations, VŠB–Technical University of Ostrava, 17. listopadu 2172/15, 708 00 Ostrava-Poruba, Czech Republic*

∗ Email: jan.navratil@upol.cz; piotr.blonski@upol.cz

# Contents


S1 Graphene on surfaces S3

S2 Bond-order analysis in POET S8

S3 Band-structure reconstruction for FT-MAE analysis in POET S21

S4 Halogenation S22

S5 TM adatoms S27

S6 TM dimers S31

S7 Magnetic anisotropy energy S39

# S1 Graphene on surfaces

Graphene–substrate interactions range from physisorption on Cu(111) [1] and MgO(001) [2], through weak chemisorption on Ni(111) [3], to covalent bonding on Ir(111) and MgO(111) [4] (Table S1). Introducing an NSV defect (Table S2, Figures S1–S2) strengthens graphene binding by 0.02–0.09 eV/atom, due to covalent N–surface interactions. The resulting binding hierarchy (Ir(111) $<$ Cu(111) $<$ MgO(001) $<$ Ni(111) $<$ MgO(111)) mirrors that of pristine graphene but with substrate-specific deviations.

On Ni(111), N–Ni bonding is counterbalanced by an increased average graphene–surface separation (from 2.21 Å to 2.44 Å, Table S3), resulting in a negligible net gain in binding strength. On MgO(001), the flat geometry and nearly unchanged spacing similarly hinder any strengthening of graphene adhesion. In contrast, on Ir(111), in-plane relaxations, outward from the NSV, enhance binding by 0.03 eV/atom. The most pronounced enhancement, 0.09 eV/atom, occurs on MgO(111) due to a combination of in-plane relaxations directed toward the NSV and out-of-plane corrugations. When the NSV is laterally displaced, this effect diminishes, underscoring the role of N–O bonding in governing graphene–oxide adhesion.

**Table S1: Structural and energetic characteristics of pristine graphene on the investigated substrates.** The graphene lattice constant $a$, graphene–substrate distance $d$, and average vertical separation $d_{vgs}$ (all in Å). The binding energy per carbon atom (in eV), the total charge of the graphene sheet $q_{\mathrm{G}}$, and the average charge on the graphene atoms $\overline{q_{\mathrm{G}}}$ (both in e). Available literature values are provided for comparison.

| | Cu (111) | Ni (111) | Ir (111) | MgO(S) (111) | MgO(L) (111) | MgO (001) |
|---|---|---|---|---|---|---|
| $a$ | 2.55 | 2.47 | 2.72 | 2.40 | 2.50 | 2.45 |
| | 2.55$^{a}$ | 2.49$^{b}$ | | 2.40$^{c}$ | | |
| $d$ | 3.18 | 2.21 | 2.04 | 1.4–3.0 | 1.5–2.6 | 3.22 |
| | 3.11$^{a}$ | 2.30$^{b}$ | | 1.5–2.9$^{c}$ | | 3.80$^{d}$ |
| $d_{vgs}$ | 3.18 | 2.21 | 2.04 | 2.77 | 2.07 | 3.22 |
| $\overline{E_b}$ | −0.02 | −0.12 | 0.26 | −0.18 | −0.20 | −0.06 |
| | −0.04$^{a}$ | −0.11$^{b}$ | | −0.05$^{c}$ | | −0.00$^{d}$ |
| $q_{\mathrm{G}}$ | −0.36 | −2.02 | −2.10 | 5.04 | 7.79 | −0.23 |
| $\overline{q_{\mathrm{G}}}$ | −0.01 | −0.04 | −0.04 | 0.10 | 0.11 | 0.00 |

*a* [1], *b* [3], *c* [4], *d* [2]

**Table S2: Energetic characteristics of NSV–graphene on the investigated substrates shown in Fig. S1 and Fig. S2.** The binding energy of the NSV–graphene sheet $E_b$, the binding energy normalized per atom $\overline{E_b}$, and the corresponding change in binding energy per atom associated with NSV defect formation $\Delta\overline{E_b}$ (all in eV).

| | Cu (111) | Ni (111) | Ir (111) | MgO $(111)_{sm}$ | MgO $(111)_{lm}$ | MgO (001) |
|---|---|---|---|---|---|---|
| Configuration | H | H | B | H | H | |
| $E_b$ | −1.65 | −5.60 | 10.97 | −13.27 | −16.98 | −3.55 |
| $\overline{E_b}$ | −0.03 | −0.11 | 0.22 | −0.27 | −0.24 | −0.06 |
| $\Delta\overline{E_b}$ | −0.02 | 0.01 | −0.03 | −0.09 | −0.03 | 0.00 |
| Configuration | T | T | H | T | T | |
| $E_b$ | −1.59 | −5.38 | 15.21 | −9.67 | −14.81 | |
| $\overline{E_b}$ | −0.03 | −0.11 | 0.31 | −0.20 | −0.21 | |
| $\Delta\overline{E_b}$ | −0.01 | 0.01 | 0.06 | −0.02 | 0.00 | |
| Configuration | D | B | T | E | E | |
| $E_b$ | −1.51 | −5.18 | 16.68 | −9.16 | −14.79 | |
| $\overline{E_b}$ | −0.03 | −0.11 | 0.34 | −0.19 | −0.21 | |
| $\Delta\overline{E_b}$ | −0.01 | 0.01 | 0.09 | −0.01 | 0.00 | |

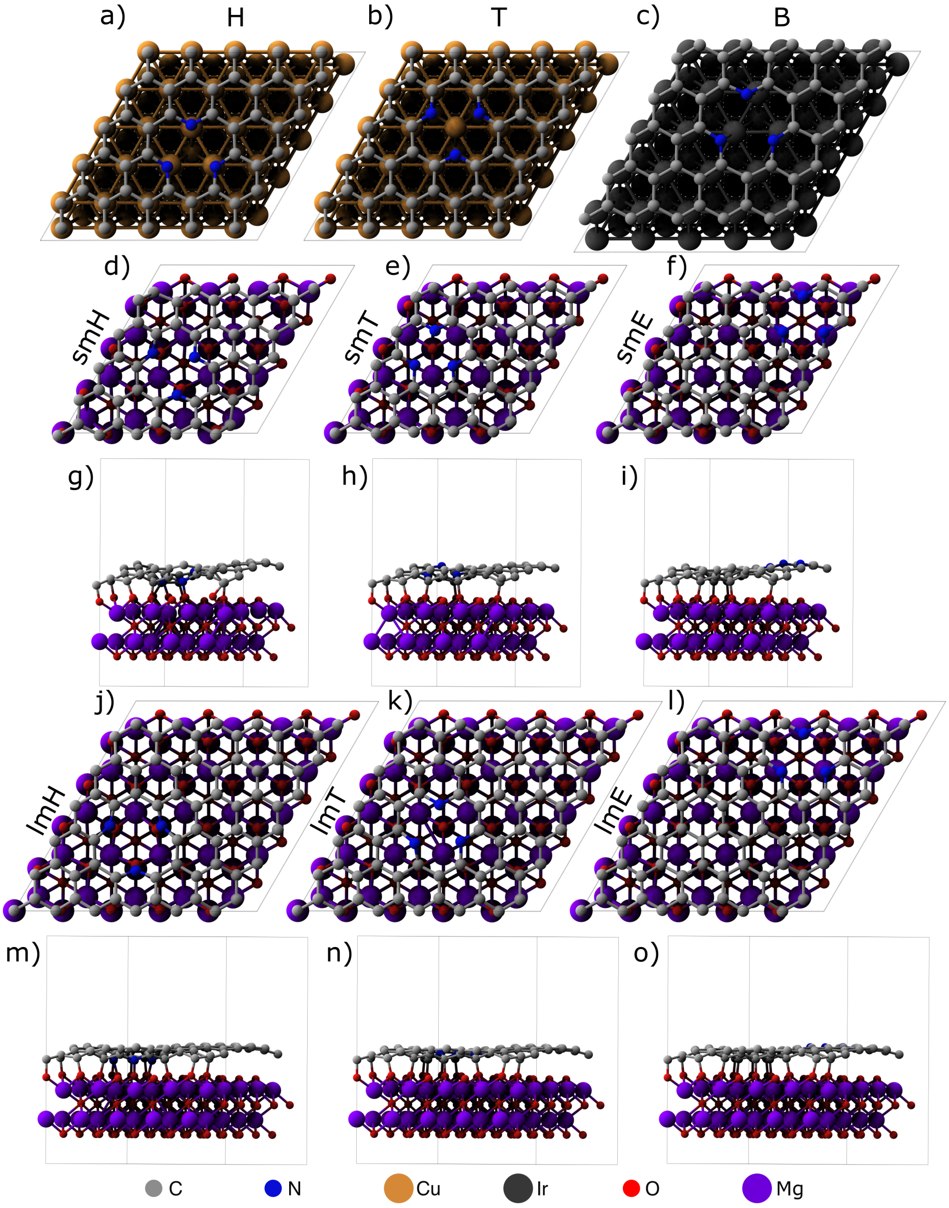


**Figure S1: High-symmetry adsorption geometries of NSV–graphene on the investigated substrates. (a–c)** Top views of representative configurations on a metal (111) surface: **(a)** the hollow site (H), where the center of the NSV defect is positioned above the three-fold hollow of the surface atoms; **(b)** the top site (T), with the defect center above a surface atom (both are stable on Cu and Ni surfaces); and **(c)** the bridge site (B), where surface atoms align with the graphene bridge position (the most stable configuration on the Ir surface). **(d,e)** Top views of the H and T configurations, respectively, on the *short-match* (sm) MgO(111) surface. **(f)** The NSV defect is placed in a region of graphene that is significantly elevated (E) from the surface due to graphene buckling. **(g–i)** Side views of the configurations depicted in **(d–f)**. **(j–o)** *Long-match* (lm) MgO(111) surface (cf. **(d–i)**).

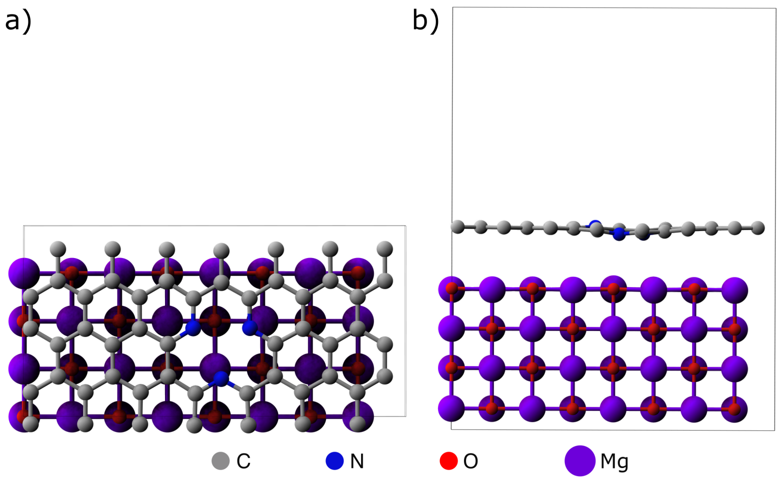


**Figure S2: Adsorption geometry of NSV–graphene on the MgO(001) surface.** The lattice mismatch precludes high-symmetry configurations. **(a)** Top view and **(b)** side view.

**Table S3: Scalar-relativistic characteristics of NSV–graphene on the investigated substrates.** The average N-N distance $d_{\mathrm{NN}}$, the average distance between each N atom and its nearest surface atom $d_{\mathrm{Ns}}$, the average vertical distance between the N atoms and all graphene atoms $d_{v\mathrm{Ng}}$, the average vertical distance between the N atoms and the surface atoms $d_{v\mathrm{Ns}}$, and the average vertical distance between the NSV–graphene and the substrate $d_{v\mathrm{gs}}$ (all in Å). The graphene buckling amplitude, defined as the vertical separation between the atoms in the NSV–graphene with the maximum and minimum $z$-coordinates (in Å). The total magnetic moment of the system relative to the corresponding pristine-graphene system $\mu_{\mathrm{red}}$ and the sum of the local magnetic moments of all N atoms $\mu_{\mathrm{N}}$ (both in $\mu_{\mathrm{B}}$). The Bader charge of the NSV–graphene sheet $q_{\mathrm{g}}$, the average Bader charge per atom within the NSV–graphene sheet $\overline{q_{\mathrm{g}}}$, and the average Bader charge of the N atoms $\overline{q_{\mathrm{N}}}$ (all in e). See Figure S1 for the notation.

| Substrate | $d_{\mathrm{NN}}$ | $d_{\mathrm{Ns}}$ | $d_{v\mathrm{Ng}}$ | $d_{v\mathrm{Ns}}$ | $d_{v\mathrm{gs}}$ | $d_{ba}$ | $\mu_{\mathrm{red}}$ | $\mu_{\mathrm{N}}$ | $q_{\mathrm{g}}$ | $\overline{q_{\mathrm{g}}}$ | $\overline{q_{\mathrm{N}}}$ |
|---|---|---|---|---|---|---|---|---|---|---|---|
| NSV | 2.62 | | | | | 0.37 | 0.90 | 0.25 | | | −1.12 |
| Cu | 2.85 | 2.22 | −0.31 | 2.35 | 2.67 | 0.38 | 0.00 | 0.00 | −1.45 | −0.03 | −1.10 |
| Ni | 2.67 | 2.04 | −0.37 | 2.08 | 2.44 | 0.82 | 1.57 | 0.04 | −1.75 | −0.04 | −1.16 |
| Ir | 3.12 | 2.23 | 0.06 | 2.11 | 2.05 | 0.07 | −0.02 | 0.00 | −2.32 | −0.05 | −0.87 |
| $\mathrm{MgO(111)_{smH}}$ | 2.80 | 1.80 | −0.49 | 1.72 | 2.21 | 1.96 | −3.84 | 0.00 | 5.49 | 0.11 | −0.80 |
| $\mathrm{MgO(111)_{smT}}$ | 2.51 | 2.63 | 0.01 | 2.30 | 2.29 | 1.55 | 2.02 | 0.66 | 4.74 | 0.10 | −1.02 |
| $\mathrm{MgO(111)_{smE}}$ | 2.56 | 3.41 | 0.67 | 2.94 | 2.27 | 1.66 | −0.37 | −0.07 | 4.95 | 0.10 | −1.06 |
| $\mathrm{MgO(111)_{lmH}}$ | 3.21 | 1.36 | −0.75 | 1.40 | 2.15 | 1.16 | 0.50 | −0.03 | 7.26 | 0.10 | −0.61 |
| $\mathrm{MgO(111)_{lmT}}$ | 2.77 | 2.38 | −0.08 | 1.99 | 2.07 | 1.08 | 1.86 | 0.94 | 7.80 | 0.11 | −0.75 |
| $\mathrm{MgO(111)_{lmE}}$ | 2.73 | 3.02 | 0.43 | 2.52 | 2.09 | 1.10 | 0.35 | −0.01 | 7.71 | 0.11 | −1.03 |
| MgO(001) | 2.61 | 3.17 | −0.16 | 3.04 | 3.20 | 0.38 | 0.30 | 0.13 | −0.28 | −0.01 | −1.14 |

## S2 Bond-order analysis in POET

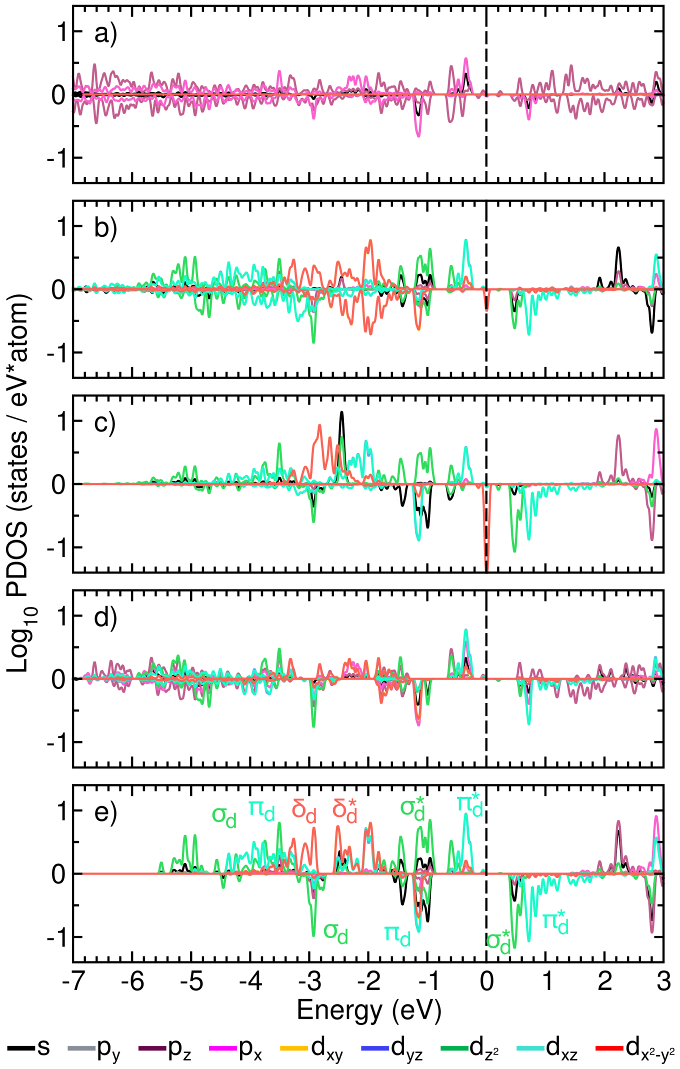


**Figure S3: Bond-order analysis for OsPd@NSV based on eigenstate-reconstructed PDOS using POET.** Reconstructed atomic PDOS for **(a)** the three N atoms forming the NSV defect ($N_3$), **(b)** Pd, **(c)** Os. **(d,e)** Molecular orbitals reconstructed from hybridized states between **d** $N_3$ and Pd, and **(e)** Pd and Os.

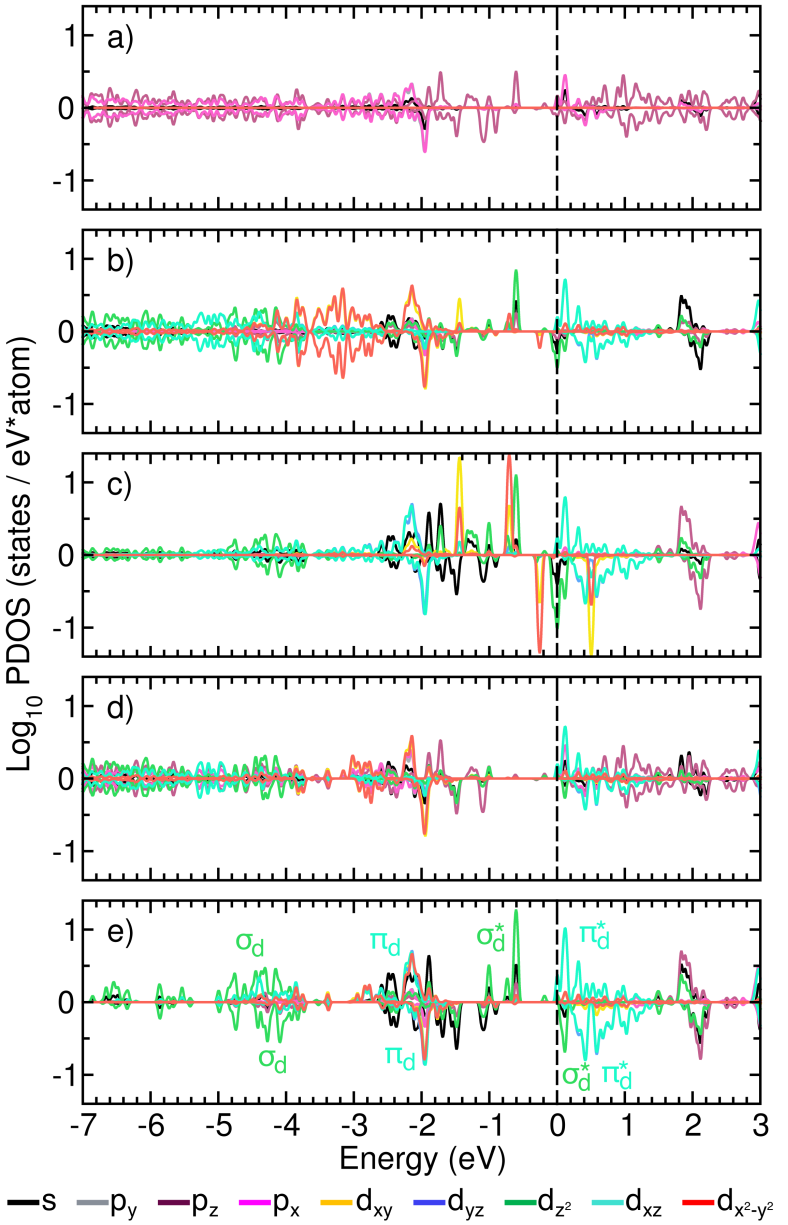


**Figure S4: Bond-order analysis for OsPt@NSV based on eigenstate-reconstructed PDOS using POET.** Reconstructed atomic PDOS for **(a)** the three N atoms forming the NSV defect ($N_3$), **(b)** Pt, **(c)** Os. **(d,e)** Molecular orbitals reconstructed from hybridized states between **(d)** $N_3$ and Pt, and **(e)** Pt and Os.

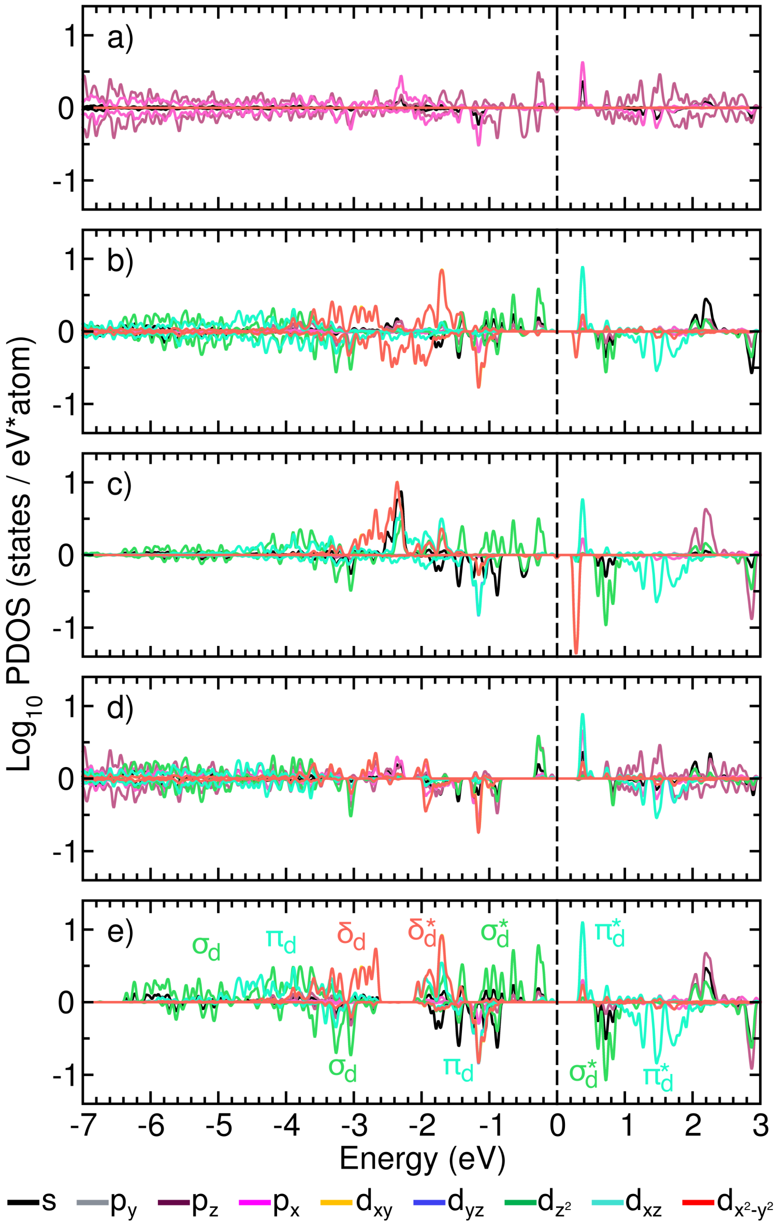


**Figure S5: Bond-order analysis for OsPt@NSV with the total magnetic moment of the supercell constrained to 5.00 $\mu_B$, based on eigenstate-reconstructed PDOS using POET.** Reconstructed atomic PDOS for **(a)** the three N atoms forming the NSV defect ($N_3$), **(b)** Pt, **(c)** Os. **(d,e)** Molecular orbitals reconstructed from hybridized states between **(d)** $N_3$ and Pt, and **(e)** Pt and Os.

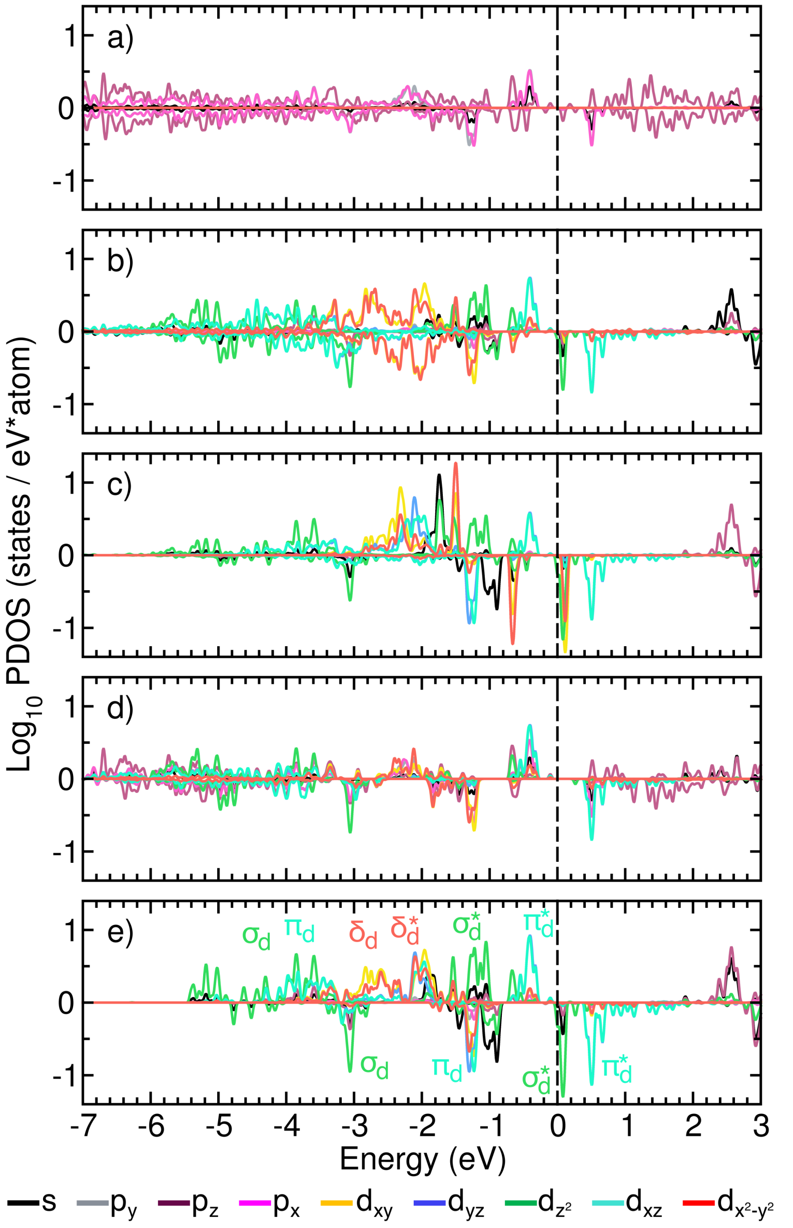


**Figure S6: Bond-order analysis for IrPd@NSV based on eigenstate-reconstructed PDOS using POET.** Reconstructed atomic PDOS for **(a)** the three N atoms forming the NSV defect ($N_3$), **(b)** Pd, **(c)** Ir. **(d,e)** Molecular orbitals reconstructed from hybridized states between **(d)** $N_3$ and Pd, and **(e)** Pd and Ir.

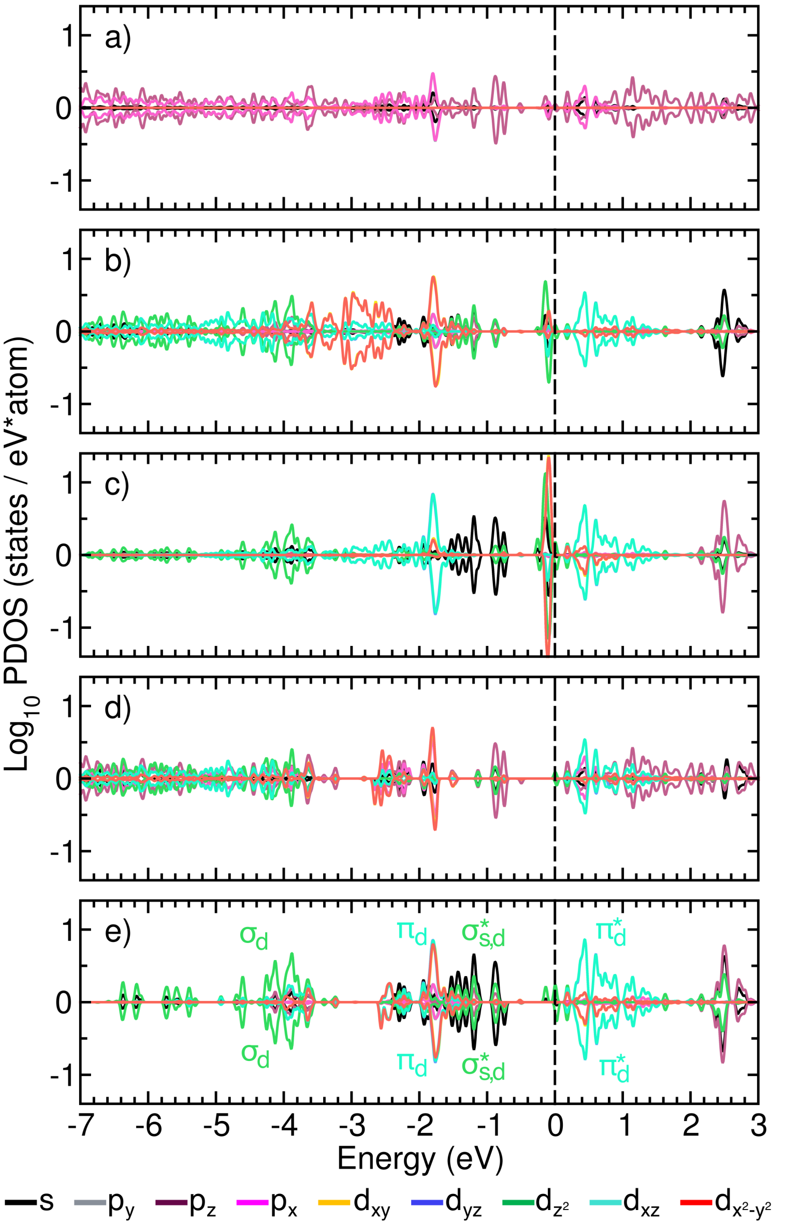


**Figure S7: Bond-order analysis for IrPt@NSV based on eigenstate-reconstructed PDOS using POET.** Reconstructed atomic PDOS for **(a)** the three N atoms forming the NSV defect ($N_3$), **(b)** Pt, **(c)** Ir. **(d,e)** Molecular orbitals reconstructed from hybridized states between **(d)** $N_3$ and Pt, and **(e)** Pt and Ir.

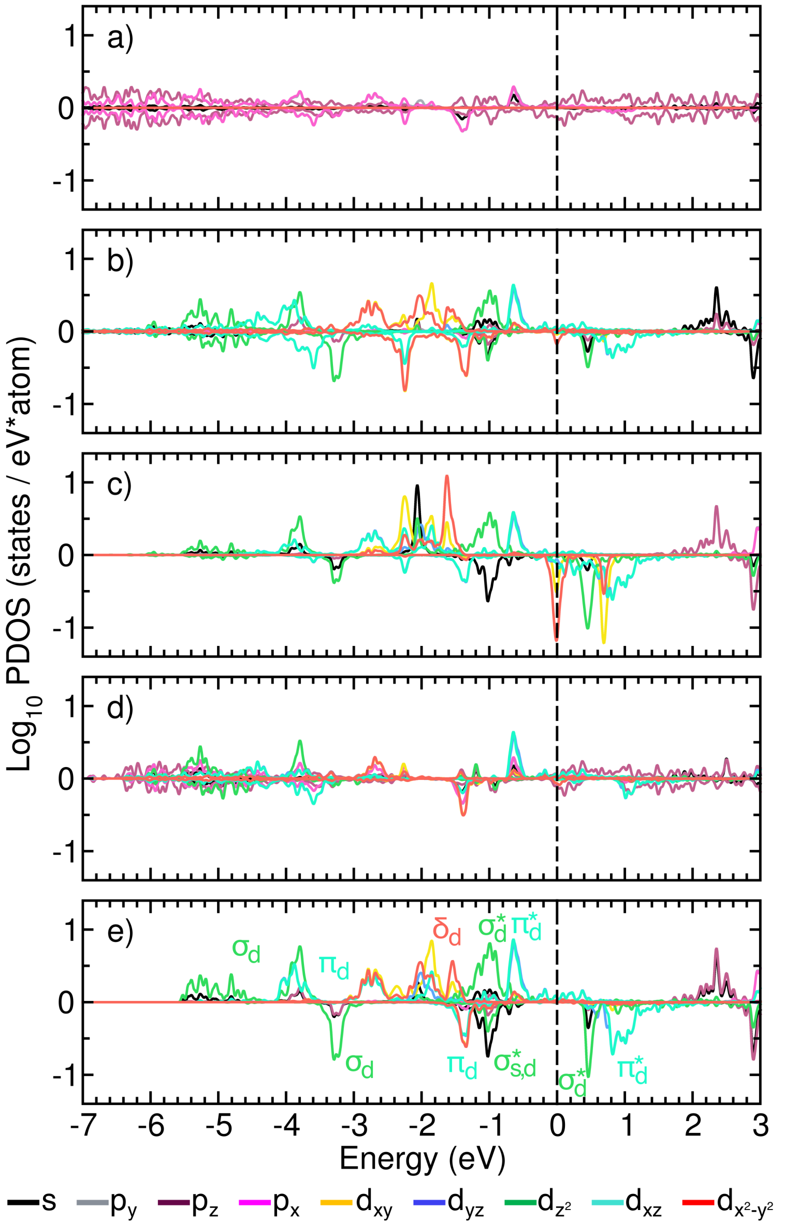


**Figure S8: Bond-order analysis for OsPd@NSV@Cu(111) based on eigenstate-reconstructed PDOS using POET.** Reconstructed atomic PDOS for **(a)** the three N atoms forming the NSV defect ($N_3$), **(b)** Pd, **(c)** Os. **(d,e)** Molecular orbitals reconstructed from hybridized states between **(d)** $N_3$ and Pd, and **(e)** Pd and Os.

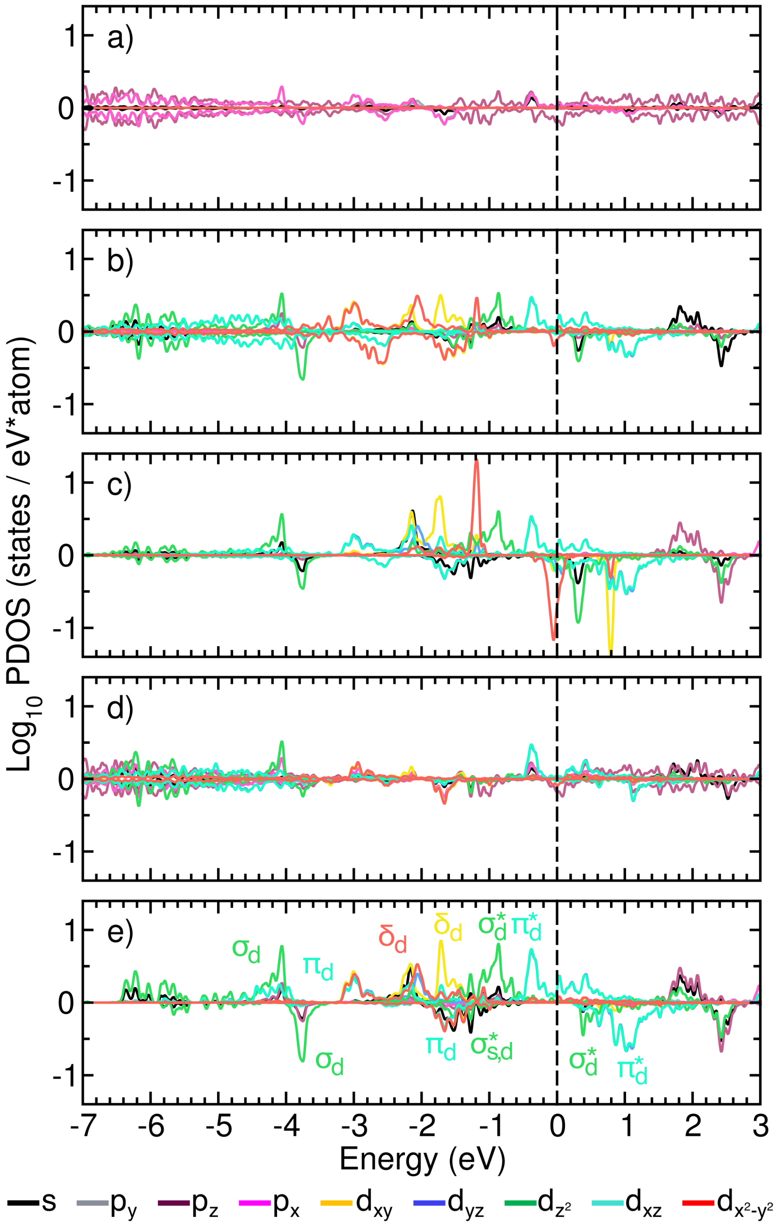


**Figure S9: Bond-order analysis for OsPt@NSV@Cu(111) based on eigenstate-reconstructed PDOS using POET.** Reconstructed atomic PDOS for **(a)** the three N atoms forming the NSV defect ($N_3$), **(b)** Pt, **(c)** Os. **(d,e)** Molecular orbitals reconstructed from hybridized states between **(d)** $N_3$ and Pt, and **(e)** Pt and Os.

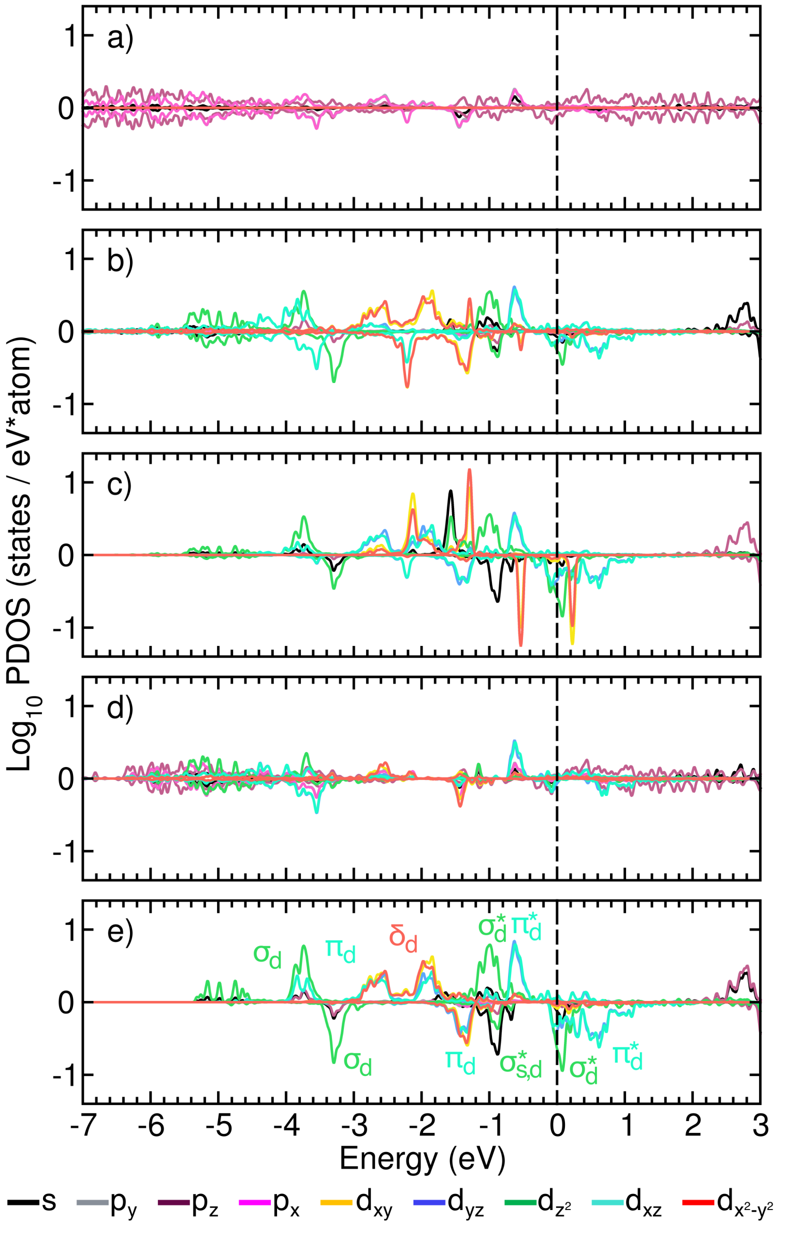


**Figure S10: Bond-order analysis for IrPd@NSV@Cu(111) based on eigenstate-reconstructed PDOS using POET.** Reconstructed atomic PDOS for **(a)** the three N atoms forming the NSV defect ($N_3$), **(b)** Pd, **(c)** Ir. **(d,e)** Molecular orbitals reconstructed from hybridized states between **(d)** $N_3$ and Pd, and **(e)** Pd and Ir.

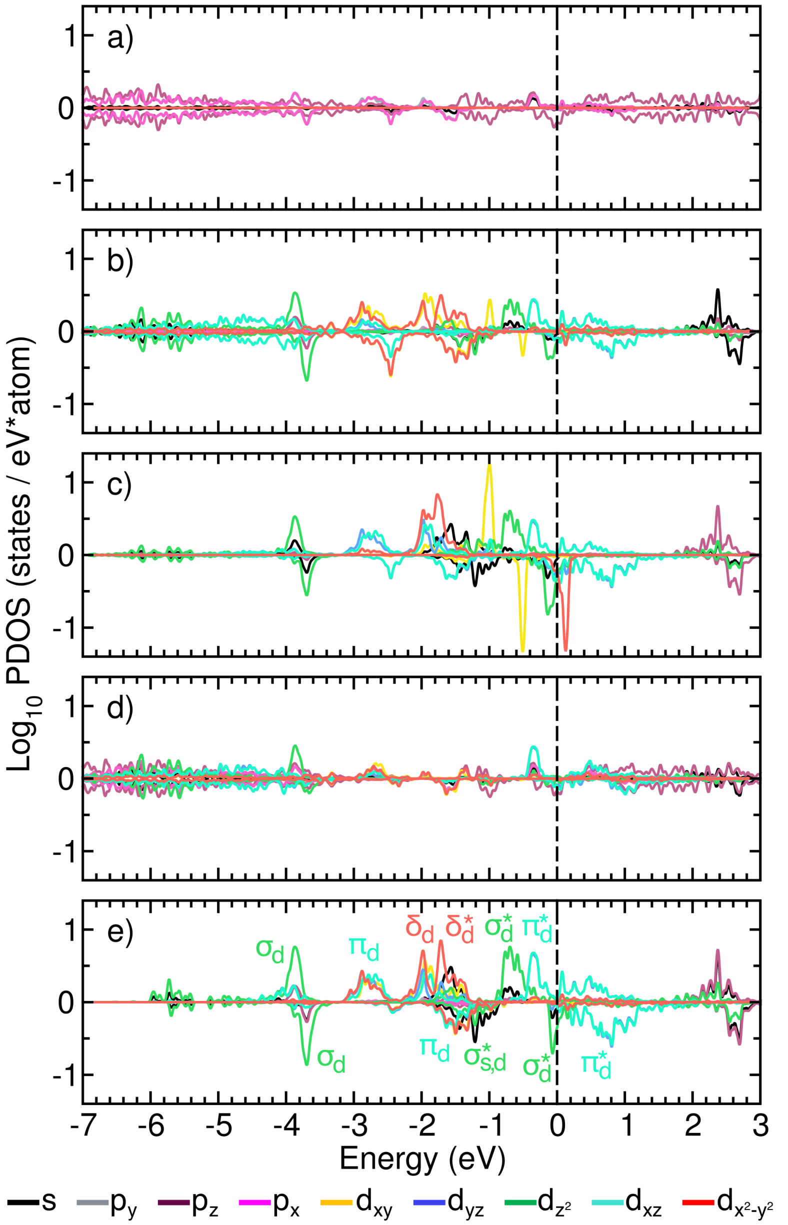


**Figure S11: Bond-order analysis for IrPt@NSV@Cu(111) based on eigenstate-reconstructed PDOS using POET.** Reconstructed atomic PDOS for **(a)** the three N atoms forming the NSV defect ($N_3$), **(b)** Pt, **(c)** Ir. **(d,e)** Molecular orbitals reconstructed from hybridized states between **(d)** $N_3$ and Pt, and **(e)** Pt and Ir.

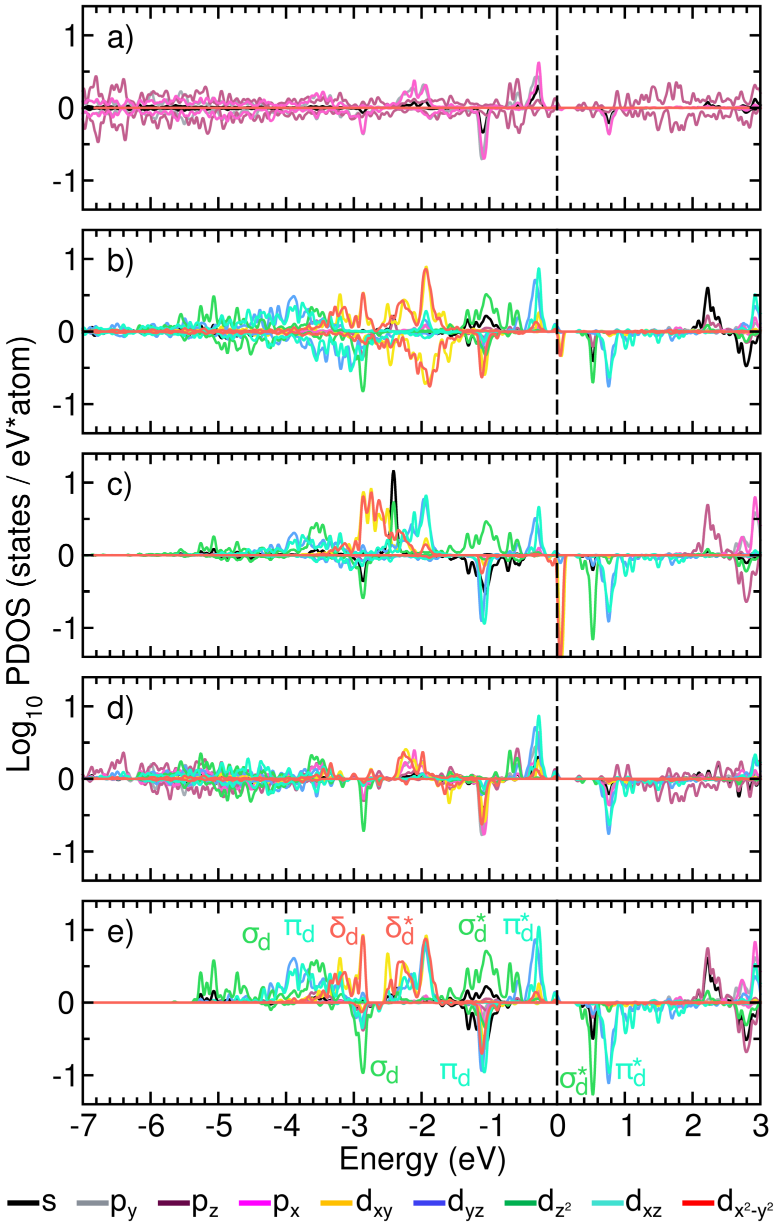


**Figure S12: Bond-order analysis for OsPd@NSV@MgO(001) based on eigenstate-reconstructed PDOS using POET.** Reconstructed atomic PDOS for **(a)** the three N atoms forming the NSV defect ($N_3$), **(b)** Pd, **(c)** Os. **(d,e)** Molecular orbitals reconstructed from hybridized states between **(d)** $N_3$ and Pd, and **(e)** Pd and Os.

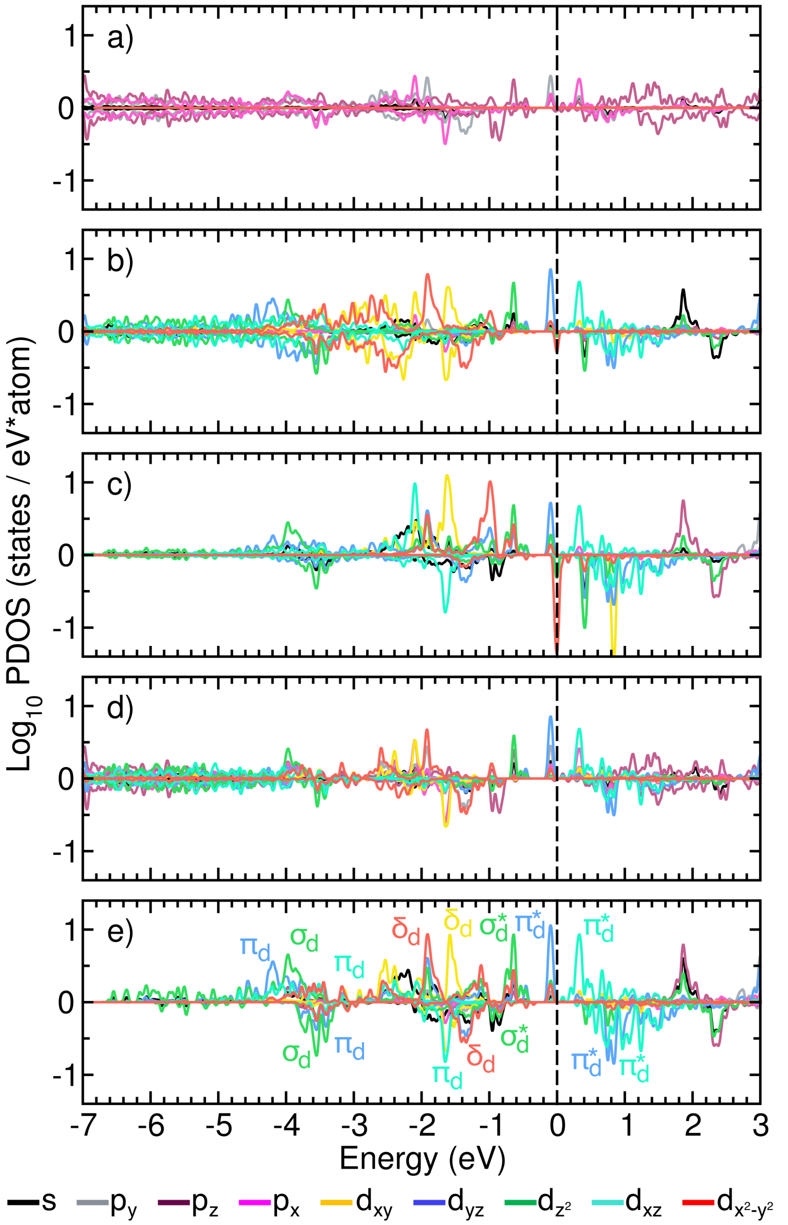


**Figure S13: Bond-order analysis for OsPt@NSV@MgO(001) based on eigenstate-reconstructed PDOS using POET.** Reconstructed atomic PDOS for **(a)** the three N atoms forming the NSV defect ($N_3$), **(b)** Pt, **(c)** Os. **(d,e)** Molecular orbitals reconstructed from hybridized states between **(d)** $N_3$ and Pt, and **(e)** Pt and Os.

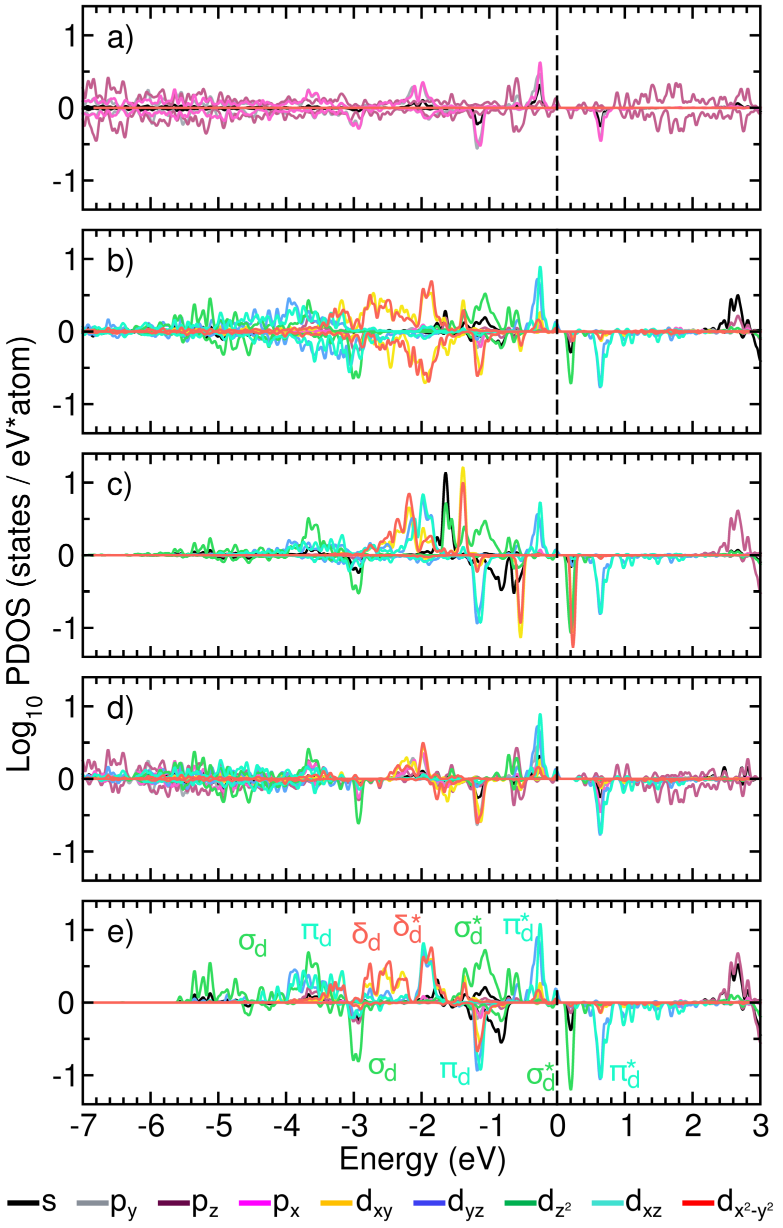


**Figure S14: Bond-order analysis for IrPd@NSV@MgO(001) based on eigenstate-reconstructed PDOS using POET.** Reconstructed atomic PDOS for **(a)** the three N atoms forming the NSV defect ($N_3$), **(b)** Pd, **(c)** Ir. **(d,e)** Molecular orbitals reconstructed from hybridized states between **(d)** $N_3$ and Pd, and **(e)** Pd and Ir.

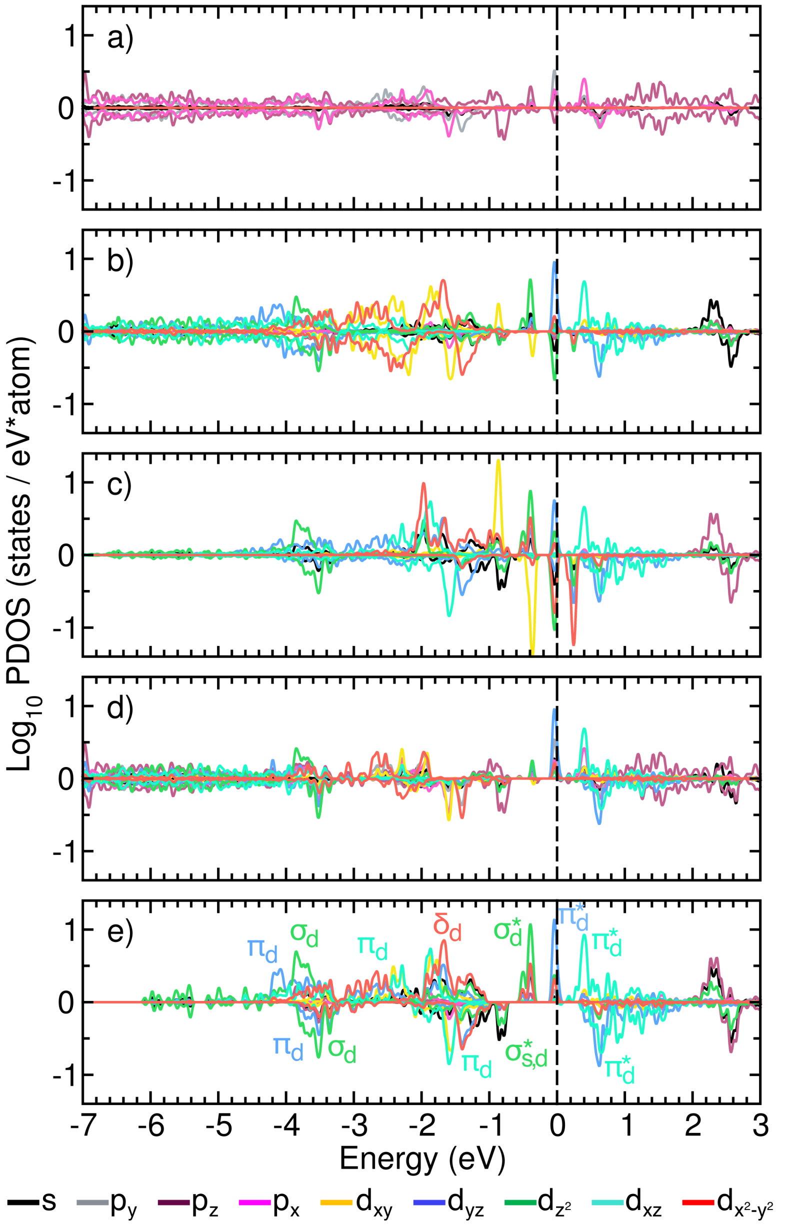


**Figure S15: Bond-order analysis for IrPt@NSV@MgO(001) based on eigenstate-reconstructed PDOS using POET.** Reconstructed atomic PDOS for **(a)** the three N atoms forming the NSV defect ($N_3$), **(b)** Pt, **(c)** Ir. **(d,e)** Molecular orbitals reconstructed from hybridized states between **(d)** $N_3$ and Pt, and **(e)** Pt and Ir.

# S3 Band-structure reconstruction for FT-MAE analysis in POET

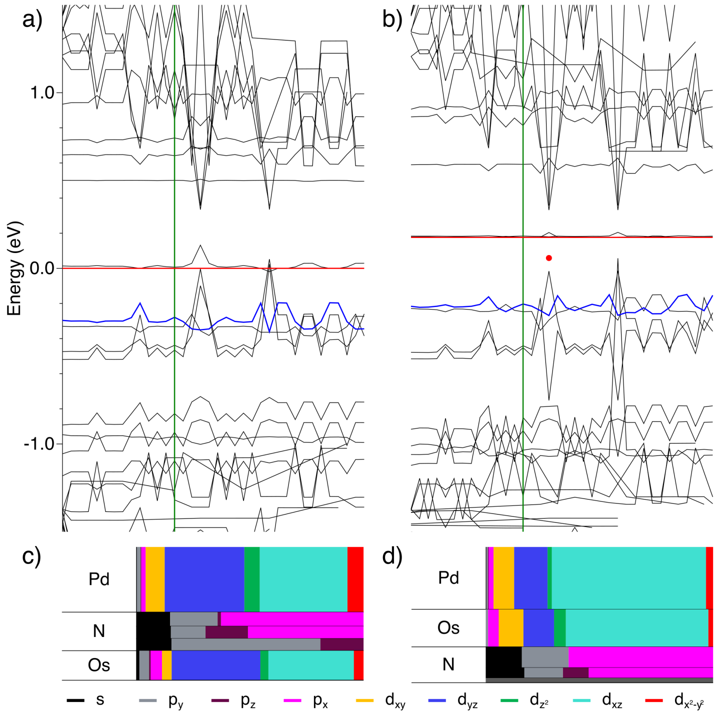


**Figure S16: Interactive band-structure visualization for magnetic-force-theorem analysis of MAE within the POET web application (reconstructed for freestanding OsPd@NSV).** While individual data points represent discrete eigenvalues extracted from the VASP PROCAR file, their connectivity is established by the POET console application. Band structures are shown for magnetizations oriented **(a)** parallel and **(b)** perpendicular to the Os–Pd bond axis. The green vertical line indicates the selected **k** point, while the chosen band is highlighted in blue; both selections are interactive and mouse-driven. The two panels are synchronized to maintain consistent state tracking across different magnetization directions. Users can also navigate through bands and *k*-points using the keyboard arrow keys (Up/Down and Left/Right, respectively). **(c, d)** Relative site- and orbital-resolved compositions of the selected state, corresponding to the active selections in panels **(a)** and **(b)**, respectively. If the automated connectivity appears physically unreasonable, the console application parameters can be adjusted and rerun; alternatively, switching the rendering mode allows users to display discrete eigenvalue spectra similar to those shown in Fig. S32b.

# S4 Halogenation

**Table S4: Scalar-relativistic properties of halogenated pristine graphene (G) and NSV–graphene.** The adsorption energy referenced to an isolated halogen atom $E_{aa\mathrm{X}}$ and a diatomic molecule $X_2$, $E_{am\mathrm{X}}$ (in eV). The vertical distance above the sheet $d_{v\mathrm{Xg}}$ and the bond length between the halogen atom and its nearest neighbor $d_{\mathrm{X-NN}}$ (in Å).

| System | Adsorption site | $E_{aa\mathrm{X}}$ | $E_{am\mathrm{X}}$ | $d_{v\mathrm{Xg}}$ | $d_{\mathrm{X-NN}}$ |
|---|---|---|---|---|---|
| $F_1G$ | T | −1.86 | −0.69 | 1.98 | 1.57 |
| $Cl_1G$ | T | −1.12 | 0.43 | 2.97 | 2.97 |
| $Cl_1G$ | B | −1.11 | 0.44 | 3.10 | 3.20 |
| $Br_1G$ | T | −0.87 | 0.43 | 3.19 | 3.23 |
| $I_1G$ | T | −0.66 | 0.51 | 3.46 | 3.48 |
| $I_1G$ | B | −0.66 | 0.51 | 3.44 | 3.53 |
| $I_1G$ | H | −0.64 | 0.53 | 3.54 | 3.76 |
| $F_1NSV$ | T | −2.70 | −1.53 | 2.02 | 1.48 |
| $F_1NSV$ | N | −1.88 | −0.70 | 1.62 | 1.47 |
| $Cl_1NSV$ | T | −1.51 | 0.04 | 2.47 | 1.94 |
| $Cl_1NSV$ | N | −1.34 | 0.21 | 2.03 | 1.74 |
| $Br_1NSV$ | T | −1.08 | 0.21 | 2.67 | 2.23 |
| $Br_1NSV$ | N | −0.99 | 0.31 | 2.25 | 2.01 |
| $I_1NSV$ | N | −0.90 | 0.27 | 2.40 | 2.19 |
| $I_1NSV$ | T | −0.76 | 0.41 | 2.98 | 2.65 |

**Table S5: Scalar-relativistic properties of halogenated TM@NSV.** The adsorption energy referenced to an isolated halogen atom $\overline{E_{aaX}}$ and a diatomic molecule $X_2$, $\overline{E_{amX}}$ (in eV). The average TM–N bond length $d_{bl}$ and the TM–X distance (in Å). The total magnetic moment of the supercell $\mu_{tot}$ and the local magnetic moment on the TM atom $\mu_{TM}$ (in $\mu_B$). The Bader charge of the TM atom $q_{TM}$ and the average Bader charge of the halogen atoms $\overline{q_X}$ (in e). See Figure S18 for the multi-halogen adsorption configurations.

| System | $\overline{E_{aaX}}$ | $\overline{E_{amX}}$ | $\overline{d_{bl}}$ | $\overline{d_{TM-X}}$ | $\mu_{tot}$ | $\mu_{TM}$ | $q_{TM}$ | $\overline{q_X}$ |
|---|---|---|---|---|---|---|---|---|
| Co@NSV | | | 1.83 | | 2.11 | 1.87 | 0.78 | |
| $F_1$Co@NSV | −5.30 | −4.13 | 1.92 | 1.79 | 3.00 | 2.38 | 1.18 | −0.69 |
| $Cl_1$Co@NSV | −4.34 | −2.79 | 1.91 | 2.14 | 3.00 | 2.31 | 1.00 | −0.56 |
| $Br_1$Co@NSV | −3.83 | −2.54 | 1.91 | 2.29 | 3.00 | 2.30 | 0.98 | −0.55 |
| $I_1$Co@NSV | −3.36 | −2.19 | 1.90 | 2.46 | 3.00 | 2.26 | 0.88 | −0.46 |
| $F_2$Co@NSV | −4.63 | −3.46 | 1.85 | 1.81 | 0.00 | 0.00 | 1.25 | −0.61 |
| $Cl_2$Co@NSV | −3.55 | −2.00 | 1.89 | 2.19 | 0.00 | 0.00 | 1.04 | −0.50 |
| $Br_2$Co@NSV | −2.98 | −1.69 | 1.92 | 2.37 | 2.00 | 1.44 | 0.97 | −0.46 |
| $I_2$Co@NSV | −2.59 | −1.42 | 1.89 | 2.56 | 0.00 | 0.00 | 0.84 | −0.35 |
| $F_3$Co@NSV | −4.25 | −3.08 | 1.87 | 1.82 | 1.00 | 0.47 | 1.40 | −0.58 |
| $Cl_3$Co@NSV | −3.09 | −1.55 | 1.92 | 2.24 | 1.00 | 0.39 | 1.08 | −0.45 |
| $Br_3$Co@NSV | −2.61 | −1.32 | 1.93 | 2.42 | 1.00 | 0.39 | 0.96 | −0.38 |
| $I_3$Co@NSV | −2.19 | −1.02 | 1.92 | 2.65 | 0.94 | 0.08 | 0.81 | −0.25 |
| Ir@NSV | | | 2.01 | | 1.89 | 0.75 | 0.59 | |
| $F_1$Ir@NSV | −5.71 | −4.54 | 2.02 | 1.93 | 0.82 | 0.28 | 0.98 | −0.59 |
| $Cl_1$Ir@NSV | −5.09 | −3.54 | 2.03 | 2.21 | 0.82 | 0.26 | 0.83 | −0.44 |
| $Br_1$Ir@NSV | −4.64 | −3.34 | 2.03 | 2.36 | 0.81 | 0.26 | 0.74 | −0.35 |
| $I_1$Ir@NSV | −4.28 | −3.11 | 2.03 | 2.51 | 0.80 | 0.25 | 0.65 | −0.25 |
| $F_2$Ir@NSV | −5.42 | −4.25 | 1.98 | 1.97 | 0.00 | 0.00 | 1.34 | −0.62 |
| $Cl_2$Ir@NSV | −4.58 | −3.03 | 2.00 | 2.31 | 0.00 | 0.00 | 1.05 | −0.48 |
| $Br_2$Ir@NSV | −4.12 | −2.82 | 2.01 | 2.46 | 0.00 | 0.00 | 0.90 | −0.41 |
| $I_2$Ir@NSV | −3.71 | −2.54 | 2.02 | 2.63 | 0.00 | 0.00 | 0.72 | −0.30 |
| $F_3$Ir@NSV | −5.07 | −3.90 | 1.96 | 1.97 | 1.00 | 0.47 | 1.68 | −0.59 |
| $Cl_3$Ir@NSV | −4.10 | −2.55 | 2.01 | 2.34 | 1.00 | 0.39 | 1.20 | −0.45 |
| $Br_3$Ir@NSV | −3.62 | −2.32 | 2.03 | 2.49 | 1.00 | 0.37 | 1.00 | −0.38 |
| $I_3$Ir@NSV | −3.18 | −2.02 | 2.05 | 2.68 | 1.00 | 0.34 | 0.72 | −0.26 |

| System | $\overline{E_{aaX}}$ | $\overline{E_{amX}}$ | $\overline{d_{bl}}$ | $\overline{d_{TM-X}}$ | $\mu_{tot}$ | $\mu_{TM}$ | $q_{TM}$ | $\overline{q_X}$ |
|---|---|---|---|---|---|---|---|---|
| Mn@NSV | | | 1.79 | | 0.45 | 0.52 | 0.96 | |
| $F_1$Mn@NSV | −6.03 | −4.86 | 1.83 | 1.79 | 1.00 | 1.13 | 1.31 | −0.64 |
| $Cl_1$Mn@NSV | −4.89 | −3.34 | 1.83 | 2.16 | 1.00 | 1.14 | 1.19 | −0.56 |
| $Br_1$Mn@NSV | −4.35 | −3.06 | 1.83 | 2.31 | 1.00 | 1.16 | 1.15 | −0.53 |
| $I_1$Mn@NSV | −3.82 | −2.65 | 1.83 | 2.50 | 1.00 | 1.16 | 1.09 | −0.47 |
| $F_2$Mn@NSV | −5.63 | −4.46 | 2.03 | 1.81 | 4.00 | 3.44 | 1.54 | −0.63 |
| $Cl_2$Mn@NSV | −4.36 | −2.81 | 2.04 | 2.21 | 4.00 | 3.47 | 1.38 | −0.55 |
| $Br_2$Mn@NSV | −3.81 | −2.52 | 2.04 | 2.38 | 4.00 | 3.51 | 1.31 | −0.50 |
| $I_2$Mn@NSV | −3.29 | −2.12 | 2.04 | 2.59 | 4.00 | 3.54 | 1.22 | −0.43 |
| $F_3$Mn@NSV | −5.11 | −3.94 | 1.99 | 1.80 | 3.00 | 2.64 | 1.76 | −0.57 |
| $Cl_3$Mn@NSV | −3.71 | −2.16 | 2.02 | 2.22 | 3.00 | 2.67 | 1.42 | −0.46 |
| $Br_3$Mn@NSV | −3.16 | −1.86 | 2.04 | 2.40 | 3.00 | 2.75 | 1.30 | −0.41 |
| $I_3$Mn@NSV | −2.64 | −1.47 | 2.04 | 2.64 | 3.00 | 2.90 | 1.14 | −0.33 |
| Pd@NSV | | | 2.17 | | 1.00 | 0.54 | 0.48 | |
| $F_1$Pd@NSV | −4.47 | −3.30 | 2.11 | 1.94 | 0.00 | 0.00 | 0.84 | −0.63 |
| $Cl_1$Pd@NSV | −3.82 | −2.27 | 2.12 | 2.27 | 2.00 | 0.97 | 0.69 | −0.51 |
| $Br_1$Pd@NSV | −3.43 | −2.13 | 2.13 | 2.40 | 2.00 | 0.96 | 0.62 | −0.44 |
| $I_1$Pd@NSV | −3.12 | −1.95 | 2.13 | 2.54 | 2.00 | 0.92 | 0.51 | −0.31 |
| $F_2$Pd@NSV | −4.29 | −3.12 | 2.05 | 1.95 | 1.00 | 0.48 | 1.14 | −0.60 |
| $Cl_2$Pd@NSV | −3.51 | −1.97 | 2.08 | 2.29 | 1.00 | 0.45 | 0.86 | −0.45 |
| $Br_2$Pd@NSV | −3.10 | −1.81 | 2.10 | 2.43 | 1.00 | 0.45 | 0.72 | −0.37 |
| $I_2$Pd@NSV | −2.76 | −1.59 | 2.11 | 2.60 | 1.00 | 0.43 | 0.55 | −0.26 |
| $F_3$Pd@NSV | −4.07 | −2.90 | 1.98 | 1.94 | 0.00 | 0.00 | 1.39 | −0.56 |
| $Cl_3$Pd@NSV | −3.17 | −1.62 | 2.05 | 2.32 | 0.00 | 0.00 | 1.01 | −0.39 |
| $Br_3$Pd@NSV | −2.76 | −1.47 | 2.08 | 2.47 | 0.00 | 0.00 | 0.83 | −0.31 |
| $I_3$Pd@NSV | −2.44 | −1.27 | 2.11 | 2.65 | 0.00 | 0.00 | 0.57 | −0.18 |
| Pt@NSV | | | 2.15 | | 1.00 | 0.53 | 0.42 | |
| $F_1$Pt@NSV | −5.14 | −3.97 | 2.12 | 1.96 | 2.00 | 0.98 | 0.78 | −0.60 |
| $Cl_1$Pt@NSV | −4.67 | −3.12 | 2.15 | 2.22 | 2.00 | 0.96 | 0.66 | −0.45 |
| $Br_1$Pt@NSV | −4.27 | −2.98 | 2.15 | 2.36 | 2.00 | 0.95 | 0.51 | −0.29 |
| $I_1$Pt@NSV | −3.99 | −2.82 | 2.15 | 2.50 | 2.00 | 0.91 | 0.37 | −0.15 |
| $F_2$Pt@NSV | −4.91 | −3.74 | 2.06 | 1.96 | 1.00 | 0.49 | 1.16 | −0.59 |
| $Cl_2$Pt@NSV | −4.16 | −2.62 | 2.09 | 2.29 | 1.00 | 0.46 | 0.87 | −0.44 |
| $Br_2$Pt@NSV | −3.73 | −2.44 | 2.10 | 2.44 | 1.00 | 0.47 | 0.70 | −0.35 |
| $I_2$Pt@NSV | −3.38 | −2.21 | 2.11 | 2.60 | 1.00 | 0.45 | 0.50 | −0.23 |
| $F_3$Pt@NSV | −4.69 | −3.52 | 1.98 | 1.97 | 0.00 | 0.00 | 1.51 | −0.57 |
| $Cl_3$Pt@NSV | −3.80 | −2.25 | 2.04 | 2.33 | 0.00 | 0.00 | 1.10 | −0.41 |
| $Br_3$Pt@NSV | −3.35 | −2.06 | 2.07 | 2.48 | 0.00 | 0.00 | 0.86 | −0.32 |
| $I_3$Pt@NSV | −2.99 | −1.82 | 2.09 | 2.66 | 0.00 | 0.00 | 0.58 | −0.20 |

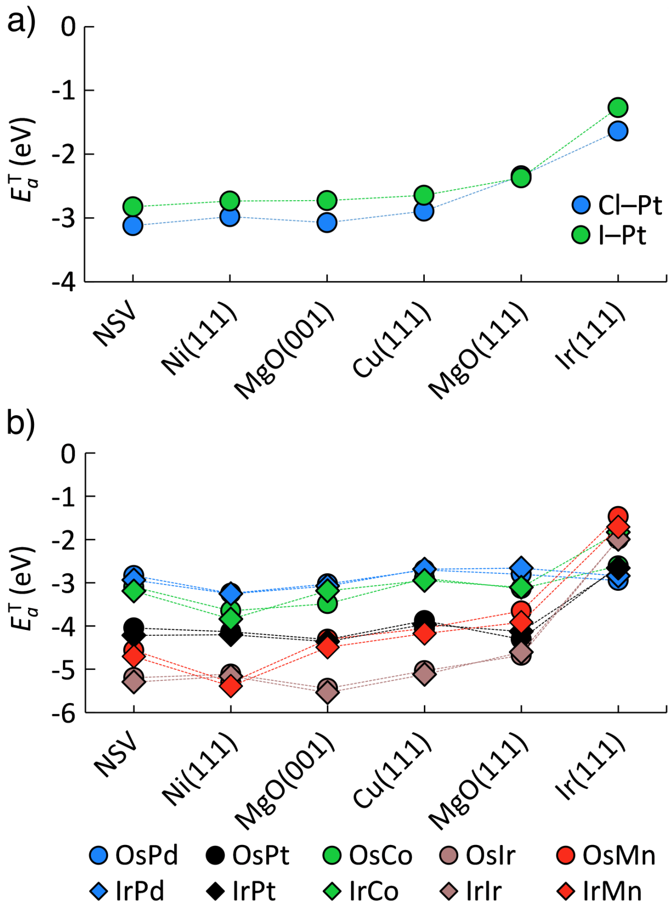


**Figure S17: Adsorption energies ($E_a^T$) (in eV) on freestanding and substrate-supported NSV-graphene. (a)** Halogen atoms on Pt@NSV. **(b)** Upper transition metal atoms in upright dimers.

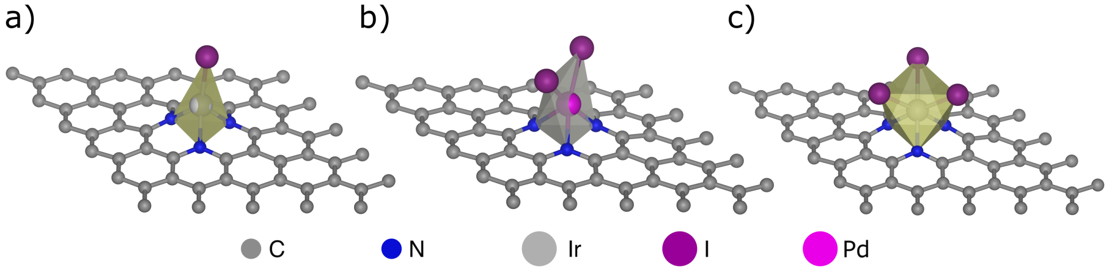


**Figure S18: Structures of halogenated freestanding TM@NSV systems.** Polyhedra highlight the coordination environment of the TM atom. **(a)** I–IrNSV, **(b)** $I_2$–PdNSV, and **(c)** $I_3$–IrNSV. Plotted using VESTA [5].

**Table S6: Scalar-relativistic properties of halogenated Pt@NSV, both freestanding and supported.** The adsorption energy referenced to an isolated halogen atom (Cl, I) $E_{aa\mathrm{X}}$ and a diatomic molecule $E_{am\mathrm{X}}$ (in eV). The average TM–N bond length $d_{bl}$ and the TM–X distance (in Å). The local magnetic moment on the TM atom $\mu_{\mathrm{TM}}$ (in $\mu_{\mathrm{B}}$). The Bader charge of the Pt atom before $q_{b\mathrm{TM}}$ and after halogen adsorption $q_{\mathrm{TM}}$, and the Bader charge of the halogen atom $q_{\mathrm{X}}$ (in e). See Figure S1 for the notation.

| System | $E_{aa\mathrm{X}}$ | $E_{am\mathrm{X}}$ | $\overline{d_{bl}}$ | $d_{\mathrm{TM-X}}$ | $\mu_{\mathrm{TM}}$ | $q_{b\mathrm{TM}}$ | $q_{\mathrm{TM}}$ | $q_{\mathrm{X}}$ |
|---|---|---|---|---|---|---|---|---|
| Cl–Pt@NSV | −4.67 | −3.12 | 2.15 | 2.22 | 0.96 | 0.42 | 0.66 | −0.45 |
| Cl–Pt@NSV@Cu(111) | −4.44 | −2.89 | 2.19 | 2.23 | 0.00 | 0.41 | 0.56 | −0.44 |
| Cl–Pt@NSV@Ni(111) | −4.52 | −2.98 | 2.20 | 2.22 | 0.32 | 0.28 | 0.39 | −0.35 |
| Cl–Pt@NSV@Ir(111) | −3.18 | −1.63 | 2.23 | 2.28 | 0.00 | 0.64 | 0.58 | −0.48 |
| Cl–Pt@NSV@MgO(001) | −4.62 | −3.07 | 2.19 | 2.21 | 0.00 | 0.36 | 0.58 | −0.41 |
| I–Pt@NSV | −3.99 | −2.82 | 2.15 | 2.50 | 0.91 | 0.42 | 0.37 | −0.15 |
| I–Pt@NSV@Cu(111) | −3.81 | −2.64 | 2.19 | 2.50 | 0.00 | 0.41 | 0.27 | −0.16 |
| I–Pt@NSV@Ni(111) | −3.90 | −2.73 | 2.20 | 2.49 | 0.30 | 0.28 | 0.21 | −0.16 |
| I–Pt@NSV@Ir(111) | −2.44 | −1.27 | 2.25 | 2.56 | 0.00 | 0.64 | 0.39 | −0.27 |
| I–Pt@NSV@MgO(111)$_{\mathrm{smE}}$ | −3.54 | −2.37 | 2.07 | 2.47 | 0.47 | 0.68 | 0.54 | 0.07 |
| I–Pt@NSV@MgO(111)$_{\mathrm{lmE}}$ | −3.35 | −2.19 | 2.08 | 2.48 | 0.48 | 0.70 | 0.55 | 0.04 |
| I–Pt@NSV@MgO(001) | −3.89 | −2.73 | 2.19 | 2.49 | 0.00 | 0.36 | 0.38 | −0.19 |

## S5 TM adatoms

TM adsorption on NSV-graphene follows a donor–acceptor mechanism, where nitrogen lone pairs donate into vacant TM *d* orbitals to form coordinate covalent bonds. Despite this donation, Bader charge analysis [6, 7] assigns net positive charges to all TMs (Table S7), reflecting electron depletion driven by electronegativity differences and metal-to-ligand back-donation. The magnitude of charge transfer generally correlates with TM electronegativity, with less electronegative elements showing stronger depletion. Departures from this trend, such as Ir being more positively charged than Co on Ni(111), or Pt exceeding Pd on Ir(111), arise from substrate-mediated charge redistribution.

On Ni(111), strong substrate-to-NSV charge transfer reduces electron withdrawal from the TM, mitigating the ionic character of the TM–N bond and making adsorption less exothermic by up to 3.17 eV. Conversely, for most NSV-graphene configurations on MgO(111), electron flow from NSV-graphene toward the substrate enhances TM-to-NSV donation and induces a local reconstruction that strengthens both NSV–substrate and TM–C/N interactions (except when N–O bonds form, where N sites suppress this electron flow relative to C sites). On Ir(111), additional metal–metal interactions between the TM and the substrate (Figure S19) stabilizes adsorption, particularly for Pt, Ir, and Mn. Co gains less due to suppression of its high-spin state, whereas closed-shell Pd, lacking metal–metal bonding, exhibits negligible change in adsorption energy. On Cu(111) and MgO(001), adsorption energies remain largely unaffected ($< 0.3$ eV), except for Mn, where high-spin stabilization dominates. For example, Mn@NSV@Cu(111) adopts a 4.41 $\mu_B$ ground-state (GS), 1.32 eV below the nonmagnetic configuration, whereas freestanding Mn@NSV favors a low-spin (0.51 $\mu_B$) state, with the high-spin variant lying 0.23 eV higher.

The magnetic response of TM@NSV systems thus reflects a competition between covalent bonding and spin retention. TM adsorption on freestanding NSV-graphene typically lowers the TM moment by $\sim$1 $\mu_B$ [8], as unpaired electrons occupy spin-down bonding orbitals between TM and NSV atoms, consistent with donor-acceptor bonding. Mn shows the strongest reduction, from 5.00 $\mu_B$ in the gas phase to 0.51 $\mu_B$ on NSV, highlighting its distinct electronic response to coordination. Substrate binding further modifies TM spin states, often quenching magnetic moments through hybridization with low-energy substrate states that promote electron pairing in TM–NSV–substrate molecular orbitals. While Co generally resists quenching, retaining 2 $\mu_B$ on most substrates, its moment collapses on Ir(111) and most MgO(111) configurations. In contrast, Mn exhibits the opposite behavior, with its moment increasing from 0.5 $\mu_B$ in freestanding NSV-graphene to $\sim$4 $\mu_B$ when supported, consistent with our earlier findings [8] that late-period TMs maximize covalent bonding, while earlier-period elements retain magnetic moments by forming fewer bonds.

**Table S7: Scalar-relativistic characteristics of TM adatoms on freestanding and supported NSV–graphene.** The adsorption energy $E_a$ (in eV). The average bond length between the TM atom and the coordinating N atoms $d_{bl}$, the average vertical distance (along the surface normal) between the TM atom and the N atoms $d_{van}$, the average vertical distance between the TM atom and all atoms forming the NSV–graphene $d_{vag}$, and the average vertical distance between the TM atom and atoms in the topmost layer of the substrate $d_{vas}$(all in Å). The total magnetic moment relative to the corresponding substrate-only system $\mu_{\mathrm{red}}$ and the local magnetic moment on the TM atom $\mu_{\mathrm{TM}}$ (both in $\mu_{\mathrm{B}}$). The Bader charge of the TM atom $q_{\mathrm{TM}}$ (in e). See Figure S1 for the notation.

| Substrate | $E_a$ | $d_{bl}$ | $d_{van}$ | $d_{vag}$ | $d_{vas}$ | $d_{vns}$ | $\mu_{\mathrm{red}}$ | $\mu_{\mathrm{TM}}$ | $q_{\mathrm{TM}}$ |
|---|---|---|---|---|---|---|---|---|---|
| | | | | Pd | | | | | |
| NSV | −2.38 | 2.17 | 1.54 | 1.72 | | | 1.00 | 0.54 | 0.48 |
| Cu(111) | −2.45 | 2.20 | 1.46 | 1.18 | 3.86 | 2.40 | 0.00 | 0.00 | 0.42 |
| Ni(111) | −1.69 | 2.60 | 2.10 | 1.76 | 4.19 | 2.09 | 1.44 | 0.01 | 0.21 |
| Ir(111) | −2.44 | 2.21 | 1.29 | 1.37 | 3.42 | 2.13 | 0.37 | 0.31 | 0.39 |
| $MgO(111)_{\mathrm{smH}}$ | −2.58 | 2.55 | 1.65 | 1.22 | 3.41 | 1.76 | −3.50 | 0.01 | 0.37 |
| $MgO(111)_{\mathrm{smT}}$ | −3.82 | 2.06 | 1.18 | 1.17 | 3.42 | 2.24 | 0.50 | 0.19 | 0.65 |
| $MgO(111)_{\mathrm{smE}}$ | −3.66 | 2.06 | 1.34 | 2.14 | 4.38 | 3.05 | 2.26 | 0.96 | 0.72 |
| $MgO(111)_{\mathrm{lmH}}$ | −2.57 | 2.10 | 1.13 | 0.44 | 2.56 | 1.43 | 1.18 | 0.05 | 0.52 |
| $MgO(111)_{\mathrm{lmT}}$ | −6.43 | 1.94 | 0.90 | 0.83 | 2.87 | 1.97 | −1.39 | −0.07 | 0.77 |
| $MgO(111)_{\mathrm{lmE}}$ | −3.80 | 2.05 | 1.20 | 1.73 | 3.81 | 2.61 | 2.87 | 0.96 | 0.72 |
| MgO(001) | −2.63 | 2.18 | 1.55 | 1.69 | 4.91 | 3.36 | 1.00 | 0.52 | 0.47 |
| | | | | Pt | | | | | |
| NSV | −2.87 | 2.15 | 1.51 | 1.71 | | | 1.00 | 0.53 | 0.42 |
| Cu(111) | −2.87 | 2.15 | 1.41 | 1.14 | 3.83 | 2.42 | 0.01 | 0.01 | 0.41 |
| Ni(111) | −2.40 | 2.18 | 1.55 | 1.25 | 3.67 | 2.12 | 1.50 | 0.07 | 0.28 |
| Ir(111) | −4.26 | 1.99 | −0.07 | 0.45 | 2.54 | 2.61 | −0.02 | 0.00 | 0.64 |
| $MgO(111)_{\mathrm{smH}}$ | −4.56 | 2.07 | 0.97 | 0.61 | 2.79 | 1.82 | −5.50 | −0.03 | 0.57 |
| $MgO(111)_{\mathrm{smT}}$ | −4.97 | 2.03 | 1.12 | 1.06 | 3.31 | 2.19 | 0.27 | 0.18 | 0.67 |
| $MgO(111)_{\mathrm{smE}}$ | −4.40 | 2.04 | 1.28 | 2.12 | 4.37 | 3.09 | 2.36 | 1.05 | 0.68 |
| $MgO(111)_{\mathrm{lmH}}$ | −3.35 | 2.04 | 1.05 | 0.35 | 2.48 | 1.43 | 1.23 | 0.05 | 0.54 |
| $MgO(111)_{\mathrm{lmT}}$ | −7.90 | 1.92 | 0.85 | 0.78 | 2.82 | 1.97 | −0.76 | −0.04 | 0.81 |
| $MgO(111)_{\mathrm{lmE}}$ | −4.60 | 2.03 | 1.13 | 1.69 | 3.78 | 2.65 | 2.50 | 1.00 | 0.70 |
| MgO(001) | −3.14 | 2.19 | 1.58 | 1.75 | 4.97 | 3.40 | 1.00 | 0.52 | 0.36 |

| Substrate | $E_a$ | $d_{bl}$ | $d_{van}$ | $d_{vag}$ | $d_{vas}$ | $d_{vns}$ | $\mu_{\text{red}}$ | $\mu_{\text{TM}}$ | $q_{\text{TM}}$ |
|---|---|---|---|---|---|---|---|---|---|
| | | | | Co | | | | | |
| NSV | −4.88 | 1.83 | 0.87 | 1.34 | | | 2.16 | 1.88 | 0.79 |
| Cu(111) | −5.01 | 1.81 | 0.60 | 0.79 | 3.81 | 3.21 | 2.39 | 1.91 | 0.84 |
| Ni(111) | −3.29 | 2.48 | 1.95 | 1.63 | 4.04 | 2.10 | 4.09 | 2.30 | 0.38 |
| Ir(111) | −5.26 | 1.91 | −0.37 | 0.08 | 2.18 | 2.54 | −0.22 | −0.18 | 0.83 |
| MgO(111)$_{smH}$ | −5.02 | 1.86 | 0.81 | 0.46 | 2.64 | 1.83 | −3.78 | −0.02 | 0.80 |
| MgO(111)$_{smT}$ | −8.38 | 1.81 | 0.86 | 0.73 | 2.89 | 2.04 | −2.50 | −0.03 | 0.89 |
| MgO(111)$_{smE}$ | −6.24 | 1.84 | 0.94 | 1.80 | 4.05 | 3.11 | 3.03 | 2.12 | 0.96 |
| MgO(111)$_{lmH}$ | −4.28 | 1.88 | 0.88 | 0.23 | 2.34 | 1.46 | 2.04 | 0.40 | 0.79 |
| MgO(111)$_{lmT}$ | −8.98 | 1.77 | 0.63 | 0.52 | 2.56 | 1.92 | 0.45 | 0.68 | 1.00 |
| MgO(111)$_{lmE}$ | −6.46 | 1.83 | 0.76 | 1.33 | 3.41 | 2.65 | 3.68 | 2.11 | 0.97 |
| MgO(001) | −4.89 | 1.83 | 0.91 | 1.36 | 4.62 | 3.72 | 2.25 | 1.88 | 0.78 |
| | | | | Ir | | | | | |
| NSV | −3.97 | 2.01 | 1.28 | 1.56 | | | 1.83 | 0.74 | 0.59 |
| Cu(111) | −3.95 | 2.02 | 1.21 | 0.94 | 3.68 | 2.47 | 0.00 | 0.00 | 0.63 |
| Ni(111) | −3.12 | 2.05 | 1.39 | 1.11 | 3.50 | 2.11 | 2.42 | 0.63 | 0.48 |
| Ir(111) | −6.33 | 2.02 | −0.69 | −0.16 | 1.94 | 2.64 | −0.02 | 0.00 | 0.75 |
| MgO(111)$_{smH}$ | −5.64 | 2.02 | 0.92 | 0.62 | 2.76 | 1.83 | −4.50 | −0.03 | 0.74 |
| MgO(111)$_{smT}$ | −9.28 | 1.96 | 1.04 | 0.90 | 3.02 | 1.98 | −2.50 | 0.16 | 0.85 |
| MgO(111)$_{smE}$ | −6.27 | 1.92 | 1.06 | 1.90 | 4.14 | 3.08 | −0.24 | −0.01 | 0.92 |
| MgO(111)$_{lmH}$ | −4.77 | 2.00 | 1.04 | 0.34 | 2.46 | 1.42 | 1.46 | 0.15 | 0.68 |
| MgO(111)$_{lmT}$ | −9.72 | 1.91 | 0.96 | 0.81 | 2.85 | 1.89 | 0.56 | 0.23 | 0.94 |
| MgO(111)$_{lmE}$ | −6.53 | 1.92 | 0.91 | 1.45 | 3.53 | 2.62 | 1.04 | −0.01 | 0.95 |
| MgO(001) | −4.16 | 2.00 | 1.26 | 1.56 | 4.80 | 3.55 | 1.42 | 0.65 | 0.59 |
| | | | | Mn | | | | | |
| NSV | −3.38 | 1.79 | 0.84 | 1.24 | | | 0.51 | 0.54 | 0.96 |
| Cu(111) | −4.05 | 1.95 | 0.98 | 1.15 | 4.12 | 3.14 | 4.41 | 4.08 | 1.15 |
| Ni(111) | −1.64 | 2.61 | 2.11 | 1.82 | 4.23 | 2.11 | 6.48 | 4.64 | 0.63 |
| Ir(111) | −5.32 | 1.95 | −0.36 | 0.12 | 2.21 | 2.57 | 3.78 | 3.51 | 1.19 |
| MgO(111)$_{smH}$ | −4.07 | 1.91 | 0.90 | 0.55 | 2.74 | 1.84 | −4.61 | −2.11 | 1.10 |
| MgO(111)$_{smT}$ | −7.63 | 1.82 | 0.87 | 0.78 | 2.91 | 2.03 | −4.50 | −2.63 | 1.26 |
| MgO(111)$_{smE}$ | −5.34 | 1.79 | 0.91 | 1.71 | 3.95 | 3.04 | −2.04 | −1.29 | 1.17 |
| MgO(111)$_{lmH}$ | −3.60 | 1.97 | 1.03 | 0.36 | 2.47 | 1.44 | 3.98 | 3.14 | 1.13 |
| MgO(111)$_{lmT}$ | −9.23 | 1.79 | 0.65 | 0.53 | 2.57 | 1.92 | 1.50 | 2.70 | 1.31 |
| MgO(111)$_{lmE}$ | −5.87 | 1.95 | 1.02 | 1.58 | 3.67 | 2.65 | −3.61 | −4.13 | 1.23 |
| MgO(001) | −4.05 | 1.96 | 1.20 | 1.57 | 4.82 | 3.62 | 5.13 | 4.16 | 1.01 |

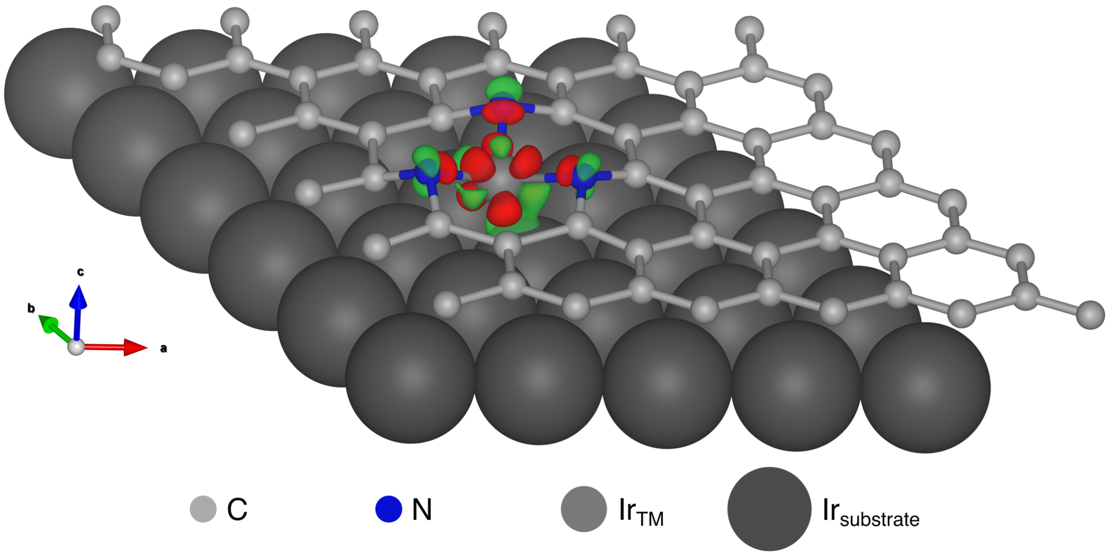


**Figure S19: Charge density difference induced by the adsorption of a single Ir atom on NSV@Ir(111).** Green/red isosurfaces ($\pm 2\times10^{-2}$ e/Å$^3$) indicate electron accumulation/depletion. To facilitate visualization, the diameter of the substrate atoms in the topmost layer has been increased, and substrate atoms in the lower layers have been omitted. Plotted using VESTA [5].

## S6 TM dimers

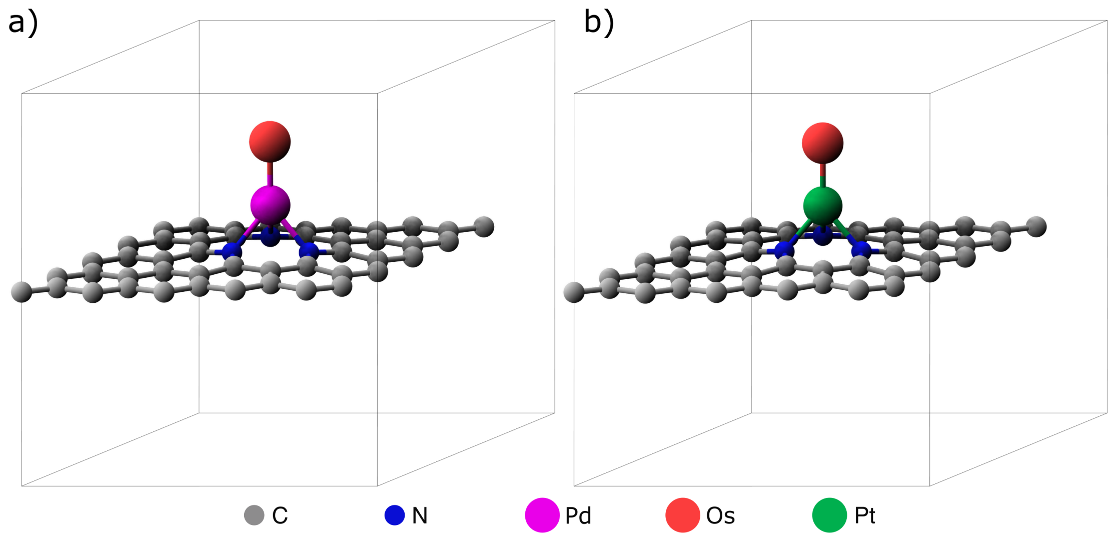


**Figure S20: Os atom adsorbed on freestanding (a)** Pd@NSV and **(b)** Pt@NSV. Rendered using POET

**Table S8: Scalar-relativistic properties of Os–TM and Ir–TM dimers adsorbed on freestanding and supported NSV–graphene.** The adsorption energy $E_a$ of the upper atom atop the pre-adsorbed TM atom (in eV). The TM–TM bond length $d_d$, average TM–N bond length $d_{bl}$, TM–N vertical distance (along the surface normal) $d_{van}$, and between the lower TM atom and all atoms forming the NSV–graphene $d_{vag}$, as well as between the lower TM atom and the topmost substrate layer $d_{vas}$ (all in Å). The dimer tilt angle, defined as the angle between the TM–TM axis and the surface normal (in °). The reduced total magnetic moment of the supercell $\mu_{\mathrm{red}}$ and the local magnetic moments on the lower $\mu_{\mathrm{TM_B}}$ and upper $\mu_{\mathrm{TM_T}}$ TM atoms (in $\mu_{\mathrm{B}}$). Bond order (BO) between the two TM atoms, evaluated considering molecular orbitals spanning two atoms ($BO_2$), three atoms ($BO_3$), and five atoms ($BO_5$). See Figure S1 for the notation.

| Substrate | $E_a^{\mathrm{T}}$ | $d_d$ | $d_{bl}$ | $d_{van}$ | $d_{vag}$ | $d_{vas}$ | tilt | $\mu_{\mathrm{red}}$ | $\mu_{\mathrm{TM_B}}$ | $\mu_{\mathrm{TM_T}}$ | $q_{\mathrm{TM_B}}$ | $q_{\mathrm{TM_T}}$ | $BO_2$ | $BO_3$ | $BO_5$ |
|---|---|---|---|---|---|---|---|---|---|---|---|---|---|---|---|
| | | | | | | | OsPd | | | | | | | | |
| NSV | −2.83 | 2.26 | 2.15 | 1.50 | 1.78 | | 0.5 | 5.00 | 0.86 | 3.32 | 0.40 | 0.01 | −0.17 | 0.53 | 1.10 |
| Cu(111) | −2.70 | 2.34 | 2.23 | 1.53 | 1.38 | 3.97 | 0.2 | 3.81 | 0.51 | 3.11 | 0.30 | 0.00 | 0.92 | 1.71 | 1.47 |
| Ni(111) | −3.24 | 2.33 | 2.26 | 1.67 | 1.44 | 3.75 | 0.4 | 5.43 | 0.55 | 3.12 | 0.22 | 0.03 | 0.98 | 1.75 | 1.42 |
| Ir(111) | −2.95 | 2.32 | 2.26 | 1.38 | 1.52 | 3.55 | 1.6 | 4.25 | 0.78 | 3.30 | 0.33 | 0.03 | 0.30 | 1.31 | 1.61 |
| $MgO(111)_{\mathrm{smH}}$ | −2.56 | 2.37 | 2.43 | 1.62 | 1.22 | 3.52 | 25.9 | −8.23 | −0.40 | −3.31 | 0.24 | 0.25 | −1.15 | −0.17 | 0.61 |
| $MgO(111)_{\mathrm{smT}}$ | −2.66 | 2.32 | 2.07 | 1.33 | 1.41 | 3.73 | 12.2 | 3.44 | 0.21 | 3.09 | 0.49 | 0.19 | −0.32 | −0.04 | 0.17 |
| $MgO(111)_{\mathrm{smE}}$ | −2.91 | 2.23 | 2.06 | 1.36 | 2.13 | 4.43 | 1.6 | −1.82 | 0.08 | −2.18 | 0.48 | 0.23 | 0.94 | 0.86 | 0.74 |
| $MgO(111)_{\mathrm{lmH}}$ | −3.95 | 2.35 | 3.13 | 2.11 | 1.33 | 3.51 | 5.9 | 0.98 | 0.14 | 3.08 | 0.19 | 0.27 | −1.08 | −0.13 | 0.62 |
| $MgO(111)_{\mathrm{lmT}}$ | −2.25 | 2.34 | 2.00 | 1.13 | 0.98 | 3.15 | 5.5 | 0.57 | 0.20 | 3.16 | 0.59 | 0.24 | −0.17 | −0.10 | −0.05 |
| $MgO(111)_{\mathrm{lmE}}$ | −2.81 | 2.30 | 2.11 | 1.33 | 1.84 | 4.03 | 0.5 | −3.50 | −0.54 | −3.32 | 0.47 | 0.26 | −0.98 | −0.34 | 0.18 |
| MgO(001) | −3.03 | 2.32 | 2.20 | 1.59 | 1.81 | 5.06 | 2.7 | 5.00 | 0.85 | 3.31 | 0.36 | 0.04 | −0.79 | 0.05 | 0.72 |

| Substrate | $E_a^{\mathrm{T}}$ | $d_d$ | $d_{bl}$ | $d_{van}$ | $d_{vag}$ | $d_{vas}$ | tilt | $\mu_{\mathrm{red}}$ | $\mu_{\mathrm{TM_B}}$ | $\mu_{\mathrm{TM_T}}$ | $q_{\mathrm{TM_B}}$ | $q_{\mathrm{TM_T}}$ | $BO_2$ | $BO_3$ | $BO_5$ |
|---|---|---|---|---|---|---|---|---|---|---|---|---|---|---|---|
| OsPt | | | | | | | | | | | | | | | |
| NSV | −4.05 | 2.24 | 2.11 | 1.44 | 1.76 | | 0.1 | 1.40 | −0.08 | 1.46 | 0.43 | −0.09 | 1.69 | 2.08 | 1.36 |
| Cu(111) | −3.88 | 2.28 | 2.18 | 1.48 | 1.36 | 3.93 | 0.4 | 3.01 | 0.25 | 2.49 | 0.23 | 0.13 | 1.52 | 2.02 | 1.35 |
| Ni(111) | −4.13 | 2.30 | 2.22 | 1.62 | 1.42 | 3.70 | 0.1 | 5.09 | 0.44 | 2.77 | 0.09 | 0.17 | 0.41 | 1.14 | 1.50 |
| Ir(111) | −2.61 | 2.30 | 2.25 | 1.40 | 1.54 | 3.56 | 2.0 | 3.79 | 0.51 | 2.81 | 0.25 | 0.07 | 1.08 | 1.91 | 1.57 |
| $MgO(111)_{smH}$ | −5.85 | 2.41 | 2.58 | 1.66 | 1.25 | 3.49 | 78.9 | −3.50 | 0.31 | 1.22 | 0.12 | 0.55 | −0.73 | 1.17 | 1.84 |
| $MgO(111)_{smT}$ | −6.44 | 2.28 | 2.09 | 1.30 | 1.30 | 3.54 | 25.1 | −6.16 | 0.05 | −2.31 | 0.44 | 0.23 | 0.20 | 0.44 | 0.64 |
| $MgO(111)_{smE}$ | −4.56 | 2.23 | 2.06 | 1.37 | 2.14 | 4.43 | 1.4 | 2.21 | −0.05 | 1.92 | 0.50 | 0.26 | 1.26 | 1.20 | 0.96 |
| $MgO(111)_{lmH}$ | −3.61 | 2.33 | 3.00 | 2.09 | 1.35 | 3.58 | 7.5 | 3.92 | 0.29 | 2.96 | 0.06 | 0.34 | −0.94 | 0.37 | 1.41 |
| $MgO(111)_{lmT}$ | −3.22 | 2.27 | 1.97 | 1.13 | 0.95 | 3.12 | 1.8 | 1.12 | −0.06 | 2.35 | 0.65 | 0.23 | 0.50 | 0.41 | 0.34 |
| $MgO(111)_{lmE}$ | −4.31 | 2.24 | 2.07 | 1.27 | 1.79 | 3.98 | 0.7 | 2.86 | −0.06 | 1.94 | 0.45 | 0.31 | 1.16 | 1.15 | 1.03 |
| MgO(001) | −4.32 | 2.27 | 2.16 | 1.53 | 1.79 | 5.04 | 8.7 | 3.02 | 0.27 | 2.31 | 0.33 | 0.00 | 0.88 | 1.22 | 1.50 |
| OsCo | | | | | | | | | | | | | | | |
| NSV | −3.10 | 2.07 | 1.88 | 1.07 | 1.37 | | 0.7 | 2.04 | 0.11 | 1.88 | 0.69 | −0.26 | 1.70 | 2.14 | 1.87 |
| Cu(111) | −2.90 | 2.06 | 1.89 | 0.95 | 1.13 | 4.05 | 0.0 | 2.15 | 0.10 | 1.92 | 0.70 | −0.21 | 1.96 | 2.40 | 1.50 |
| Ni(111) | −3.65 | 2.10 | 1.96 | 1.24 | 1.05 | 3.34 | 0.1 | 5.65 | 1.26 | 2.63 | 0.65 | −0.20 | 2.44 | 2.68 | 1.67 |
| Ir(111) | −2.61 | 2.24 | 2.01 | −0.83 | −0.32 | 1.75 | 6.6 | 5.78 | 1.53 | 2.58 | 0.71 | 0.52 | 1.20 | 1.64 | 1.71 |
| $MgO(111)_{smH}$ | −2.81 | 2.24 | 1.92 | 0.94 | 0.59 | 2.88 | 4.2 | 0.51 | 0.63 | 3.15 | 0.61 | 0.21 | −0.69 | 0.23 | 0.96 |
| $MgO(111)_{smT}$ | −1.05 | 2.11 | 1.82 | 1.01 | 1.07 | 3.38 | 21.8 | 1.50 | 0.13 | 2.63 | 0.80 | 0.01 | 0.63 | 0.92 | 1.15 |
| $MgO(111)_{smE}$ | −3.37 | 2.10 | 1.84 | 1.03 | 1.79 | 4.08 | 1.2 | 2.91 | 0.11 | 2.53 | 0.76 | 0.06 | 0.83 | 1.19 | 1.16 |
| $MgO(111)_{lmH}$ | −6.89 | 2.61 | 1.91 | 0.95 | 0.26 | 2.45 | 63.1 | −0.50 | 0.50 | 0.32 | 0.74 | 0.38 | −0.44 | 0.72 | 1.39 |
| $MgO(111)_{lmT}$ | −6.10 | 2.42 | 1.79 | 0.67 | 0.50 | 2.65 | 65.7 | −0.52 | 0.72 | 0.21 | 0.89 | 0.50 | −0.69 | 1.28 | 2.36 |
| $MgO(111)_{lmE}$ | −3.14 | 2.10 | 1.86 | 0.92 | 1.42 | 3.61 | 0.2 | 3.50 | 0.01 | 2.52 | 0.78 | 0.05 | 0.40 | 0.59 | 0.75 |
| MgO(001) | −3.48 | 2.07 | 1.86 | 1.06 | 1.35 | 4.64 | 0.9 | 2.09 | 0.14 | 1.93 | 0.72 | −0.28 | 1.62 | 2.01 | 1.82 |

| Substrate | $E_a^{T}$ | $d_d$ | $d_{bl}$ | $d_{van}$ | $d_{vag}$ | $d_{vas}$ | tilt | $\mu_{red}$ | $\mu_{TM_B}$ | $\mu_{TM_T}$ | $q_{TM_B}$ | $q_{TM_T}$ | $BO_2$ | $BO_3$ | $BO_5$ |
|---|---|---|---|---|---|---|---|---|---|---|---|---|---|---|---|
| **OsIr** | | | | | | | | | | | | | | | |
| NSV | −5.19 | 2.16 | 2.06 | 1.36 | 1.73 | | 0.2 | −2.08 | −0.30 | −1.69 | 0.63 | −0.18 | 0.95 | 2.45 | 2.11 |
| Cu(111) | −5.04 | 2.18 | 2.06 | 1.25 | 1.47 | 4.13 | 0.9 | 2.09 | 0.13 | 1.58 | 0.58 | −0.06 | 2.32 | 2.52 | 1.51 |
| Ni(111) | −5.12 | 2.19 | 2.08 | 1.44 | 1.30 | 3.51 | 0.3 | 2.26 | 0.16 | 1.62 | 0.43 | −0.05 | 3.08 | 2.83 | 1.70 |
| Ir(111) | −1.99 | 2.47 | 2.07 | −0.84 | −0.24 | 1.78 | 1.1 | 4.26 | 0.10 | 3.01 | 0.63 | 0.33 | 1.06 | 1.00 | 0.60 |
| $MgO(111)_{smH}$ | −3.34 | 2.22 | 2.14 | 1.27 | 0.95 | 3.24 | 11.6 | −2.36 | −0.11 | 1.58 | 0.56 | 0.09 | 0.81 | 1.06 | 1.07 |
| $MgO(111)_{smT}$ | −3.52 | 2.21 | 2.05 | 1.23 | 1.11 | 3.36 | 19.9 | −2.50 | 0.15 | −0.50 | 0.69 | 0.06 | 1.50 | 1.72 | 1.24 |
| $MgO(111)_{smE}$ | −4.92 | 2.20 | 2.01 | 1.27 | 2.09 | 4.38 | 1.9 | 2.12 | 0.06 | 2.08 | 0.71 | 0.15 | 0.60 | 1.88 | 2.06 |
| $MgO(111)_{lmH}$ | −7.04 | 2.38 | 2.08 | 1.19 | 0.48 | 2.67 | 64.1 | 0.25 | 0.40 | 1.10 | 0.56 | 0.41 | −0.96 | 0.87 | 1.59 |
| $MgO(111)_{lmT}$ | −4.13 | 2.23 | 1.94 | 1.08 | 0.89 | 3.06 | 1.4 | 1.53 | 0.02 | 2.18 | 0.85 | 0.14 | 0.50 | 0.59 | 0.66 |
| $MgO(111)_{lmE}$ | −4.68 | 2.19 | 2.01 | 1.17 | 1.72 | 3.90 | 1.1 | 3.23 | 0.04 | 2.02 | 0.73 | 0.14 | 1.20 | 1.42 | 1.59 |
| MgO(001) | −5.44 | 2.17 | 2.03 | 1.31 | 1.69 | 4.99 | 0.7 | 1.92 | 0.17 | 1.57 | 0.60 | −0.15 | 0.77 | 2.37 | 2.07 |
| **OsMn** | | | | | | | | | | | | | | | |
| NSV | −4.56 | 1.92 | 1.93 | 1.14 | 1.48 | | 0.1 | 2.00 | 0.96 | 0.83 | 0.94 | −0.39 | 2.80 | 3.39 | 2.14 |
| Cu(111) | −4.06 | 1.92 | 1.93 | 1.00 | 1.20 | 4.10 | 0.0 | 1.91 | 0.90 | 0.93 | 0.95 | −0.32 | 3.19 | 3.43 | 2.06 |
| Ni(111) | −5.31 | 1.91 | 1.95 | 1.21 | 1.07 | 3.29 | 0.7 | 2.18 | 1.08 | 0.79 | 0.89 | −0.38 | 3.59 | 3.10 | 1.86 |
| Ir(111) | −1.47 | 2.14 | 1.95 | −0.27 | 0.26 | 2.27 | 1.9 | 5.73 | 2.12 | 2.47 | 1.02 | 0.17 | 0.47 | 0.84 | 1.13 |
| $MgO(111)_{smH}$ | −3.71 | 2.05 | 1.95 | 0.94 | 0.64 | 2.93 | 32.0 | −2.50 | −0.01 | 0.69 | 0.89 | 0.20 | −0.53 | 1.48 | 2.30 |
| $MgO(111)_{smT}$ | −2.65 | 2.01 | 1.87 | 0.98 | 0.91 | 3.17 | 17.2 | 1.50 | 0.30 | 1.79 | 1.06 | −0.10 | 2.02 | 2.08 | 1.82 |
| $MgO(111)_{smE}$ | −4.09 | 1.99 | 1.89 | 1.09 | 1.87 | 4.17 | 2.9 | 0.93 | −0.80 | 1.83 | 1.04 | −0.07 | 1.70 | 2.04 | 1.69 |
| $MgO(111)_{lmH}$ | −7.14 | 2.19 | 2.03 | 1.17 | 0.45 | 2.64 | 61.6 | −2.50 | −1.89 | 0.24 | 0.98 | 0.26 | −0.70 | 0.74 | 1.74 |
| $MgO(111)_{lmT}$ | −4.70 | 2.31 | 1.82 | 0.82 | 0.63 | 2.78 | 58.2 | −2.39 | −2.22 | 1.98 | 1.20 | 0.27 | 0.73 | 0.59 | 0.48 |
| $MgO(111)_{lmE}$ | −3.66 | 2.00 | 1.89 | 0.95 | 1.46 | 3.64 | 2.8 | 2.51 | −0.71 | 1.80 | 1.05 | −0.07 | 1.45 | 2.04 | 1.74 |
| MgO(001) | −4.31 | 1.96 | 1.91 | 1.12 | 1.43 | 4.72 | 0.3 | 2.00 | 0.92 | 0.97 | 0.96 | −0.41 | 2.62 | 3.21 | 2.10 |

| Substrate | $E_a^{\mathrm{T}}$ | $d_d$ | $d_{bl}$ | $d_{van}$ | $d_{vag}$ | $d_{vas}$ | tilt | $\mu_{\mathrm{red}}$ | $\mu_{\mathrm{TM_B}}$ | $\mu_{\mathrm{TM_T}}$ | $q_{\mathrm{TM_B}}$ | $q_{\mathrm{TM_T}}$ | $BO_2$ | $BO_3$ | $BO_5$ |
|---|---|---|---|---|---|---|---|---|---|---|---|---|---|---|---|
| | | | | | | | IrPd | | | | | | | | |
| NSV | −2.94 | 2.33 | 2.17 | 1.53 | 1.79 | | 1.9 | 4.00 | 0.94 | 2.24 | 0.46 | −0.14 | −0.06 | 0.53 | 1.00 |
| Cu(111) | −2.69 | 2.36 | 2.23 | 1.53 | 1.32 | 3.91 | 1.0 | −2.43 | −0.55 | −2.10 | 0.40 | −0.18 | 1.37 | 1.98 | 1.70 |
| Ni(111) | −3.25 | 2.36 | 2.24 | 1.65 | 1.40 | 3.72 | 0.1 | 4.33 | 0.54 | 2.12 | 0.27 | −0.14 | 1.42 | 2.06 | 1.61 |
| Ir(111) | −2.84 | 2.33 | 2.24 | 1.35 | 1.48 | 3.51 | 2.0 | −3.42 | −0.82 | −2.18 | 0.39 | −0.17 | 0.55 | 1.22 | 1.38 |
| $MgO(111)_{smH}$ | −2.56 | 2.38 | 2.37 | 1.52 | 1.14 | 3.43 | 28.6 | −0.81 | 0.44 | 2.25 | 0.33 | 0.06 | −0.34 | 0.15 | 0.54 |
| $MgO(111)_{smT}$ | −2.61 | 2.33 | 2.05 | 1.29 | 1.35 | 3.67 | 12.3 | 1.50 | 0.07 | 1.85 | 0.58 | −0.03 | 0.12 | 0.23 | 0.32 |
| $MgO(111)_{smE}$ | −2.66 | 2.31 | 2.07 | 1.37 | 2.13 | 4.43 | 5.9 | −0.57 | 0.23 | −1.18 | 0.58 | 0.02 | 0.63 | 0.64 | 0.51 |
| $MgO(111)_{lmH}$ | −6.61 | 2.68 | 2.06 | 1.12 | 0.44 | 2.61 | 68.4 | −0.70 | 0.03 | 0.00 | 0.51 | 0.25 | −2.36 | −1.26 | −0.39 |
| $MgO(111)_{lmT}$ | −7.01 | 2.59 | 2.01 | 1.06 | 0.84 | 2.99 | 72.0 | −2.69 | 0.01 | 0.03 | 0.68 | 0.19 | −2.02 | −0.97 | −0.14 |
| $MgO(111)_{lmE}$ | −2.66 | 2.30 | 2.08 | 1.28 | 1.78 | 3.97 | 1.2 | 4.18 | 0.55 | 2.14 | 0.56 | 0.06 | −0.60 | −0.24 | 0.04 |
| MgO(001) | −3.08 | 2.32 | 2.16 | 1.53 | 1.75 | 5.02 | 1.5 | 4.00 | 0.93 | 2.24 | 0.46 | −0.15 | −0.50 | 0.10 | 0.59 |
| | | | | | | | IrPt | | | | | | | | |
| NSV | −4.22 | 2.25 | 2.11 | 1.44 | 1.75 | | 0.9 | 0.00 | −0.03 | 0.02 | 0.46 | −0.27 | 0.27 | 1.36 | 1.41 |
| Cu(111) | −3.96 | 2.32 | 2.17 | 1.47 | 1.31 | 3.90 | 0.2 | 1.75 | 0.16 | 1.57 | 0.42 | −0.20 | 0.57 | 1.28 | 1.59 |
| Ni(111) | −4.20 | 2.32 | 2.19 | 1.59 | 1.39 | 3.67 | 0.1 | 3.90 | 0.38 | 1.77 | 0.25 | −0.12 | 0.84 | 1.48 | 1.68 |
| Ir(111) | −2.66 | 2.30 | 2.21 | 1.28 | 1.53 | 3.55 | 17.6 | 2.10 | 0.38 | 1.43 | 0.44 | −0.18 | 1.36 | 1.73 | 1.10 |
| $MgO(111)_{smH}$ | −5.37 | 2.38 | 2.33 | 1.34 | 0.97 | 3.21 | 60.5 | −5.00 | 0.03 | 0.19 | 0.38 | 0.13 | 0.03 | 1.13 | 1.33 |
| $MgO(111)_{smT}$ | −5.80 | 2.28 | 2.07 | 1.31 | 1.20 | 3.45 | 24.0 | −0.50 | 0.03 | 1.43 | 0.54 | 0.00 | 0.25 | 0.45 | 0.61 |
| $MgO(111)_{smE}$ | −4.39 | 2.23 | 2.05 | 1.34 | 2.12 | 4.42 | 1.8 | −0.82 | 0.05 | −0.90 | 0.57 | 0.06 | 1.23 | 1.36 | 1.08 |
| $MgO(111)_{lmH}$ | −2.85 | 2.38 | 2.17 | 1.32 | 0.65 | 2.88 | 12.6 | −1.57 | −0.11 | −1.89 | 0.35 | 0.11 | −0.41 | −0.20 | −0.03 |
| $MgO(111)_{lmT}$ | −3.27 | 2.29 | 1.97 | 1.11 | 0.94 | 3.11 | 1.5 | −0.47 | −0.03 | 1.30 | 0.72 | 0.04 | 0.61 | 0.53 | 0.46 |
| $MgO(111)_{lmE}$ | −4.13 | 2.23 | 2.05 | 1.25 | 1.78 | 3.96 | 0.8 | 1.77 | −0.05 | 0.87 | 0.59 | 0.03 | 1.13 | 1.62 | 1.21 |
| MgO(001) | −4.36 | 2.30 | 2.13 | 1.49 | 1.73 | 4.99 | 8.4 | 2.00 | 0.26 | 1.38 | 0.43 | −0.19 | 0.94 | 1.58 | 1.90 |

| Substrate | $E_a^{\mathrm{T}}$ | $d_d$ | $d_{bl}$ | $d_{van}$ | $d_{vag}$ | $d_{vas}$ | tilt | $\mu_{\mathrm{red}}$ | $\mu_{\mathrm{TM_B}}$ | $\mu_{\mathrm{TM_T}}$ | $q_{\mathrm{TM_B}}$ | $q_{\mathrm{TM_T}}$ | $BO_2$ | $BO_3$ | $BO_5$ |
|---|---|---|---|---|---|---|---|---|---|---|---|---|---|---|---|
| | | | | | | | IrCo | | | | | | | | |
| NSV | −3.19 | 2.08 | 1.86 | 1.04 | 1.33 | | 0.0 | 1.14 | 0.37 | 0.72 | 0.79 | −0.46 | 1.18 | 2.00 | 1.60 |
| Cu(111) | −2.95 | 2.07 | 1.89 | 0.94 | 1.10 | 4.03 | 0.0 | 1.31 | 0.44 | 0.65 | 0.78 | −0.38 | 1.74 | 1.92 | 1.24 |
| Ni(111) | −3.84 | 2.13 | 1.93 | 1.19 | 0.99 | 3.30 | 0.1 | 5.18 | 1.74 | 1.84 | 0.74 | −0.40 | 2.57 | 3.09 | 2.16 |
| Ir(111) | −1.83 | 2.33 | 2.04 | −0.91 | −0.39 | 1.67 | 5.0 | 4.56 | 1.54 | 1.72 | 0.72 | 0.41 | 1.18 | 1.75 | 1.88 |
| $MgO(111)_{smH}$ | −2.78 | 2.24 | 1.92 | 0.94 | 0.58 | 2.88 | 6.2 | −5.68 | −0.50 | −2.02 | 0.68 | −0.06 | −0.19 | 0.67 | 1.36 |
| $MgO(111)_{smT}$ | −6.37 | 2.35 | 1.83 | 0.92 | 0.85 | 3.07 | 73.6 | −3.50 | 0.05 | 0.02 | 0.83 | 0.08 | −2.22 | 0.52 | 2.70 |
| $MgO(111)_{smE}$ | −3.35 | 2.11 | 1.83 | 1.01 | 1.75 | 4.04 | 0.8 | −1.61 | −0.13 | −1.48 | 0.85 | −0.14 | 0.23 | 1.71 | 1.77 |
| $MgO(111)_{lmH}$ | −6.93 | 2.40 | 1.89 | 0.94 | 0.28 | 2.47 | 60.2 | −0.48 | 0.31 | 0.12 | 0.76 | 0.15 | −2.32 | −0.13 | 1.62 |
| $MgO(111)_{lmT}$ | −6.61 | 2.53 | 1.81 | 0.85 | 0.59 | 2.74 | 68.1 | −3.21 | −0.07 | 0.00 | 0.85 | 0.33 | −2.26 | −0.78 | 0.41 |
| $MgO(111)_{lmE}$ | −3.11 | 2.12 | 1.84 | 0.88 | 1.36 | 3.55 | 0.1 | 2.50 | 0.08 | 1.48 | 0.86 | −0.13 | −0.11 | 1.53 | 1.56 |
| MgO(001) | −3.19 | 2.17 | 1.97 | 1.23 | 1.49 | 4.77 | 13.0 | 1.11 | −0.07 | 1.25 | 0.78 | −0.42 | 1.34 | 1.91 | 1.88 |
| | | | | | | | IrIr | | | | | | | | |
| NSV | −5.30 | 2.20 | 2.03 | 1.31 | 1.69 | | 0.1 | 1.00 | 0.28 | 0.61 | 0.67 | −0.32 | 0.55 | 2.28 | 2.07 |
| Cu(111) | −5.12 | 2.19 | 2.04 | 1.22 | 1.45 | 4.13 | 0.1 | 1.21 | 0.26 | 0.59 | 0.70 | −0.27 | 1.36 | 2.15 | 1.53 |
| Ni(111) | −5.16 | 2.20 | 2.05 | 1.39 | 1.27 | 3.47 | 0.3 | 0.80 | 0.10 | 0.37 | 0.63 | −0.34 | 0.82 | 2.07 | 1.57 |
| Ir(111) | −1.99 | 2.50 | 2.06 | −0.82 | −0.23 | 1.79 | 5.5 | 2.79 | 0.01 | 1.90 | 0.65 | 0.19 | 1.21 | 1.13 | 0.68 |
| $MgO(111)_{smH}$ | −3.43 | 2.24 | 2.11 | 1.23 | 0.90 | 3.20 | 12.8 | −2.75 | 0.00 | 0.85 | 0.66 | −0.07 | 0.51 | 1.22 | 1.30 |
| $MgO(111)_{smT}$ | −3.73 | 2.23 | 2.03 | 1.22 | 1.09 | 3.35 | 21.5 | −1.50 | 0.08 | 0.96 | 0.78 | −0.10 | 0.26 | 1.80 | 1.92 |
| $MgO(111)_{smE}$ | −4.85 | 2.22 | 1.99 | 1.24 | 2.05 | 4.35 | 2.1 | 1.18 | 0.11 | 1.13 | 0.84 | −0.08 | 0.31 | 2.15 | 2.21 |
| $MgO(111)_{lmH}$ | −3.33 | 2.33 | 2.09 | 1.24 | 0.57 | 2.80 | 8.1 | 3.16 | 0.21 | 1.64 | 0.59 | 0.05 | −0.83 | 0.56 | 1.13 |
| $MgO(111)_{lmT}$ | −4.18 | 2.22 | 1.93 | 1.06 | 0.87 | 3.04 | 0.4 | −1.50 | 0.07 | −1.04 | 0.97 | −0.08 | 0.12 | 0.50 | 0.49 |
| $MgO(111)_{lmE}$ | −4.60 | 2.19 | 1.99 | 1.14 | 1.68 | 3.86 | 0.9 | 2.26 | 0.10 | 0.98 | 0.86 | −0.08 | −0.13 | 1.57 | 1.97 |
| MgO(001) | −5.54 | 2.21 | 2.02 | 1.31 | 1.68 | 4.97 | 1.0 | 1.10 | 0.29 | 0.70 | 0.68 | −0.32 | 0.29 | 2.21 | 2.30 |

| Substrate | $E_a^{\mathrm{T}}$ | $d_d$ | $d_{bl}$ | $d_{van}$ | $d_{vag}$ | $d_{vas}$ | tilt | $\mu_{\mathrm{red}}$ | $\mu_{\mathrm{TM_B}}$ | $\mu_{\mathrm{TM_T}}$ | $q_{\mathrm{TM_B}}$ | $q_{\mathrm{TM_T}}$ | $BO_2$ | $BO_3$ | $BO_5$ |
|---|---|---|---|---|---|---|---|---|---|---|---|---|---|---|---|
| | | | | | | | IrMn | | | | | | | | |
| NSV | −4.71 | 2.01 | 1.91 | 1.12 | 1.44 | | 0.6 | 2.37 | 1.81 | 0.40 | 1.09 | −0.52 | 0.77 | 1.55 | 1.59 |
| Cu(111) | −4.17 | 1.99 | 1.89 | 0.93 | 1.13 | 4.01 | 0.0 | 1.07 | 1.29 | 0.32 | 1.09 | −0.44 | 1.46 | 1.96 | 1.36 |
| Ni(111) | −5.39 | 2.01 | 1.93 | 1.20 | 1.06 | 3.28 | 0.6 | 2.36 | 1.91 | 0.40 | 1.05 | −0.52 | 2.00 | 1.77 | 1.06 |
| Ir(111) | −1.71 | 2.24 | 1.93 | −0.17 | 0.33 | 2.34 | 2.5 | 5.30 | 2.71 | 1.69 | 1.09 | −0.03 | 0.06 | 0.54 | 0.91 |
| $MgO(111)_{smH}$ | −3.72 | 2.14 | 1.95 | 0.95 | 0.64 | 2.93 | 34.2 | −3.50 | −0.67 | 0.05 | 0.97 | 0.05 | −1.81 | 0.20 | 1.80 |
| $MgO(111)_{smT}$ | −6.62 | 2.29 | 1.91 | 1.04 | 0.96 | 3.18 | 75.6 | −7.50 | −2.68 | −0.27 | 1.19 | 0.00 | −1.35 | −0.08 | 0.65 |
| $MgO(111)_{smE}$ | −4.33 | 1.99 | 1.88 | 1.08 | 1.86 | 4.15 | 2.1 | 2.37 | 1.42 | 0.58 | 1.12 | −0.27 | 0.28 | 1.62 | 1.74 |
| $MgO(111)_{lmH}$ | −7.43 | 2.40 | 2.01 | 1.12 | 0.41 | 2.61 | 63.1 | 1.36 | 2.92 | 0.15 | 1.09 | 0.07 | −1.38 | −0.33 | 0.46 |
| $MgO(111)_{lmT}$ | −2.99 | 2.15 | 1.80 | 0.77 | 0.59 | 2.76 | 3.8 | 2.50 | 1.63 | 1.32 | 1.22 | −0.06 | −0.86 | 0.27 | 0.84 |
| $MgO(111)_{lmE}$ | −3.91 | 2.00 | 1.88 | 0.94 | 1.44 | 3.62 | 1.1 | 2.90 | 1.38 | 0.57 | 1.12 | −0.25 | −0.09 | 1.43 | 1.83 |
| MgO(001) | −4.49 | 2.00 | 1.90 | 1.11 | 1.42 | 4.70 | 0.7 | 2.18 | 1.69 | 0.42 | 1.08 | −0.51 | 0.34 | 1.45 | 1.66 |

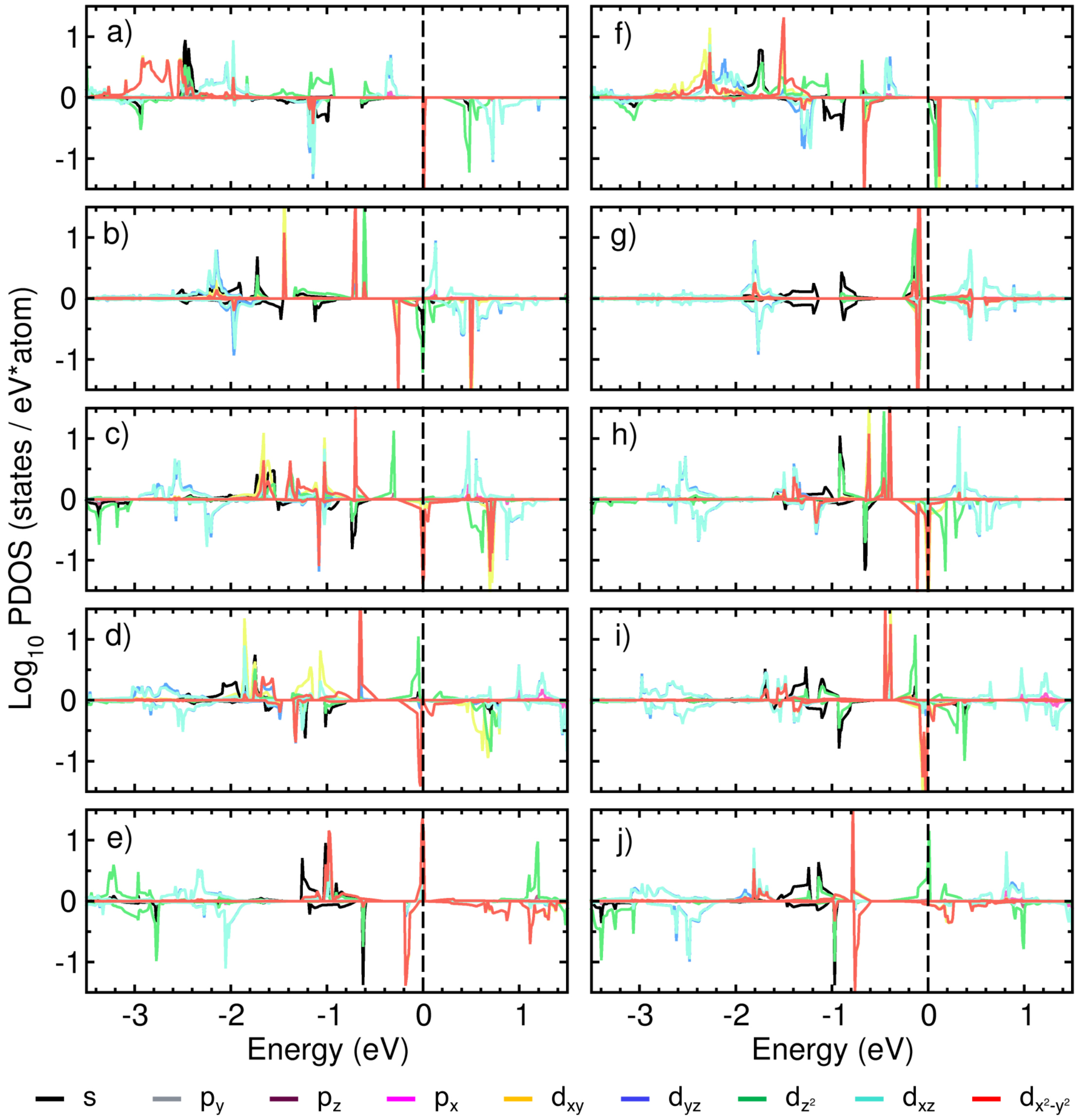


**Figure S21: Projected density of states (PDOS) of the** $TM_T$ **atom in (a)** OsPd@NSV, **(b)** OsPt@NSV, **(c)** OsCo@NSV, **(d)** OsIr@NSV, **(e)** OsMn@NSV, **(f)** IrPd@NSV, **(g)** IrPt@NSV, **(h)** IrCo@NSV, **(i)** IrIr@NSV, **(j)** IrMn@NSV. All spectra are aligned to the Fermi level ($E_F$ = 0).

## S7 Magnetic anisotropy energy

**Table S9: Magnetic properties of single TM atoms adsorbed on a freestanding NSV–graphene sheet.** The initial magnetization direction $\alpha$ (*x*, *xy*, *y*, and *z*). The spin magnetic moment on the TM atom $\mu_S$ and its anisotropy $\Delta\mu_S$, defined as the difference between out-of-plane and in-plane orientations, the corresponding orbital magnetic moment $\mu_L$ and orbital moment anisotropy $\Delta\mu_L$ (all in $\mu_B$). The self-consistent magnetic anisotropy energy MAE (in meV), where positive values correspond to an easy axis perpendicular to the graphene plane.

| Substrate | $\alpha$ | $\mu_S$ | $\Delta\mu_S$ | $\mu_L$ | $\Delta\mu_L$ | MAE |
|---|---|---|---|---|---|---|
| Pd@NSV | *xy*<br>*z* | 0.53<br>0.54 | 0.00 | 0.15<br>0.11 | −0.04 | −0.1 |
| Pt@NSV | *y*<br>*z* | 0.47<br>0.52 | 0.06 | 0.20<br>0.18 | −0.02 | 6.3 |
| Co@NSV | *y*<br>*z* | 1.88<br>1.88 | 0.00 | 0.10<br>0.09 | −0.01 | 0.3 |
| Ir@NSV | *y*<br>*z* | 0.66<br>0.63 | −0.03 | 0.22<br>0.04 | −0.17 | −9.9 |
| Mn@NSV | *xy*<br>*z* | 0.02<br>0.02 | 0.00 | 0.00<br>0.00 | 0.00 | 0.0 |

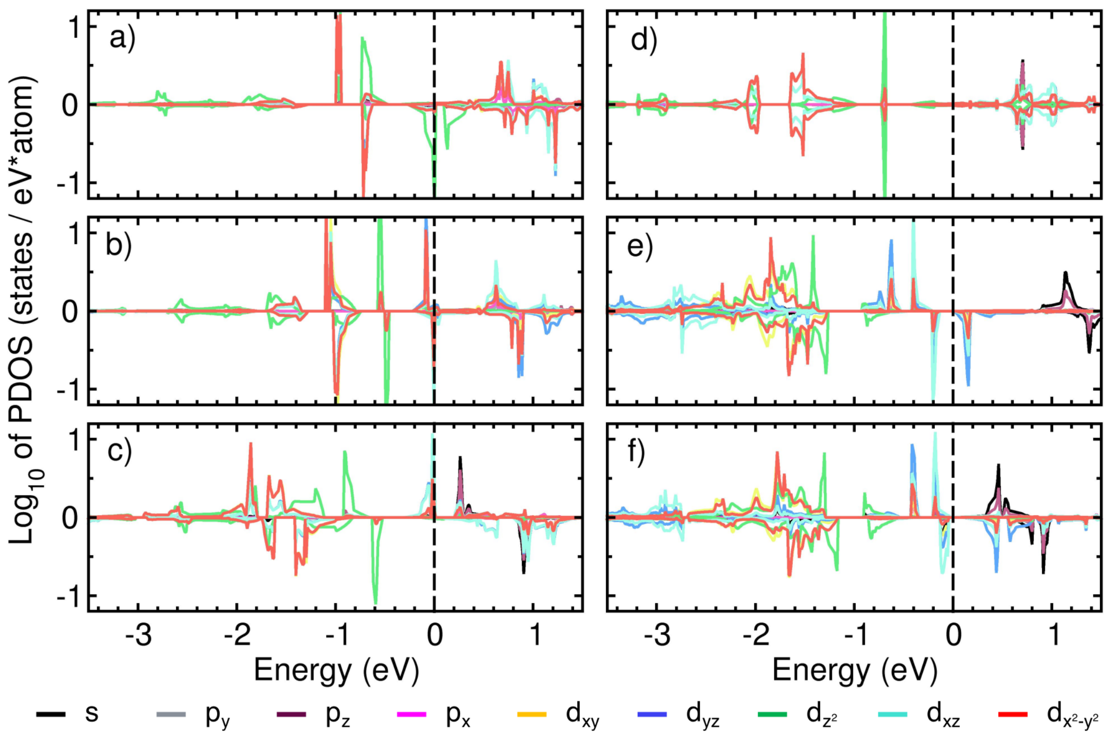


**Figure S22: PDOS of single TM atoms at freestanding NSV. (a)** Mn@NSV, **(b)** Co@NSV, **(c)** Ir@NSV, **(d)** Os@NSV, **(e)** Pd@NSV, **(f)** Pt@NSV. All spectra all aligned to the Fermi level ($E_F$ = 0).

**Table S10: Magnetic properties of single TM atoms adsorbed on a supported NSV–graphene sheet.** The initial magnetization direction $\alpha$ ($x$, $xy$, $y$, and $z$). The spin magnetic moment on the TM atom $\mu_S$ and its anisotropy $\Delta\mu_S$, defined as the difference between out-of-plane and in-plane orientations, the corresponding orbital magnetic moment $\mu_L$ and orbital moment anisotropy $\Delta\mu_L$ (all in $\mu_B$). The self-consistent magnetic anisotropy energy MAE (in meV), where positive values correspond to an easy axis perpendicular to the graphene plane. See Figure S1 for the notation.

| Substrate | $\alpha$ | $\mu_S$ | $\Delta\mu_S$ | $\mu_L$ | $\Delta\mu_L$ | MAE |
|---|---|---|---|---|---|---|
| NSV@Cu(111) | | | | | | |
| CoNSV@Cu(111) | $y$ | 0.00 | 0.00 | 0.00 | 0.00 | 0.0 |
| | $z$ | 0.00 | | 0.00 | | |
| IrNSV@Cu(111) | $y$ | 0.00 | 0.00 | 0.00 | 0.00 | 0.0 |
| | $z$ | 0.00 | | 0.00 | | |
| MnNSV@Cu(111) | $y$ | 4.06 | 0.00 | 0.00 | 0.00 | 0.0 |
| | $z$ | 4.06 | | 0.01 | | |
| PdNSV@Cu(111) | $y$ | 0.00 | 0.00 | 0.00 | 0.00 | 0.0 |
| | $z$ | 0.00 | | 0.00 | | |
| PtNSV@Cu(111) | $y$ | 0.00 | 0.00 | 0.00 | 0.00 | 0.0 |
| | $z$ | 0.00 | | 0.00 | | |
| NSV@Ir(111) | | | | | | |
| IrNSV@Ir(111) | $y$ | 0.00 | 0.00 | 0.00 | 0.00 | 0.0 |
| | $z$ | 0.00 | | 0.00 | | |
| MnNSV@Ir(111) | $x$ | 3.52 | 0.00 | 0.03 | −0.01 | −0.3 |
| | $z$ | 3.52 | | 0.02 | | |
| PdNSV@Ir(111) | $xy$ | 0.22 | −0.21 | 0.05 | −0.05 | −1.0 |
| | $z$ | 0.00 | | 0.00 | | |
| PtNSV@Ir(111) | $y$ | 0.00 | 0.00 | 0.00 | 0.00 | 0.0 |
| | $z$ | 0.00 | | 0.00 | | |
| NSV@MgO(001) | | | | | | |
| CoNSV@MgO(001) | $x$ | 1.87 | 0.00 | 0.10 | −0.01 | 0.2 |
| | $z$ | 1.87 | | 0.09 | | |
| IrNSV@MgO(001) | $x$ | 0.60 | −0.04 | 0.26 | −0.22 | −4.7 |
| | $z$ | 0.56 | | 0.03 | | |
| MnNSV@MgO(001) | $y$ | 4.17 | 0.00 | 0.00 | 0.00 | 0.0 |
| | $z$ | 4.17 | | 0.01 | | |
| PdNSV@MgO(001) | $y$ | 0.52 | 0.00 | 0.15 | −0.05 | 0.0 |
| | $z$ | 0.52 | | 0.10 | | |
| PtNSV@MgO(001) | $xy$ | 0.47 | 0.01 | 0.21 | −0.02 | 1.8 |
| | $z$ | 0.48 | | 0.18 | | |

| Substrate | $\alpha$ | $\mu_S$ | $\Delta\mu_S$ | $\mu_L$ | $\Delta\mu_L$ | MAE |
|---|---|---|---|---|---|---|
| | | | NSV@MgO(111)$_{\text{smE}}$ | | | |
| PtNSV@MgO(111)$_{\text{smE}}$ | $xy$ | 0.98 | 0.01 | 0.29 | −0.06 | −25.2 |
| | $z$ | 0.98 | | 0.23 | | |
| | | | NSV@MgO(111)$_{\text{lmE}}$ | | | |
| CoNSV@MgO(111)$_{\text{lmE}}$ | $xy$ | 2.12 | 0.00 | 0.11 | −0.01 | −0.5 |
| | $z$ | 2.12 | | 0.10 | | |

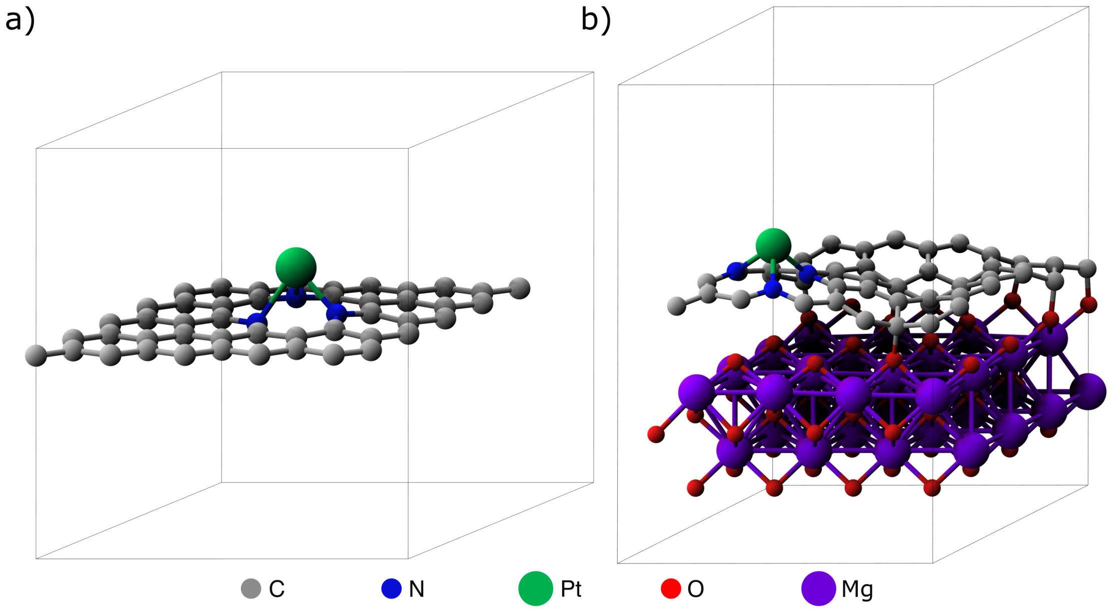


**Figure S23: Pt@NSV**. **(a)** Freestanding and **(b)** MgO(111)-supported. Rendered using POET

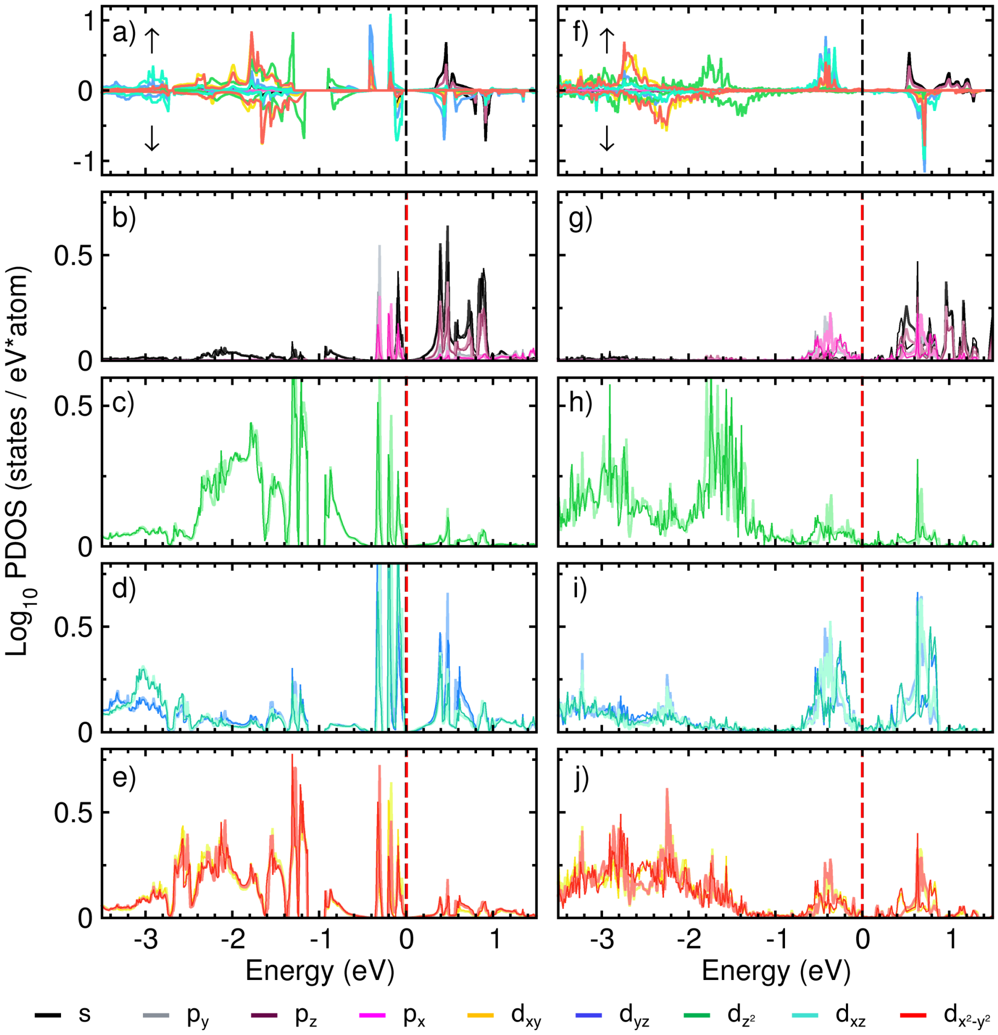


**Figure S24: Projected density of states (PDOS) of the Pt atom in (a–e) freestanding Pt@NSV and (f–j) Pt@NSV@MgO(111)$_{smE}$.** See Figure S1 for the notation. **(a,f)** Scalar-relativistic PDOS, **(b–e,g–j)** NCL-SOC PDOS where the darker thin lines correspond to the magnetization oriented along the out-of-plane *z*-axis, whereas the lighter thick lines indicate magnetization lying within the graphene plane. All spectra are referenced to the Fermi energy ($E_F$) of the out-of-plane magnetization configuration (black dashed line); $E_F$ of the out-of-plane magnetization direction is indicated by the red dashed line.

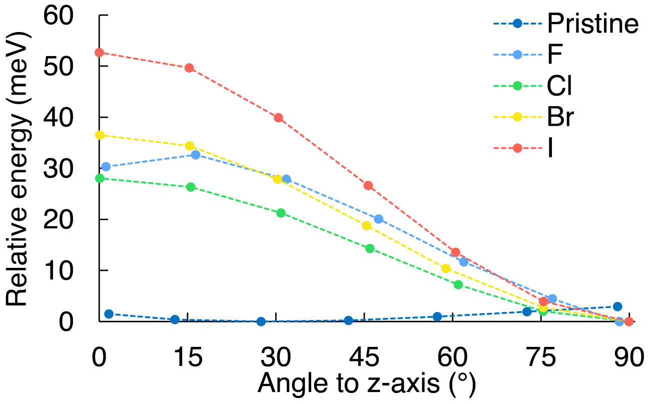


**Figure S25: Energy profiles of pristine and halogenated ($X_1$) freestanding Pt@NSV as a function of the magnetization orientation.** Energies are referenced to the easy axis energy; for the halogenated systems, the easy axis corresponds to magnetization lying parallel to the graphene plane.

**Table S11: Magnetic properties of halogenated single TM atoms adsorbed on a free-standing NSV–graphene sheet.** The initial magnetization direction $\alpha$ ($x$, $xy$, $y$, and $z$). The spin magnetic moment on the TM atom $\mu_S$ and its anisotropy $\Delta\mu_S$, defined as the difference between out-of-plane and in-plane orientations, the corresponding orbital magnetic moment $\mu_L$ and orbital moment anisotropy $\Delta\mu_L$ (in $\mu_B$). The self-consistent magnetic anisotropy energy MAE (in meV), where positive values correspond to an easy axis perpendicular to the graphene plane. $^*$ denotes values obtained by constraining the orientation of the magnetic moment to the specified axis.

| Substrate | $\alpha$ | $\mu_S$ | $\Delta\mu_S$ | $\mu_L$ | $\Delta\mu_L$ | MAE |
|---|---|---|---|---|---|---|
| | | | Pd@NSV | | | |
| $Cl_1Pd$@NSV | $y$<br>$z$ | 0.97<br>0.97 | 0.00 | 0.19<br>0.08 | −0.11 | −4.6 |
| $Br_1Pd$@NSV | $y$<br>$z$ | 0.95<br>0.96 | 0.01 | 0.17<br>0.09 | −0.08 | −7.9 |
| $I_1Pd$@NSV | $x$<br>$z$ | 0.89<br>0.91 | 0.02 | 0.14<br>0.08 | −0.06 | $-18.0^*$ |
| $F_2Pd$@NSV | $xy$<br>$z$ | 0.48<br>0.47 | 0.00 | 0.13<br>0.05 | −0.07 | −0.3 |
| $Cl_2Pd$@NSV | $xy$<br>$z$ | 0.45<br>0.45 | 0.00 | 0.12<br>0.05 | −0.07 | −0.3 |
| $Br_2Pd$@NSV | $xy$<br>$z$ | 0.44<br>0.44 | 0.00 | 0.12<br>0.05 | −0.07 | −0.3 |
| $I_2Pd$@NSV | $xy$<br>$z$ | 0.42<br>0.41 | 0.00 | 0.12<br>0.06 | −0.06 | −0.3 |

| Substrate | $\alpha$ | $\mu_S$ | $\Delta\mu_S$ | $\mu_L$ | $\Delta\mu_L$ | MAE |
|---|---|---|---|---|---|---|
| | | | Pt@NSV | | | |
| $F_1$Pt@NSV | *y*<br>*z* | 0.84<br>0.88 | 0.04 | 0.30<br>0.16 | −0.15 | −31.3* |
| $Cl_1$Pt@NSV | *y*<br>*z* | 0.89<br>0.89 | 0.00 | 0.35<br>0.21 | −0.14 | −28.1* |
| $Br_1$Pt@NSV | *y*<br>*z* | 0.87<br>0.88 | 0.01 | 0.35<br>0.21 | −0.14 | −36.5* |
| $I_1$Pt@NSV | *y*<br>*z* | 0.81<br>0.85 | 0.03 | 0.33<br>0.21 | −0.13 | −52.7 |
| $F_2$Pt@NSV | *xy*<br>*z* | 0.48<br>0.45 | −0.03 | 0.30<br>0.10 | −0.20 | −0.5 |
| $Br_2$Pt@NSV | *xy*<br>*z* | 0.46<br>0.43 | −0.03 | 0.30<br>0.11 | −0.19 | −0.5 |
| $I_2$Pt@NSV | *xy*<br>*z* | 0.44<br>0.41 | −0.03 | 0.29<br>0.11 | −0.18 | −0.5 |
| | | | Co@NSV | | | |
| $F_1$Co@NSV | *y*<br>*z* | 2.38<br>2.38 | 0.00 | 0.08<br>0.09 | 0.00 | 1.0 |
| $Cl_1$Co@NSV | *y*<br>*z* | 2.30<br>2.30 | 0.00 | 0.09<br>0.09 | 0.00 | 0.8 |
| $Br_1$Co@NSV | *xy*<br>*z* | 2.29<br>2.29 | 0.00 | 0.10<br>0.10 | 0.00 | −0.3 |
| $I_1$Co@NSV | *x*<br>*z* | 2.26<br>2.26 | 0.00 | 0.10<br>0.11 | 0.00 | −1.9 |
| $Cl_2$Co@NSV | *x*<br>*z* | 1.39<br>1.39 | 0.00 | 0.07<br>0.07 | 0.00 | −0.2 |
| $Br_2$Co@NSV | *x*<br>*z* | 1.41<br>1.41 | 0.00 | 0.09<br>0.09 | 0.00 | 0.1 |
| $F_3$Co@NSV | *y*<br>*z* | 0.46<br>0.46 | 0.00 | 0.04<br>0.00 | −0.04 | −0.3 |
| $Cl_3$Co@NSV | *y*<br>*z* | 0.39<br>0.39 | 0.00 | 0.04<br>0.00 | −0.03 | −0.2 |
| $Br_3$Co@NSV | *y*<br>*z* | 0.38<br>0.37 | 0.00 | 0.06<br>0.01 | −0.05 | −0.1 |
| $I_3$Co@NSV | *y*<br>*z* | 0.03<br>0.05 | 0.01 | 0.01<br>0.02 | 0.01 | −0.2 |

| Substrate | $\alpha$ | $\mu_S$ | $\Delta\mu_S$ | $\mu_L$ | $\Delta\mu_L$ | MAE |
|---|---|---|---|---|---|---|
| | | | Ir@NSV | | | |
| $F_1Ir@NSV$ | *x*<br>*z* | 0.22<br>0.23 | 0.02 | 0.04<br>0.05 | 0.01 | 0.2 |
| $Cl_1Ir@NSV$ | *xy*<br>*z* | 0.19<br>0.24 | 0.05 | 0.02<br>0.06 | 0.04 | −0.1 |
| $Br_1Ir@NSV$ | *x*<br>*z* | 0.17<br>0.24 | 0.07 | 0.02<br>0.06 | 0.04 | −0.2 |
| $I_1Ir@NSV$ | *x*<br>*z* | 0.17<br>0.25 | 0.09 | 0.04<br>0.06 | 0.02 | 0.0 |
| $F_3Ir@NSV$ | *x*<br>*z* | 0.41<br>0.36 | −0.05 | 0.12<br>0.06 | −0.07 | 1.5 |
| $Cl_3Ir@NSV$ | *y*<br>*z* | 0.36<br>0.31 | −0.05 | 0.12<br>0.06 | −0.07 | 0.9 |
| $Br_3Ir@NSV$ | *y*<br>*z* | 0.33<br>0.28 | −0.05 | 0.14<br>0.05 | −0.08 | 0.7 |
| $I_3Ir@NSV$ | *y*<br>*z* | 0.29<br>0.24 | −0.05 | 0.16<br>0.04 | −0.11 | 0.4 |
| | | | Mn@NSV | | | |
| $F_1Mn@NSV$ | *y*<br>*z* | 1.13<br>1.13 | 0.00 | 0.02<br>0.01 | −0.01 | 0.0 |
| $Cl_1Mn@NSV$ | *y*<br>*z* | 1.15<br>1.15 | 0.00 | 0.01<br>0.01 | −0.01 | 0.0 |
| $Br_1Mn@NSV$ | *y*<br>*z* | 1.16<br>1.16 | 0.00 | 0.00<br>0.00 | 0.00 | −0.1 |
| $I_1Mn@NSV$ | *y*<br>*z* | 1.17<br>1.16 | 0.00 | 0.02<br>0.01 | −0.01 | −0.3 |
| $F_2Mn@NSV$ | *x*<br>*z* | 3.44<br>3.44 | 0.00 | 0.02<br>0.01 | −0.01 | −0.4 |
| $Br_2Mn@NSV$ | *x*<br>*z* | 3.50<br>3.50 | 0.00 | 0.03<br>0.01 | −0.01 | −0.6 |
| $I_2Mn@NSV$ | *x*<br>*z* | 3.56<br>3.56 | 0.00 | 0.07<br>0.03 | −0.04 | −0.8 |
| $F_3Mn@NSV$ | *y*<br>*z* | 2.64<br>2.64 | 0.00 | 0.02<br>0.02 | 0.00 | −0.1 |
| $Br_3Mn@NSV$ | *x*<br>*z* | 2.75<br>2.75 | 0.00 | 0.03<br>0.05 | 0.02 | 0.5 |
| $I_3Mn@NSV$ | *y*<br>*z* | 2.96<br>2.94 | −0.01 | 0.07<br>0.11 | 0.04 | −0.5 |

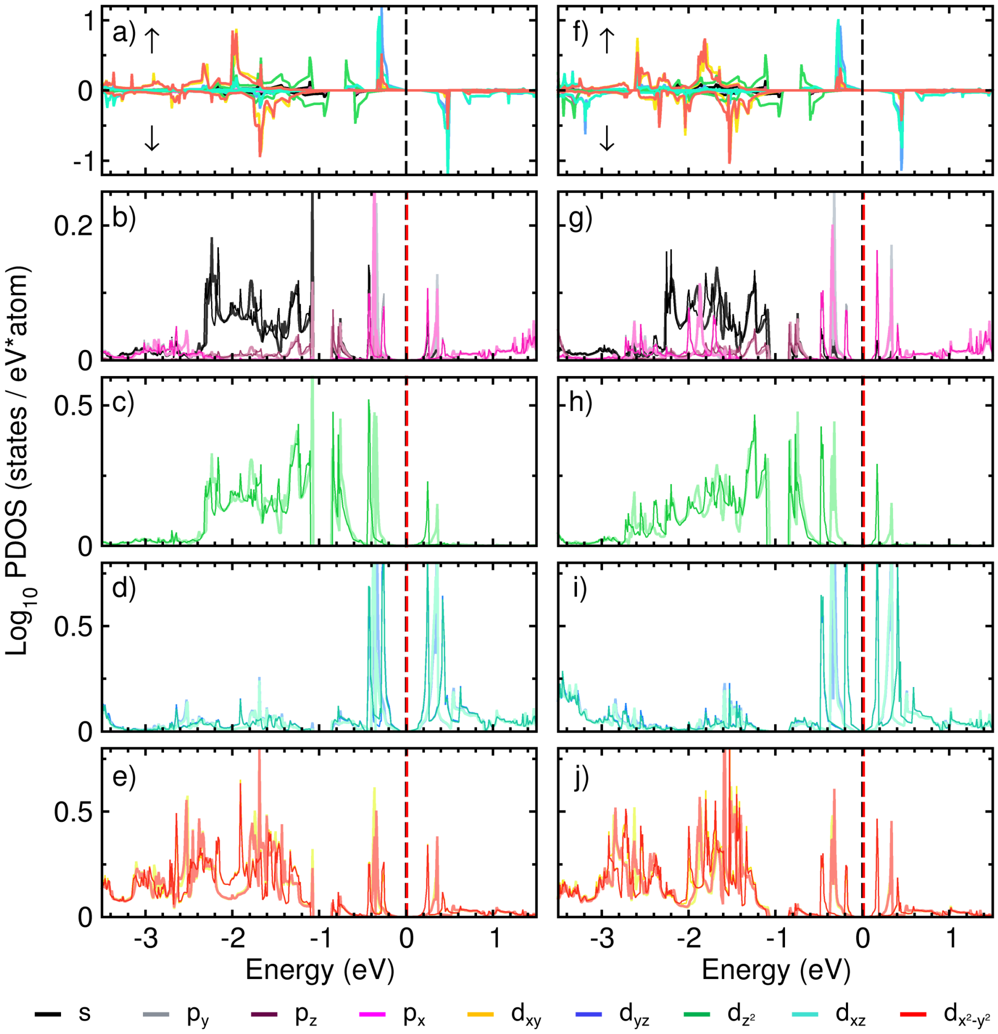


**Figure S26: Projected density of states (PDOS) of the Pt atom in (a–e) freestanding Cl–Pt@NSV and (f–j) freestanding I–Pt@NSV. (a,f)** Scalar-relativistic PDOS, **(b–e,g–j)** NCL-SOC PDOS where the darker thin lines correspond to the magnetization oriented along the out-of-plane *z*-axis, whereas the lighter thick lines indicate magnetization lying within the graphene plane. All spectra are referenced to the Fermi energy ($E_F$) of the out-of-plane magnetization configuration (black dashed line); $E_F$ of the out-of-plane magnetization direction is indicated by the red dashed line.

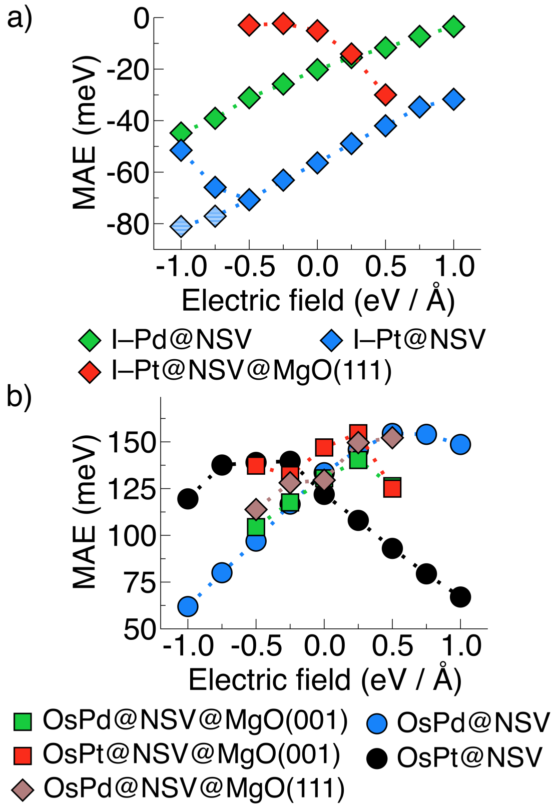


**Figure S27: Electric-field dependence of the magnetic anisotropy energy. (a)** Freestanding I–Pd@NSV and I–Pt@NSV, and I–Pt@NSV@MgO(111). The hard axis corresponds to the $z$ direction, parallel to the I–TM bond. For I–Pt@NSV at electric fields of $-1.00$ and $-0.75$ eV/Å, the system favors a demagnetized state over a magnetized configuration along $z$ (hatched symbols). **(b)** Freestanding OsPd@NSV and OsPt@NSV, and the corresponding systems supported on MgO(001) and MgO(111).

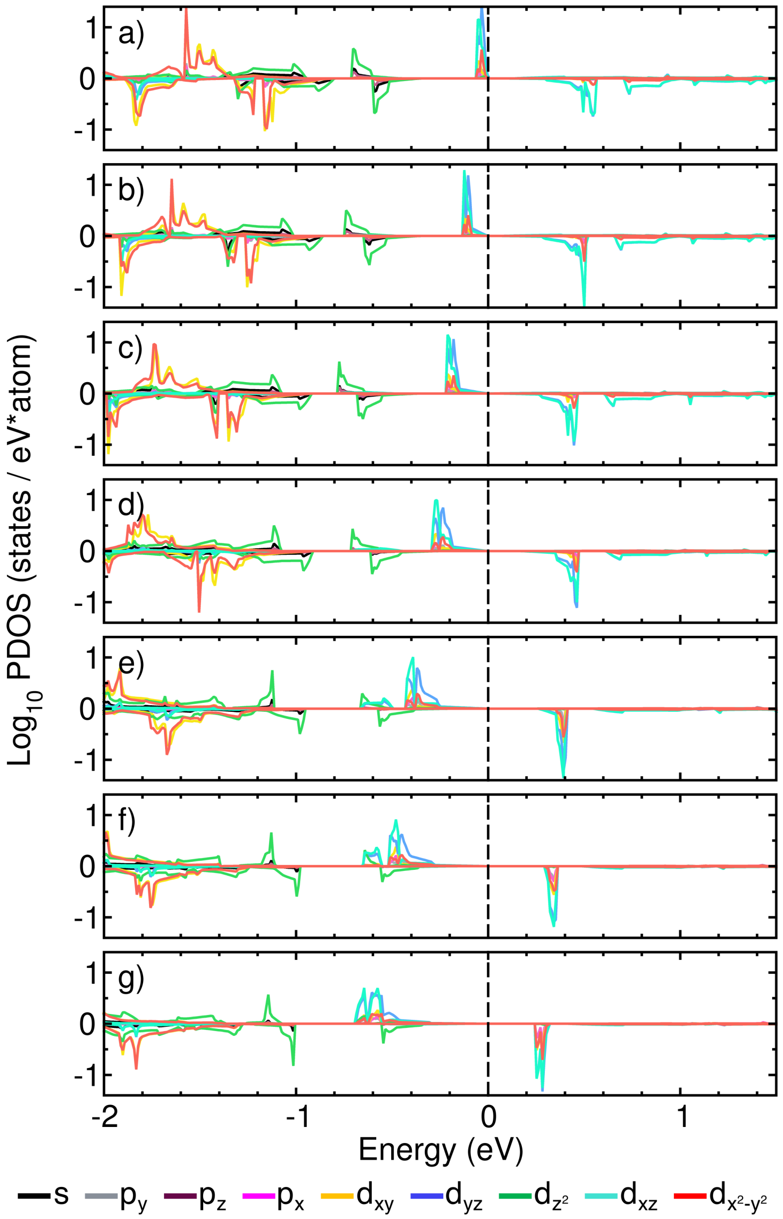


**Figure S28: Projected density of states (PDOS) of the Pt atom in freestanding I–Pt@NSV under an applied external electric field.** The considered fields are **(a)** −1.00 eV/Å, **(b)** −0.75 eV/Å, **(c)** −0.50 eV/Å, **(d)** 0.00 eV/Å, **(e)** +0.50 eV/Å, **(f)** +0.75 eV/Å, and **(g)** +1.00 eV/Å. All spectra are referenced to the Fermi level ($E_F$ = 0).

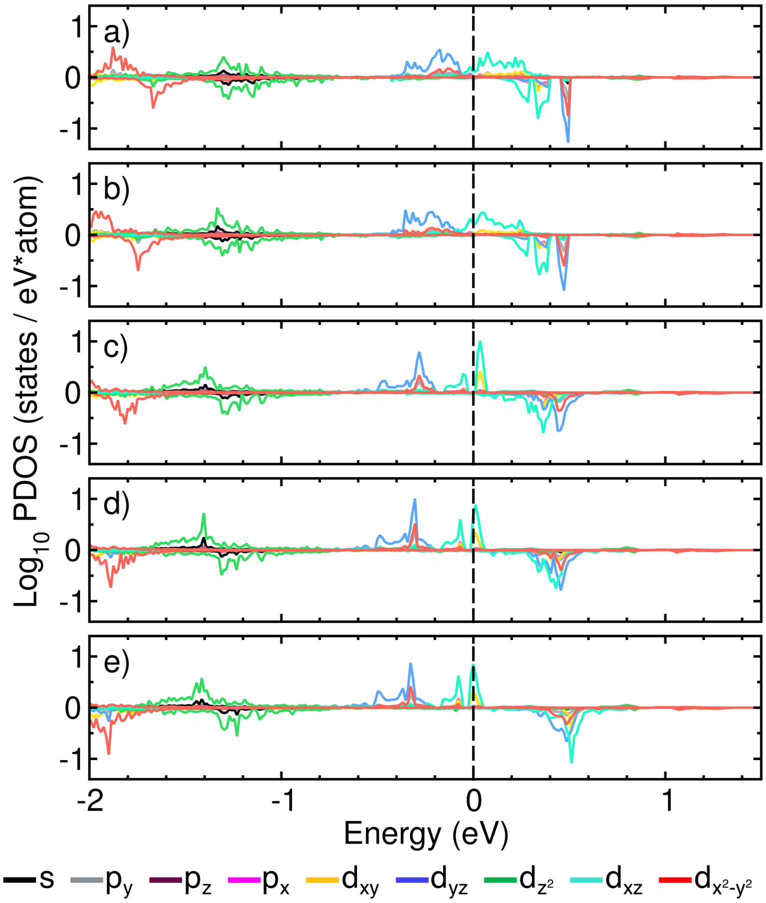


**Figure S29: Projected density of states (PDOS) of the Pt atom in I–Pt@NSV@MgO(111)$_{smE}$ under an applied external electric field.** See Figure S1 for the notation. The considered fields are **(a)** −0.50 eV/Å, **(b)** −0.25 eV/Å, **(c)** 0.00 eV/Å, **(d)** +0.25 eV/Å, **(e)** +0.50 eV/Å. All spectra are referenced to the Fermi level ($E_F$ = 0).

**Table S12: Magnetic properties of TM dimers formed on a freestanding and supported NSV–graphene sheet.** The initial magnetization direction $\alpha$ ($x$, $xy$, $y$, and $z$). The spin magnetic moment on the $TM_B$ atom $\mu_{S,TM_B}$, its anisotropy $\Delta\mu_{S,TM_B}$, defined as the difference between out-of-plane and in-plane orientations (all in $\mu_B$), and the analogous properties of the $TM_T$ atom ($\mu_{S,TM_T}$ and $\Delta\mu_{S,TM_T}$), the corresponding orbital magnetic moment ($\mu_{L,TM_B}$ and $\mu_{L,TM_T}$), and orbital moment anisotropy ($\Delta\mu_{L,TM_B}$ and $\Delta\mu_{L,TM_T}$). The self-consistent magnetic anisotropy energy MAE (in meV), where positive values correspond to an easy axis perpendicular to the graphene plane. See Figure S1 for the notation.

| Substrate | $\alpha$ | $\mu_{S,TM_B}$ | $\Delta\mu_{S,TM_B}$ | $\mu_{S,TM_T}$ | $\Delta\mu_{S,TM_T}$ | $\mu_{L,TM_B}$ | $\Delta\mu_{L,TM_B}$ | $\mu_{L,TM_T}$ | $\Delta\mu_{L,TM_T}$ | MAE |
|---|---|---|---|---|---|---|---|---|---|---|
| | | | | | OsPd | | | | | |
| NSV | $x$<br>$z$ | 0.75<br>0.52 | −0.23 | 2.98<br>2.63 | −0.34 | 0.16<br>0.08 | −0.08 | 0.47<br>0.82 | 0.34 | 133.5 |
| Cu(111) | $y$<br>$z$ | 0.50<br>0.43 | −0.08 | 2.91<br>2.81 | −0.10 | 0.08<br>0.01 | −0.07 | 0.75<br>1.12 | 0.37 | 77.3 |
| Ni(111) | $y$<br>$z$ | 0.52<br>0.50 | −0.02 | 2.92<br>2.89 | −0.03 | 0.09<br>0.02 | −0.08 | 0.63<br>1.16 | 0.53 | 75.6 |
| Ir(111) | $xy$<br>$z$ | 0.67<br>0.58 | −0.09 | 3.00<br>2.87 | −0.13 | 0.14<br>0.01 | −0.13 | 0.47<br>0.96 | 0.49 | 76.5 |
| $MgO(111)_{smH}$ | $y$<br>$z$ | 0.37<br>0.37 | 0.00 | 3.04<br>3.07 | 0.03 | 0.06<br>0.01 | −0.05 | 0.79<br>1.16 | 0.37 | 11.7 |
| $MgO(111)_{smT}$ | $xy$<br>$z$ | 0.17<br>0.09 | −0.08 | 2.79<br>2.66 | −0.13 | 0.08<br>0.02 | −0.05 | 0.54<br>0.92 | 0.39 | 83.8 |
| $MgO(111)_{smE}$ | $x$<br>$z$ | 0.04<br>0.12 | 0.08 | 1.94<br>2.27 | 0.33 | 0.09<br>0.00 | −0.08 | 0.21<br>1.25 | 1.04 | 131.5 |
| $MgO(111)_{lmT}$ | $x$<br>$z$ | 0.15<br>0.13 | −0.02 | 2.84<br>2.69 | −0.15 | 0.07<br>0.01 | −0.06 | 0.57<br>0.84 | 0.27 | 54.6 |
| $MgO(111)_{lmE}$ | $x$<br>$z$ | 0.43<br>0.24 | −0.19 | 2.96<br>2.67 | −0.29 | 0.12<br>0.04 | −0.08 | 0.43<br>0.89 | 0.45 | 112.8 |
| MgO(001) | $xy$<br>$z$ | 0.73<br>0.52 | −0.21 | 2.96<br>2.64 | −0.32 | 0.16<br>0.08 | −0.08 | 0.47<br>0.83 | 0.36 | 128.0 |
| | | | | | OsPt | | | | | |
| NSV | $y$<br>$z$ | 0.02<br>0.25 | 0.23 | 1.10<br>1.95 | 0.85 | 0.05<br>0.08 | 0.03 | 0.32<br>1.26 | 0.94 | 120.0 |
| Cu(111) | $x$<br>$z$ | 0.33<br>0.24 | −0.08 | 2.41<br>2.23 | −0.17 | 0.07<br>0.01 | −0.06 | 0.65<br>1.24 | 0.58 | 114.9 |
| Ni(111) | $xy$<br>$z$ | 0.40<br>0.35 | −0.05 | 2.51<br>2.39 | −0.12 | 0.12<br>0.02 | −0.09 | 0.60<br>1.11 | 0.51 | 78.1 |
| Ir(111) | $x$<br>$z$ | 0.45<br>0.38 | −0.07 | 2.50<br>2.34 | −0.16 | 0.13<br>0.05 | −0.08 | 0.44<br>1.05 | 0.61 | 111.5 |
| $MgO(111)_{smH}$ | $x$<br>$z$ | 0.27<br>0.28 | 0.00 | 1.05<br>1.04 | −0.01 | 0.11<br>0.13 | 0.01 | 0.22<br>0.18 | −0.04 | −3.3 |
| $MgO(111)_{smE}$ | $y$<br>$z$ | 0.01<br>0.01 | 0.00 | 1.68<br>1.88 | 0.19 | 0.03<br>0.01 | −0.02 | 0.22<br>1.52 | 1.30 | 72.8 |
| $MgO(111)_{lmH}$ | $x$<br>$z$ | 0.25<br>0.20 | −0.05 | 2.71<br>2.59 | −0.12 | 0.08<br>0.03 | −0.05 | 0.67<br>0.86 | 0.19 | −9.4 |
| MgO(001) | $x$<br>$z$ | 0.27<br>0.28 | 0.01 | 2.02<br>2.10 | 0.08 | 0.16<br>0.06 | −0.09 | 0.36<br>1.24 | 0.88 | 150.9 |

| Substrate | $\alpha$ | $\mu_{S,}$ $_{TM_B}$ | $\Delta\mu_{S,}$ $_{TM_B}$ | $\mu_{S,}$ $_{TM_T}$ | $\Delta\mu_{S,}$ $_{TM_T}$ | $\mu_{L,}$ $_{TM_B}$ | $\Delta\mu_{L,}$ $_{TM_B}$ | $\mu_{L,}$ $_{TM_T}$ | $\Delta\mu_{L,}$ $_{TM_T}$ | MAE |
|---|---|---|---|---|---|---|---|---|---|---|
| | | | | | OsCo | | | | | |
| NSV | *x*<br>*z* | 0.24<br>0.21 | −0.03 | 1.36<br>1.72 | 0.37 | 0.22<br>0.01 | −0.21 | 0.04<br>1.34 | 1.30 | 66.9 |
| Cu(111) | *xy*<br>*z* | 0.30<br>0.27 | −0.04 | 1.35<br>1.75 | 0.39 | 0.26<br>0.00 | −0.26 | 0.07<br>1.31 | 1.24 | 74.1 |
| Ni(111) | *y*<br>*z* | 1.11<br>0.68 | −0.43 | 2.28<br>2.01 | −0.27 | 0.27<br>0.05 | −0.22 | 0.26<br>0.90 | 0.64 | 74.1 |
| MgO(111)$_{smH}$ | *y*<br>*z* | 0.58<br>0.53 | −0.04 | 2.92<br>2.80 | −0.12 | 0.09<br>0.04 | −0.05 | 0.68<br>0.94 | 0.27 | 48.4 |
| MgO(111)$_{smT}$ | *x*<br>*z* | 0.11<br>0.15 | 0.05 | 2.34<br>2.20 | −0.14 | 0.14<br>0.05 | −0.09 | 0.38<br>0.83 | 0.44 | 8.8 |
| MgO(111)$_{smE}$ | *xy*<br>*z* | 0.15<br>0.05 | −0.10 | 2.29<br>2.26 | −0.03 | 0.20<br>0.08 | −0.12 | 0.28<br>0.86 | 0.57 | 28.2 |
| MgO(111)$_{lmT}$ | *xy*<br>*z* | 0.69<br>0.69 | 0.00 | 0.17<br>0.17 | 0.00 | 0.04<br>0.04 | 0.00 | 0.04<br>0.04 | 0.00 | −1.9 |
| MgO(111)$_{lmE}$ | *xy*<br>*z* | 0.03<br>0.08 | 0.04 | 2.22<br>2.18 | −0.04 | 0.19<br>0.10 | −0.09 | 0.28<br>0.90 | 0.61 | 13.3 |
| MgO(001) | *x*<br>*z* | 0.27<br>0.34 | 0.07 | 1.58<br>1.76 | 0.18 | 0.24<br>0.15 | −0.09 | 0.07<br>1.29 | 1.22 | −61.7 |
| | | | | | OsIr | | | | | |
| NSV | *x*<br>*z* | 0.23<br>0.19 | −0.03 | 1.21<br>1.43 | 0.21 | 0.05<br>0.07 | 0.02 | 0.15<br>1.41 | 1.27 | 50.1 |
| Cu(111) | *x*<br>*z* | 0.27<br>0.12 | −0.14 | 1.52<br>1.46 | −0.06 | 0.07<br>0.03 | −0.04 | 0.24<br>1.36 | 1.12 | 111.0 |
| Ni(111) | *xy*<br>*z* | 0.20<br>0.00 | −0.20 | 1.35<br>0.00 | −1.35 | 0.06<br>0.00 | −0.06 | 0.30<br>0.00 | −0.30 | 73.6 |
| Ir(111) | *x*<br>*z* | 0.09<br>0.08 | −0.01 | 2.71<br>2.77 | 0.05 | 0.01<br>0.04 | 0.03 | 0.44<br>0.89 | 0.45 | 36.0 |
| MgO(111)$_{smH}$ | *x*<br>*z* | 0.05<br>0.01 | −0.03 | 1.10<br>1.60 | 0.50 | 0.01<br>0.05 | 0.04 | 0.23<br>1.21 | 0.98 | 69.7 |
| MgO(111)$_{smE}$ | *y*<br>*z* | 0.09<br>0.04 | −0.05 | 1.89<br>1.82 | −0.07 | 0.06<br>0.03 | −0.03 | 0.34<br>0.91 | 0.56 | 3.7 |
| MgO(111)$_{lmT}$ | *y*<br>*z* | 0.03<br>0.04 | 0.01 | 1.96<br>1.98 | 0.02 | 0.04<br>0.08 | 0.04 | 0.41<br>0.52 | 0.11 | −79.4 |
| MgO(111)$_{lmE}$ | *xy*<br>*z* | 0.04<br>0.06 | 0.01 | 1.81<br>1.75 | −0.06 | 0.05<br>0.04 | −0.02 | 0.32<br>0.92 | 0.60 | −7.6 |
| MgO(001) | *xy*<br>*z* | 0.12<br>0.10 | −0.02 | 1.08<br>1.36 | 0.28 | 0.04<br>0.06 | 0.02 | 0.17<br>1.46 | 1.30 | 43.8 |

| Substrate | $\alpha$ | $\mu_{S,}$ $_{TM_B}$ | $\Delta\mu_{S,}$ $_{TM_B}$ | $\mu_{S,}$ $_{TM_T}$ | $\Delta\mu_{S,}$ $_{TM_T}$ | $\mu_{L,}$ $_{TM_B}$ | $\Delta\mu_{L,}$ $_{TM_B}$ | $\mu_{L,}$ $_{TM_T}$ | $\Delta\mu_{L,}$ $_{TM_T}$ | MAE |
|---|---|---|---|---|---|---|---|---|---|---|
| | | | | | OsMn | | | | | |
| NSV | *xy* | 1.06 | 0.13 | 0.55 | −0.16 | 0.09 | 0.34 | 0.00 | 0.11 | −30.9 |
| | *z* | 1.19 | | 0.39 | | 0.43 | | 0.12 | | |
| Cu(111) | *xy* | 0.96 | 0.14 | 0.65 | −0.27 | 0.09 | 0.29 | 0.01 | 0.16 | −42.8 |
| | *z* | 1.10 | | 0.39 | | 0.38 | | 0.17 | | |
| Ni(111) | *xy* | 1.10 | 0.09 | 0.56 | −0.09 | 0.09 | 0.28 | 0.04 | 0.20 | −45.3 |
| | *z* | 1.19 | | 0.47 | | 0.38 | | 0.24 | | |
| Ir(111) | *x* | 2.19 | −0.03 | 2.12 | −0.23 | 0.13 | 0.02 | 0.25 | 0.33 | 51.7 |
| | *z* | 2.16 | | 1.89 | | 0.16 | | 0.59 | | |
| $MgO(111)_{smE}$ | *x* | 1.46 | −0.11 | 0.52 | 0.80 | 0.10 | 0.01 | 0.06 | 0.98 | −60.2 |
| | *z* | 1.35 | | 1.32 | | 0.11 | | 1.04 | | |
| MgO(001) | *y* | 1.34 | 0.17 | 0.65 | −0.40 | 0.13 | 0.19 | 0.65 | −0.16 | −20.3 |
| | *z* | 1.51 | | 0.25 | | 0.32 | | 0.49 | | |
| | | | | | IrPd | | | | | |
| NSV | *x* | 0.77 | 0.06 | 1.75 | 0.22 | 0.08 | −0.01 | 0.64 | 0.83 | 50.9 |
| | *z* | 0.83 | | 1.97 | | 0.07 | | 1.47 | | |
| Cu(111) | *xy* | 0.36 | 0.06 | 1.64 | 0.19 | 0.09 | −0.01 | 1.13 | 0.71 | 29.9 |
| | *z* | 0.41 | | 1.83 | | 0.08 | | 1.84 | | |
| Ni(111) | *y* | 0.46 | −0.46 | 1.78 | −1.78 | 0.07 | −0.07 | 1.15 | −1.15 | 32.2 |
| | *z* | 0.00 | | 0.00 | | 0.00 | | 0.00 | | |
| Ir(111) | *x* | 0.61 | 0.12 | 1.77 | 0.27 | 0.02 | 0.07 | 1.09 | 0.61 | 117.7 |
| | *z* | 0.73 | | 2.04 | | 0.09 | | 1.70 | | |
| $MgO(111)_{smH}$ | *y* | 0.37 | 0.02 | 1.88 | 0.17 | 0.01 | 0.03 | 1.14 | 0.74 | 37.5 |
| | *z* | 0.39 | | 2.05 | | 0.04 | | 1.88 | | |
| $MgO(111)_{lmE}$ | *y* | 0.15 | 0.18 | 1.14 | 0.48 | 0.04 | 0.03 | 0.58 | 0.85 | 87.6 |
| | *z* | 0.33 | | 1.62 | | 0.07 | | 1.44 | | |
| MgO(001) | *x* | 0.70 | 0.07 | 1.69 | 0.24 | 0.06 | 0.00 | 0.65 | 0.80 | 49.8 |
| | *z* | 0.77 | | 1.92 | | 0.06 | | 1.45 | | |
| | | | | | IrPt | | | | | |
| NSV | *xy* | 0.00 | 0.04 | 0.05 | 0.32 | 0.02 | 0.02 | 0.11 | 0.27 | 1.4 |
| | *z* | 0.05 | | 0.37 | | 0.04 | | 0.38 | | |
| Cu(111) | *x* | 0.07 | 0.03 | 0.82 | 0.45 | 0.03 | −0.01 | 0.48 | 1.06 | 46.7 |
| | *z* | 0.10 | | 1.28 | | 0.01 | | 1.55 | | |
| Ni(111) | *xy* | 0.29 | −0.03 | 1.51 | −0.06 | 0.05 | −0.02 | 1.12 | 0.38 | 48.3 |
| | *z* | 0.26 | | 1.45 | | 0.03 | | 1.50 | | |
| Ir(111) | *x* | 0.26 | 0.07 | 1.07 | 0.30 | 0.04 | 0.06 | 0.70 | 0.51 | 42.0 |
| | *z* | 0.33 | | 1.37 | | 0.09 | | 1.21 | | |
| $MgO(111)_{smH}$ | *x* | 0.05 | −0.01 | 0.23 | −0.01 | 0.00 | 0.01 | 0.15 | −0.02 | −4.9 |
| | *z* | 0.04 | | 0.21 | | 0.01 | | 0.13 | | |
| $MgO(111)_{smE}$ | *y* | 0.02 | 0.00 | 0.07 | 0.84 | 0.00 | 0.00 | 0.09 | 1.15 | 23.8 |
| | *z* | 0.01 | | 0.91 | | 0.01 | | 1.24 | | |
| $MgO(111)_{lmT}$ | *x* | 0.04 | 0.00 | 0.71 | 0.52 | 0.02 | 0.00 | 0.40 | 1.22 | −102.8 |
| | *z* | 0.05 | | 1.24 | | 0.02 | | 1.62 | | |
| MgO(001) | *x* | 0.06 | 0.11 | 0.41 | 0.70 | 0.02 | 0.05 | 0.31 | 0.81 | 21.0 |
| | *z* | 0.17 | | 1.11 | | 0.07 | | 1.12 | | |

| Substrate | $\alpha$ | $\mu_S$, $TM_B$ | $\Delta\mu_S$, $TM_B$ | $\mu_S$, $TM_T$ | $\Delta\mu_S$, $TM_T$ | $\mu_L$, $TM_B$ | $\Delta\mu_L$, $TM_B$ | $\mu_L$, $TM_T$ | $\Delta\mu_L$, $TM_T$ | MAE |
|---|---|---|---|---|---|---|---|---|---|---|
| | | | | | IrCo | | | | | |
| NSV | $x$<br>$z$ | 0.35<br>0.45 | 0.10 | 0.28<br>0.87 | 0.59 | 0.05<br>0.04 | −0.01 | 0.06<br>0.87 | 0.82 | 17.7 |
| Cu(111) | $x$<br>$z$ | 0.57<br>0.96 | 0.39 | 0.37<br>1.10 | 0.74 | 0.11<br>0.00 | −0.11 | 0.00<br>0.87 | 0.87 | 55.0 |
| Ni(111) | $x$<br>$z$ | 1.32<br>1.36 | 0.04 | 1.23<br>1.45 | 0.22 | 0.16<br>0.14 | −0.02 | 0.37<br>1.17 | 0.80 | 108.8 |
| $MgO(111)_{smH}$ | $xy$<br>$z$ | 0.47<br>0.48 | 0.02 | 1.66<br>1.85 | 0.20 | 0.02<br>0.02 | 0.00 | 0.86<br>1.79 | 0.93 | 49.7 |
| $MgO(111)_{smT}$ | $y$<br>$z$ | 0.06<br>0.06 | 0.00 | 0.03<br>0.03 | 0.00 | 0.01<br>0.01 | 0.00 | 0.01<br>0.01 | 0.00 | 0.0 |
| $MgO(111)_{smE}$ | $x$<br>$z$ | 0.15<br>0.20 | 0.05 | 0.65<br>1.29 | 0.64 | 0.07<br>0.03 | −0.04 | 0.18<br>1.56 | 1.39 | 62.9 |
| $MgO(111)_{lmH}$ | $y$<br>$z$ | 0.24<br>0.21 | −0.04 | 0.13<br>0.10 | −0.03 | 0.06<br>0.03 | −0.04 | 0.08<br>0.04 | −0.05 | −0.8 |
| $MgO(111)_{lmT}$ | $xy$<br>$z$ | 0.05<br>0.05 | 0.00 | 0.00<br>0.00 | 0.00 | 0.00<br>0.00 | 0.00 | 0.00<br>0.00 | 0.00 | −1.2 |
| $MgO(111)_{lmE}$ | $x$<br>$z$ | 0.17<br>0.18 | 0.01 | 0.58<br>1.28 | 0.70 | 0.08<br>0.02 | −0.05 | 0.29<br>1.54 | 1.24 | 57.1 |
| MgO(001) | $x$<br>$z$ | 2.12<br>1.52 | −0.60 | 1.59<br>1.26 | −0.33 | 0.20<br>0.11 | −0.10 | 0.57<br>0.87 | 0.30 | 84.6 |
| | | | | | IrIr | | | | | |
| NSV | $y$<br>$z$ | 0.30<br>0.31 | 0.01 | 0.48<br>0.78 | 0.30 | 0.06<br>0.06 | 0.00 | 0.15<br>0.68 | 0.52 | 36.3 |
| Cu(111) | $xy$<br>$z$ | 0.26<br>0.30 | 0.04 | 0.42<br>0.81 | 0.38 | 0.06<br>0.05 | −0.01 | 0.13<br>0.73 | 0.59 | 34.6 |
| Ni(111) | $y$<br>$z$ | 0.18<br>0.15 | −0.03 | 0.78<br>0.72 | −0.06 | 0.07<br>0.08 | 0.01 | 0.85<br>0.87 | 0.02 | −20.7 |
| Ir(111) | $xy$<br>$z$ | 0.02<br>0.03 | 0.01 | 1.71<br>1.81 | 0.10 | 0.01<br>0.01 | 0.00 | 0.66<br>1.11 | 0.45 | 25.3 |
| $MgO(111)_{smH}$ | $y$<br>$z$ | 0.02<br>0.03 | 0.01 | 0.23<br>0.89 | 0.66 | 0.00<br>0.04 | 0.04 | 0.14<br>1.03 | 0.90 | 22.4 |
| $MgO(111)_{smE}$ | $x$<br>$z$ | 0.11<br>0.14 | 0.03 | 0.59<br>1.11 | 0.52 | 0.04<br>0.07 | 0.03 | 0.24<br>1.39 | 1.15 | 44.9 |
| $MgO(111)_{lmH}$ | $x$<br>$z$ | 0.15<br>0.15 | 0.00 | 1.22<br>1.44 | 0.22 | 0.04<br>0.04 | 0.00 | 0.78<br>1.62 | 0.84 | 36.9 |
| $MgO(111)_{lmT}$ | $x$<br>$z$ | 0.07<br>0.06 | −0.01 | 0.42<br>1.05 | 0.63 | 0.03<br>0.01 | −0.01 | 0.17<br>1.52 | 1.35 | 35.1 |
| MgO(001) | $y$<br>$z$ | 0.31<br>0.32 | 0.00 | 0.52<br>0.84 | 0.33 | 0.06<br>0.06 | 0.00 | 0.15<br>0.78 | 0.62 | 41.2 |

| Substrate | $\alpha$ | $\mu_{S,}$ $TM_B$ | $\Delta\mu_{S,}$ $TM_B$ | $\mu_{S,}$ $TM_T$ | $\Delta\mu_{S,}$ $TM_T$ | $\mu_{L,}$ $TM_B$ | $\Delta\mu_{L,}$ $TM_B$ | $\mu_{L,}$ $TM_T$ | $\Delta\mu_{L,}$ $TM_T$ | MAE |
|---|---|---|---|---|---|---|---|---|---|---|
| | | | | | IrMn | | | | | |
| NSV | *xy* | 1.81 | −0.03 | 0.30 | 0.12 | 0.21 | 0.09 | 0.10 | 0.32 | −17.4 |
| | *z* | 1.78 | | 0.42 | | 0.30 | | 0.42 | | |
| Cu(111) | *x* | 1.21 | −0.06 | 0.22 | 0.16 | 0.10 | −0.01 | 0.03 | 0.46 | 7.1 |
| | *z* | 1.15 | | 0.38 | | 0.09 | | 0.49 | | |
| Ni(111) | *y* | 0.00 | 1.87 | 0.00 | 0.40 | 0.00 | 0.22 | 0.00 | 0.35 | −29.2 |
| | *z* | 1.87 | | 0.40 | | 0.22 | | 0.35 | | |
| $MgO(111)_{smT}$ | *x* | 2.60 | 0.00 | 0.18 | −0.01 | 0.07 | −0.01 | 0.02 | 0.00 | 1.0 |
| | *z* | 2.59 | | 0.18 | | 0.06 | | 0.02 | | |
| $MgO(111)_{smE}$ | *y* | 1.42 | 0.01 | 0.40 | 0.21 | 0.12 | 0.20 | 0.04 | 0.62 | −9.9 |
| | *z* | 1.43 | | 0.60 | | 0.32 | | 0.66 | | |
| MgO(001) | *x* | 1.71 | −0.06 | 0.31 | 0.11 | 0.19 | 0.06 | 0.08 | 0.35 | −15.9 |
| | *z* | 1.65 | | 0.42 | | 0.25 | | 0.43 | | |

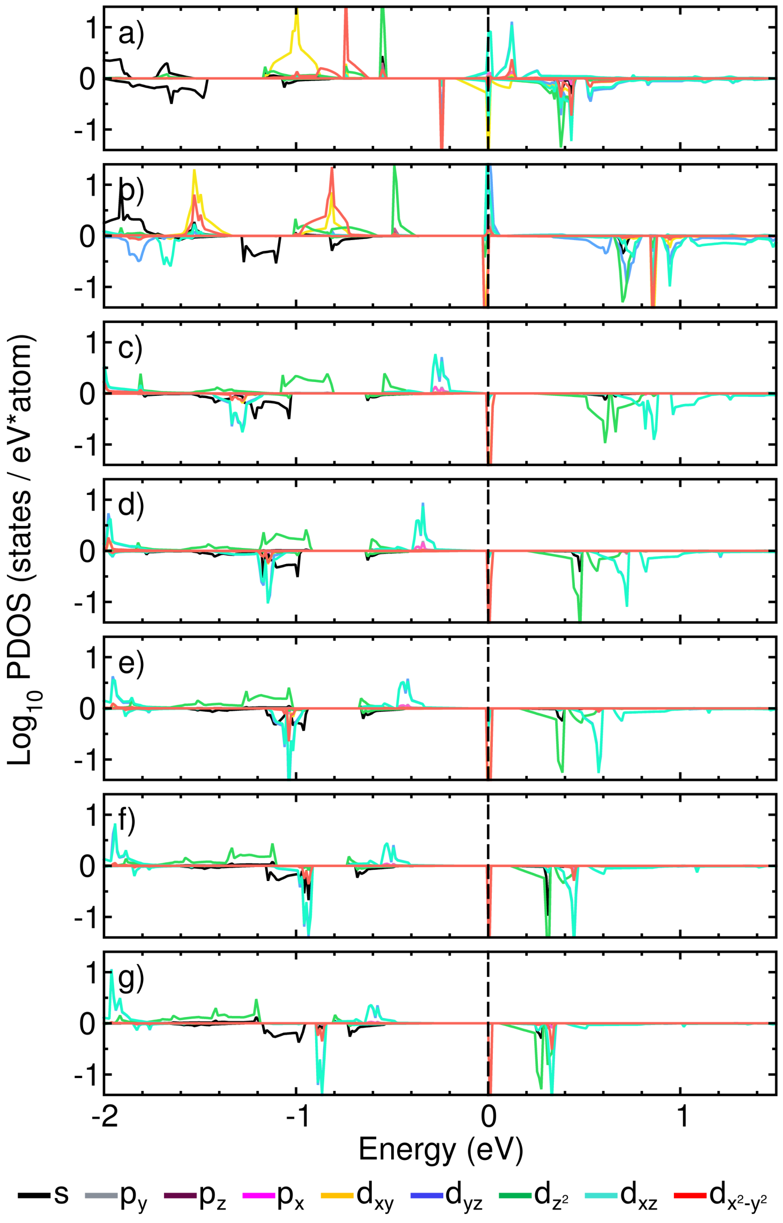


**Figure S30: Projected density of states (PDOS) of freestanding OsPd@NSV as a function of the Os–Pd bond length.** The considered distances are **(a)** 85%, **(b)** 90%, **(c)** 95%, **(d)** 100%, **(e)** 105%, **(f)** 110%, and **(g)** 115% of the optimized equilibrium distance. All spectra are aligned to the Fermi level ($E_F$ = 0).

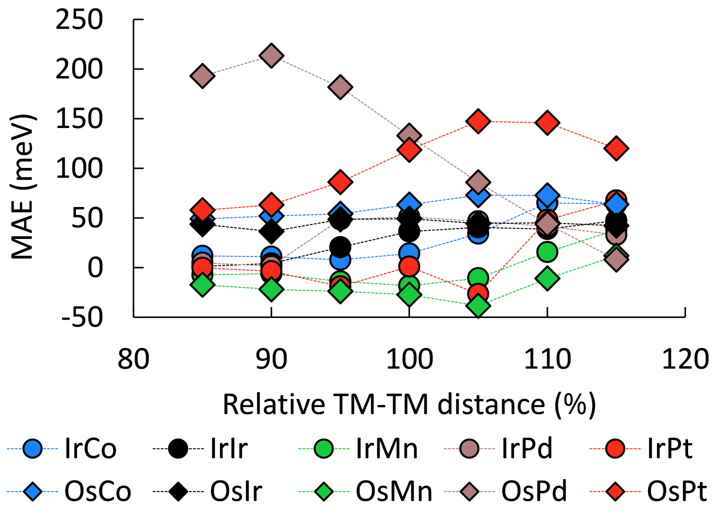


**Figure S31: Magnetic anisotropy energy (MAE) of a TM dimer anchored to the NSV defect in freestanding graphene.** The TM–TM distance is referenced to the equilibrium distance (100%).

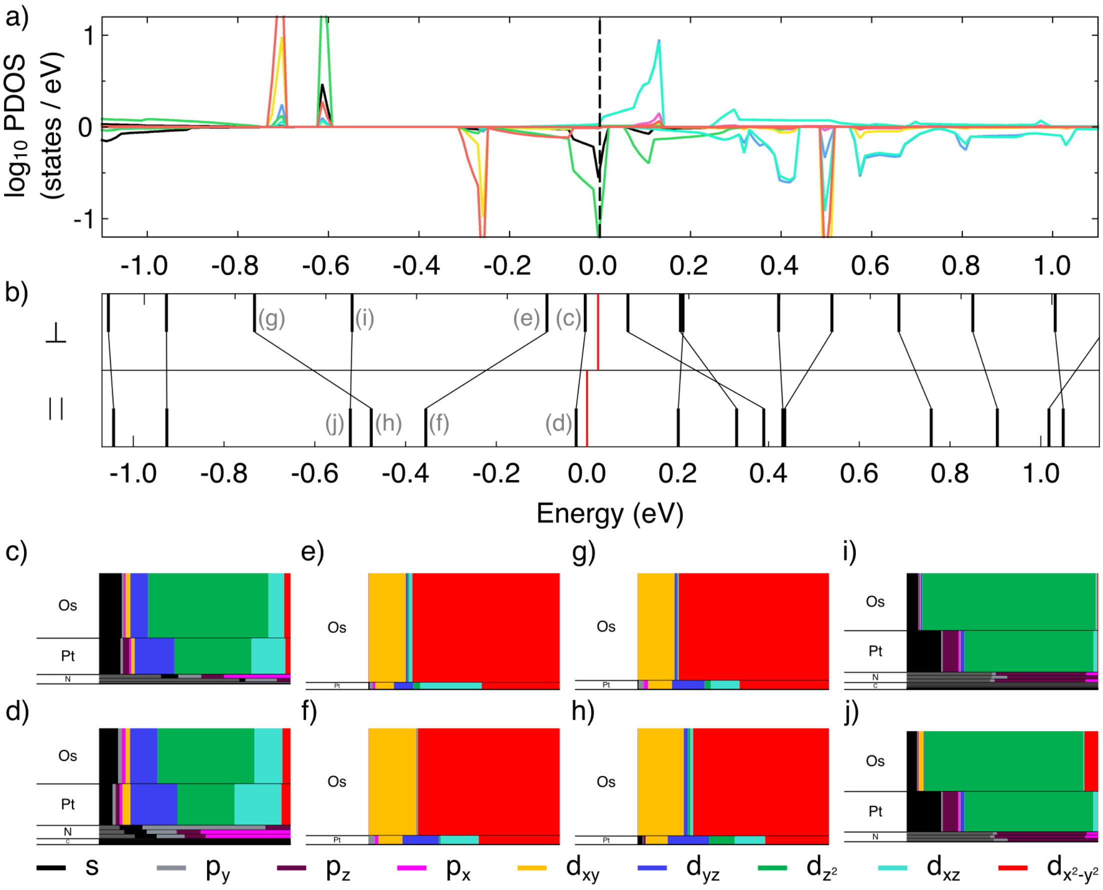


**Figure S32: Origin of the large magnetic anisotropy energy (MAE) in freestanding OsPt@NSV, analyzed via the magnetic force theorem (FT) using POET. (a)** Scalar-relativistic projected density of states (PDOS) of the Os atom. **(b)** Eigenvalue spectra for magnetization perpendicular (top) and parallel (bottom) to the Os–Pt bond, each aligned to its Fermi level ($E_F$, red lines). For a direct comparison, the energy axes are aligned to account for the difference in $E_F$. Letter labels (c)–(j) in panel **(b)** mark the states with the dominant contributions to the MAE, with the corresponding atom- and orbital-resolved decompositions displayed in panles **(c–j)** below, where the box sizes are proportional to the orbital weights.

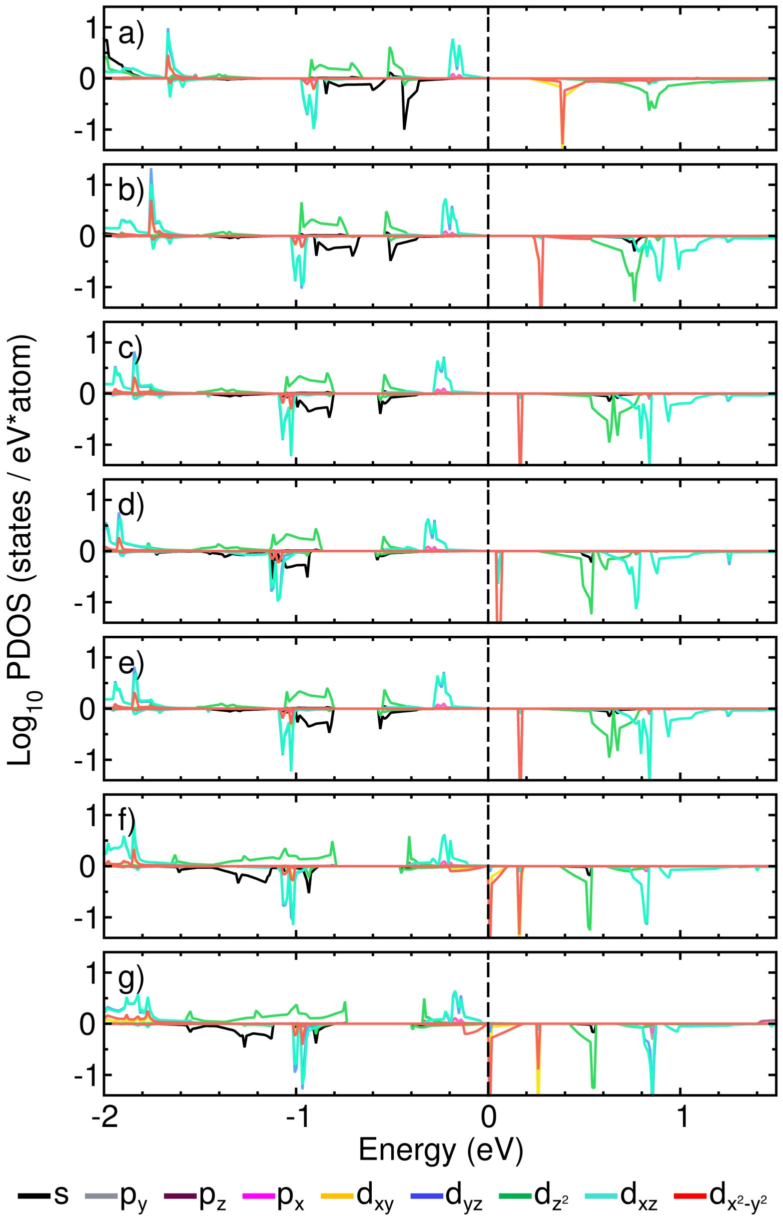


**Figure S33: Projected density of states (PDOS) of the Os atom in freestanding OsPd@NSV under an applied external electric field.** The considered fields are **(a)** −0.75 eV/Å, **(b)** −0.50 eV/Å, **(c)** −0.25 eV/Å, **(d)** 0.00 eV/Å, **(e)** +0.25 eV/Å, **(f)** +0.50 eV/Å, and **(g)** +0.75 eV/Å. All spectra are referenced to the Fermi level ($E_F$ = 0).

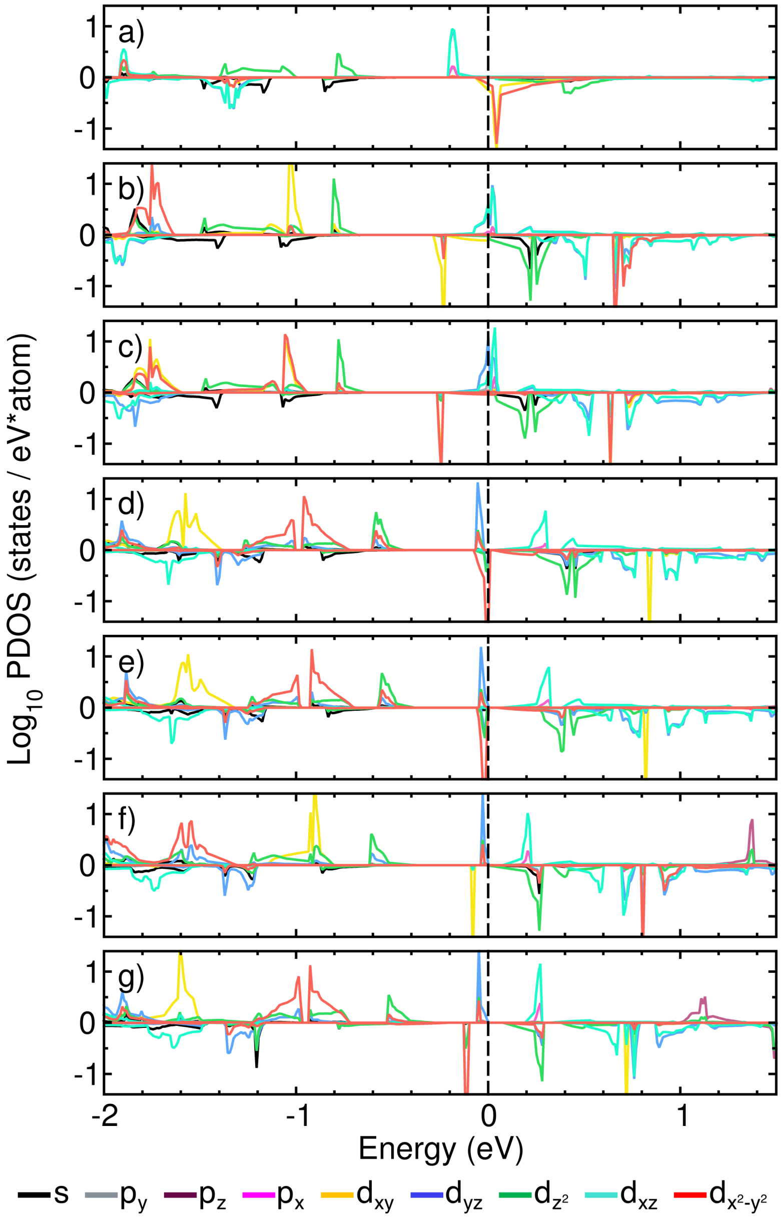


**Figure S34: Projected density of states (PDOS) of the Os atom in freestanding OsPt@NSV under an applied external electric field.** The considered fields are **(a)** −0.75 eV/Å, **(b)** −0.50 eV/Å, **(c)** −0.25 eV/Å, **(d)** 0.00 eV/Å, **(e)** +0.25 eV/Å, **(f)** +0.50 eV/Å, and **(g)** +0.75 eV/Å. All spectra are referenced to the Fermi level ($E_F$ = 0).

## References


1. Błoński, P. & Hafner, J. Cu(1 1 1) supported graphene as a substrate for magnetic dimers with a large magnetic anisotropy: relativistic density-functional calculations. *Journal of Physics: Condensed Matter* **26,** 256001. https://doi.org/10.1088/0953-8984/26/25/256001 (2014).
2. Ryou, J. & Hong, S. First-principles study of carbon atoms adsorbed on MgO(100) related to graphene growth. *Current Applied Physics* **13,** 327–330. https://doi.org/10.1016/j.cap.2012.05.043 (2013).
3. Błoński, P. & Hafner, J. Pt on graphene monolayers supported on a Ni(111) substrate: Relativistic density-functional calculations. *The Journal of Chemical Physics* **136,** 074701. https://doi.org/10.1063/1.3684891 (2012).
4. Cho, S. B. & Chung, Y.-C. Bandgap engineering of graphene by corrugation on lattice-mismatched MgO (111). *Journal of Materials Chemistry C* **1,** 1595. https://doi.org/10.1039/c2tc00257d (2013).
5. Momma, K. & Izumi, F. VESTA 3 for three-dimensional visualization of crystal, volumetric and morphology data. *Journal of Applied Crystallography* **44,** 1272–1276. https://doi.org/10.1107/S0021889811038970 (2011).
6. Bader, R. F. W. Atoms in molecules. *Accounts of Chemical Research* **18,** 9–15. https://doi.org/10.1021/ar00109a003 (1985).
7. Henkelman, G., Arnaldsson, A. & Jónsson, H. A fast and robust algorithm for Bader decomposition of charge density. *Computational Materials Science* **36,** 354–360. https://doi.org/10.1016/j.commatsci.2005.04.010 (2006).
8. Navrátil, J., Błoński, P. & Otyepka, M. Large Magnetic Anisotropy in OsIr Dimer Anchored in Defective Graphene. *Nanotechnology* **32,** 230001. https://doi.org/10.1088/1361-6528/abe966 (2021).
